# Supplementary material for: APOPT1/COA8 assists COX assembly and is oppositely regulated by UPS and ROS
Source: EMBO Mol Med. 2018 Dec 14;11(1):e9582. doi: 10.15252/emmm.201809582 (PMC6328941; doi:10.15252/emmm.201809582)
Supplement: Supplementary file 1 — Appendix [file EMMM-11-e9582-s001.pdf]

APPENDIX

APOPT1/COA8 ASSISTS COX ASSEMBLY AND IS OPPOSITELY REGULATED BY UPS AND ROS.

TABLE OF CONTENTS

Appendix Figure S1 – Characterization of the clinical phenotype of Apopt1 knockout mice 3

Appendix Figure S2 – Biochemical and structural analysis in mouse tissues........................ 4

Appendix Figure S3 – Expression and localization of different APOPT1 isoforms 6

Appendix Figure S4– Cell viability and growth... 8

Appendix Figure S5– APOPT1 detection in mitochondria by WB analysis........................ 10

Appendix Figure S6– APOPT1 detection in mitochondria by fluorescence super-resolution microscopy 11

Appendix Figure S7 – Quantification of mRNA levels 12

Appendix Figure S8 – Ubiquitination analysis of cells stressed with oxidants.................... 13

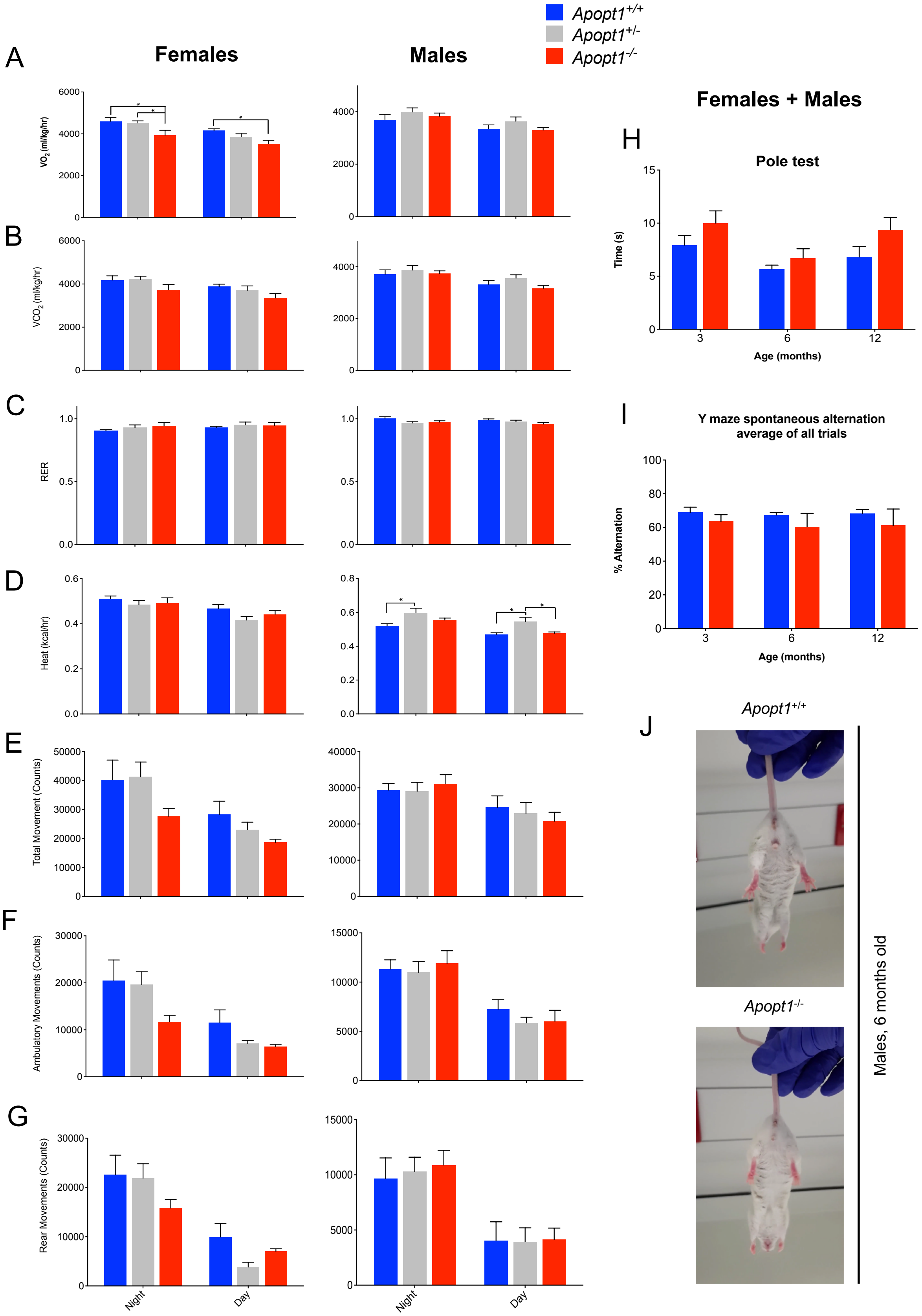

Apopt1+/+
Apopt1+/-
Apopt1-/-
A
Females
Males
VO2 (ml/kg/hr)
B
VCO2 (ml/kg/hr)
C
RER
D
Heat (kcal/hr)
E
Total Movement (Counts)
F
Ambulatory Movements (Counts)
G
Rear Movements (Counts)
Night
Day
Females + Males
H
Pole test
Time (s)
Age (months)
I
Y maze spontaneous alternation average of all trials
% Alternation
Age (months)
J
Apopt1+/+
Apopt1-/-
Males, 6 months old

Appendix Figure S1 – Characterization of the clinical phenotype of Apopt1 knockout mice (Related to main Figure 2)

A Volume of oxygen (ml/kg/hr) consumed in female and male animals at 3 months of age measured in the CLAMS™ system. The asterisks represent the significance levels calculated by two-way ANOVA with Sidak's multiple comparisons test: Females - *P = 0.0190 (WT vs KO, night), *P = 0.0426 (het vs KO, night), *P = 0.0275 (WT vs KO, day).

B Volume of carbon dioxide (ml/kg/hr) produced in female and male animals at 3 months of age measured in the CLAMS™ system.

C Respiratory exchange ratio (RER) in female and male animals at 3 months of age measured in the CLAMS™ system.

D Heat (Kcal/hr) produced by female and male animals at 3 months of age measured in the CLAMS™ system. The asterisks represent the significance levels calculated by two-way ANOVA with Sidak's multiple comparisons test: Males - *P = 0.0127 (WT vs het, night), *P = 0.0140 (WT vs het, day), *P = 0.0148 (het vs KO, day).

E Total movements of female and male animals at 3 months of age measured in the CLAMS™ system.

F Ambulatory movements of female and male animals at 3 months of age measured in the CLAMS™ system.

G Rear movements of female and male animals at 3 months of age measured in the CLAMS™ system.

H Time in seconds spent by the female and male mice to descend a pole at different ages.

I Percentage of alternation scored in the Y maze by the female and male mice at different ages.

J Illustration of the posture of wild-type mice compared to Apopt1$^{-/-}$ mice when the tails were suspended. No feet-clasping was observed.

Data information: In (A-I), data are presented as mean ± SEM (n = 5 mice per group/gender).

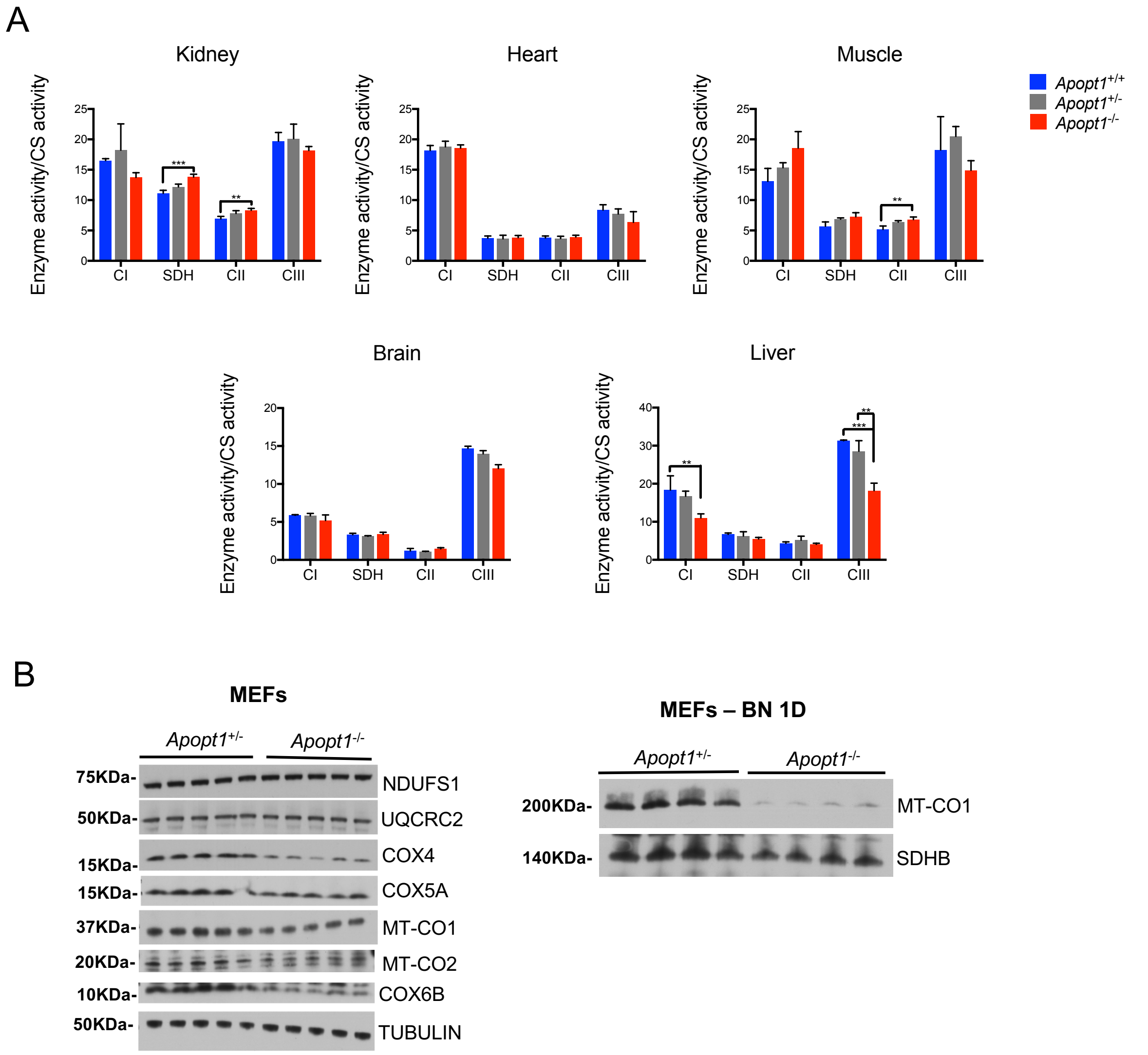

Appendix Figure S2 – Biochemical and structural analysis in mouse tissues (Related to main Figure 3)

A Complex I, succinate dehydrogenase (SDH), complex II and complex III enzymatic activity normalized to the activity of citrate synthase (CS) measured in kidney, heart, skeletal muscle, cerebellar cortex and liver of three animals per genotype at three months of age. Data are presented as mean ± SEM (n = 3 mice per genotype). The asterisks represent the significance levels calculated by two-way ANOVA with Sidak's multiple comparisons test: Complex I - **P = 0.0092 (WT vs KO, liver), SDH - ***P = 0.0007 (WT vs KO, kidney), Complex II - *P = 0.0105 (WT vs KO, muscle), *P = 0.0277 (WT vs KO, kidney), Complex III - **P = 0.0044 (het vs KO, liver), ****P < 0.001 (WT vs KO, liver).
B Left: Western blot analysis of SDS-PAGE of total lysates from mouse embryonic fibroblasts from the indicated genotypes, each lane showing the results for one animal. Right: Western blot analysis of 1D-BNGE of mitochondria from mouse embryonic fibroblasts from the indicated genotypes, each lane showing the results from one animal.

A

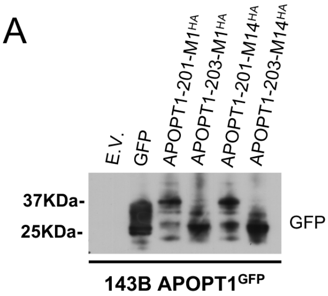

APOPT1 construct	MTS processing	Predicted molecular weight	Predicted molecular weight with the GFP tag
APOPT1-201-M1	Precursor	24.2 kDa	51.2 KDa
	Mature	20.1 KDa	47.1 KDa
APOPT1-201-M14	Precursor	23.0 KDa	50.0 kDa
	Mature	20.1 KDa	47.1 KDa
APOPT1-203-M1	Precursor	14.3 KDa	41.3 KDa
	Mature	10.2 KDa	37.2 KDa
APOPT1-203-M14	Precursor	13.1 KDa	40.1 KDa
	Mature	10.2 KDa	37.2 KDa

B

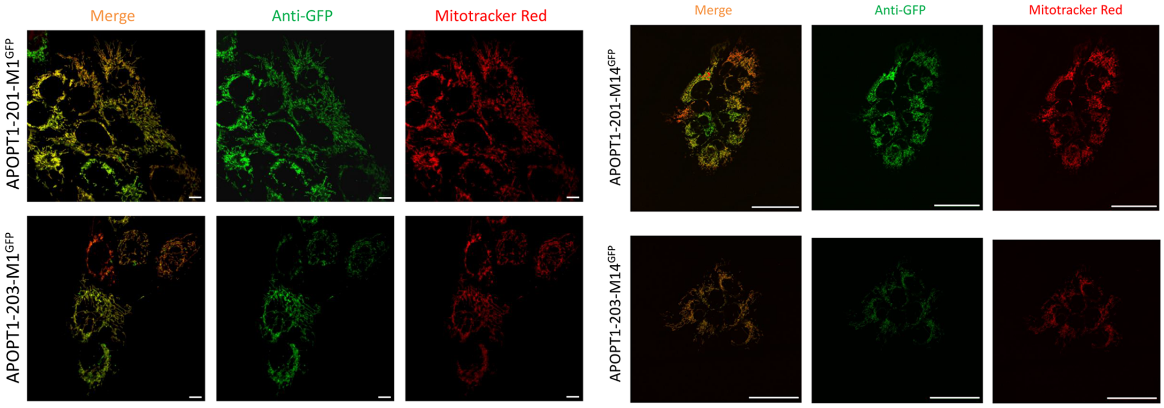

C

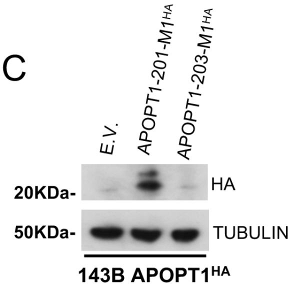

D

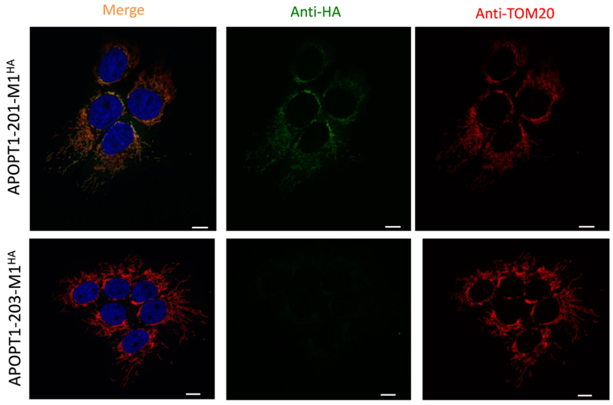

Appendix Figure S3 – Expression and localization of different APOPT1 isoforms (Related to main Figure 4)

A Western blot analysis of SDS-PAGE of total lysates from 143B cells transduced with the empty vector (E.V.), the GFP protein alone or different isoforms of the wild-type APOPT1 protein tagged with GFP (see main text for details). The table shows the predicted molecular weight of the precursor and mature endogenous and GFP-tagged proteins, considering the alternative start codons (M1 and M14) and the two alternative splicing isoforms (201 and 203).

B Localization of APOPT1 by immunofluorescence on fixed cells. The different isoforms of $APOPT1^{GFP}$ overexpressed in 143B cells were detected using Rabbit Anti-GFP (green). The mitochondrial network was visualized using MitoTracker® Red CMXRos (red). Scale bars: 10 µm for $APOPT1\text{-}201/203\text{-}M1^{GFP}$ (left panel) and 50 µm for $APOPT1\text{-}201/203\text{-}M14^{GFP}$ (right panel).

C Western blot analysis of SDS-PAGE of total lysates from 143B cells transduced with the empty vector (E.V.) or different isoforms of the wild-type APOPT1 protein tagged with HA.

D Localization of APOPT1 by immunofluorescence on fixed cells. The different isoforms of $APOPT1^{HA}$ overexpressed in 143B cells were detected using Rat Anti-HA (green). Rabbit Anti-TOM20 (red) was used to visualize the mitochondrial network. Scale bars: 10 µm.

A

HeLa APOPT1-001-M1^{GFP}

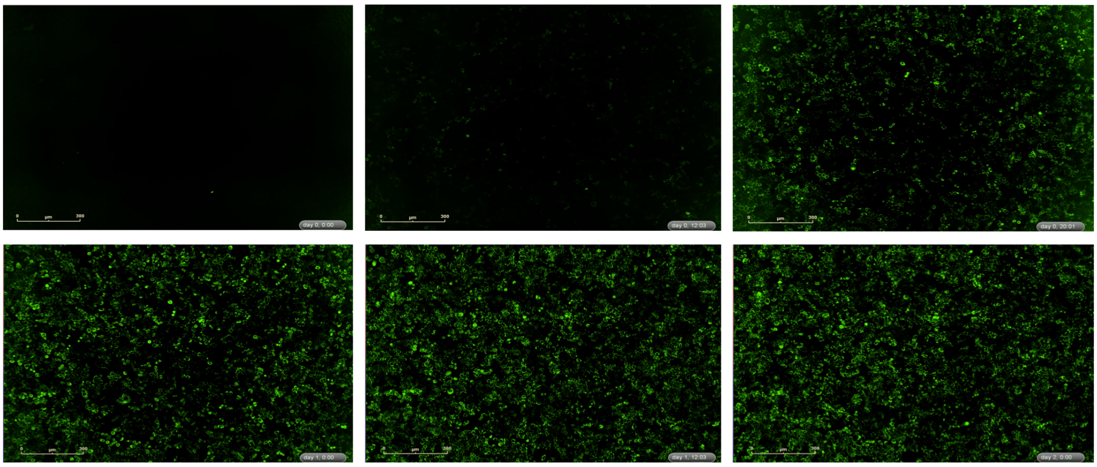

B

143B APOPT1-001-M1^{GFP}

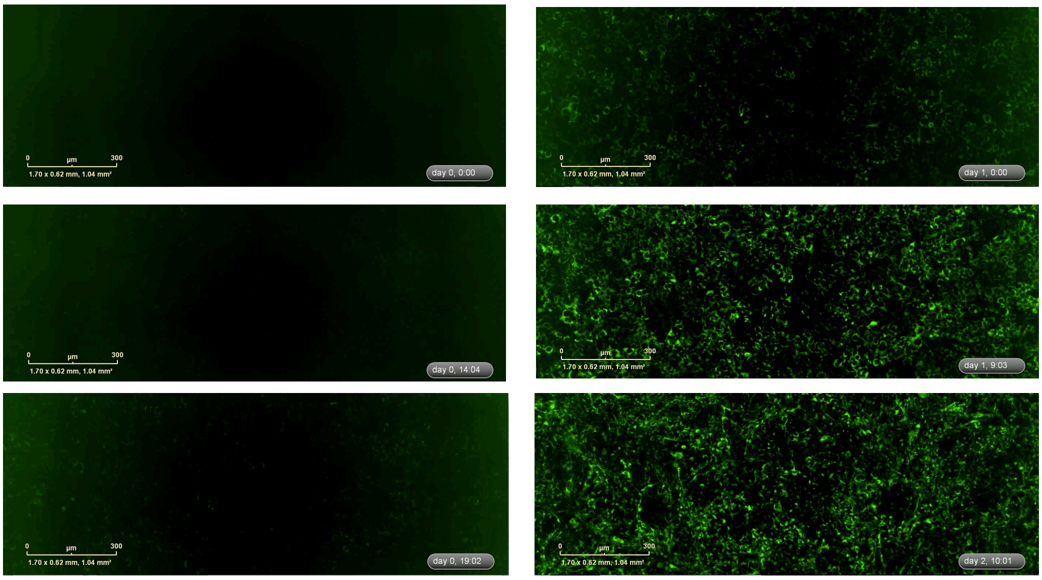

C

143B APOPT1-001-M1^{HA}

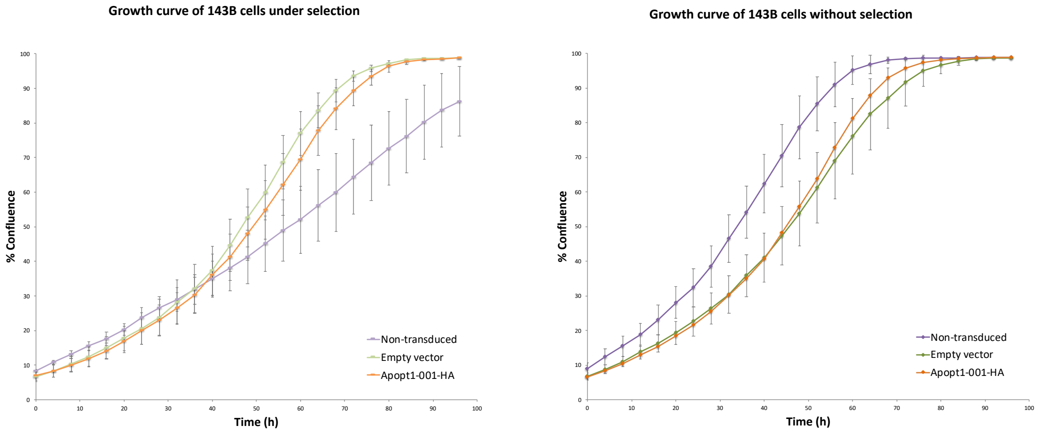

Appendix Figure S4– Cell viability and growth (Related to main Figure 4)

A Live cell fluorescence imaging using an Incucyte ZOOM instrument to monitor APOPT^{GFP} expression in HeLa cells at different time points immediately after transduction.

B Live cell fluorescence imaging using an Incucyte ZOOM instrument to monitor APOPT^{GFP} expression in 143B cells at different time points immediately after transduction.

C Growth curves of 143B non-transduced cells or transduced with the empty vector and with APOPT1^{HA}. The graph on the right shows growth in non-selective conditions and the graph on the left under antibiotic selection with 1 μg/ml puromycin.

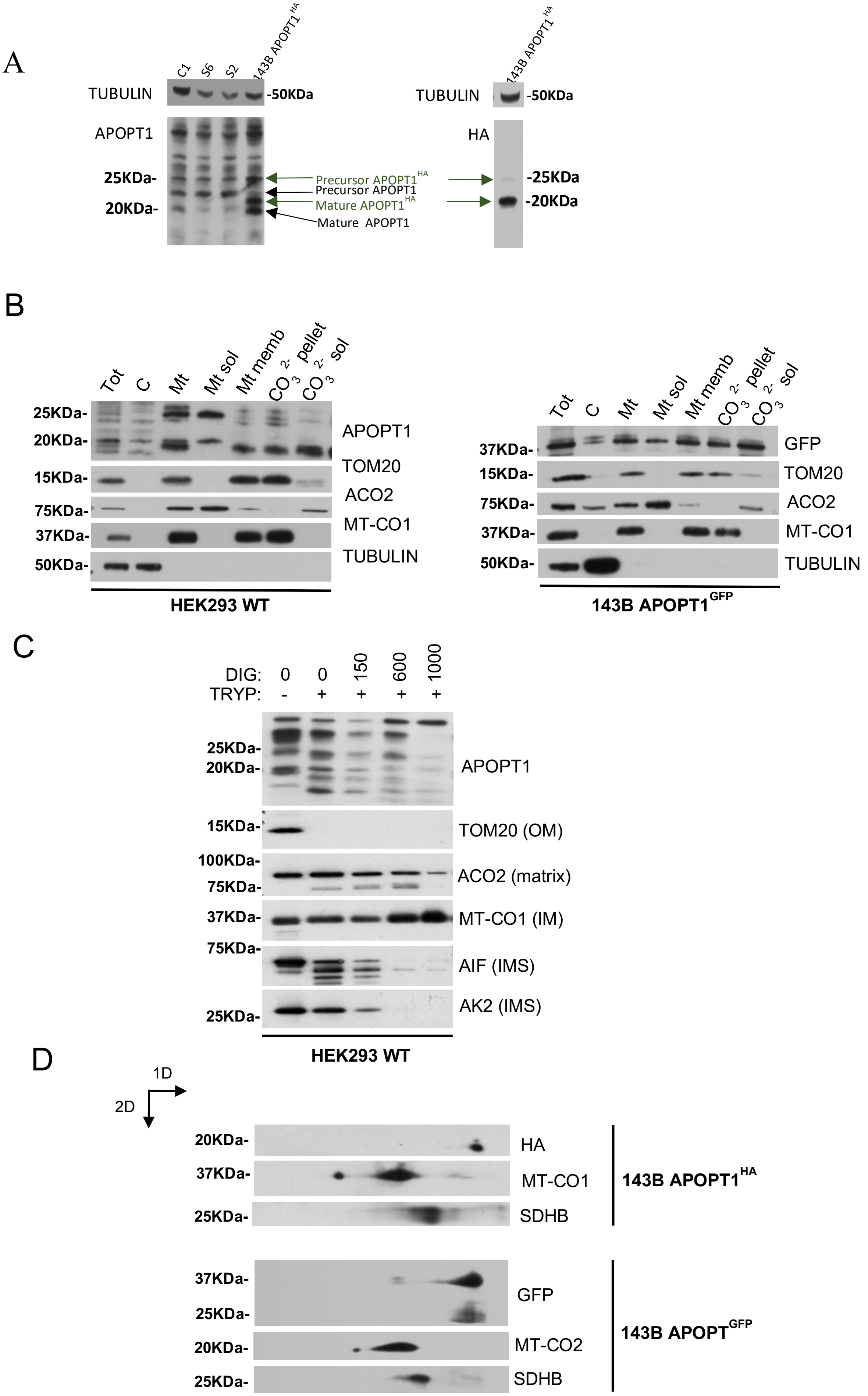
A
C1
S6
S2
143B APOPT1HA
TUBULIN
-50KDa
APOPT1
25KDa-
20KDa-
Precursor APOPT1HA
Precursor APOPT1
Mature APOPT1HA
Mature APOPT1
143B APOPT1HA
TUBULIN
-50KDa
HA
-25KDa
-20KDa
B
Tot
C
Mt
Mt sol
Mt memb
CO3 2- pellet
CO3 2- sol
25KDa-
20KDa-
15KDa-
75KDa-
37KDa-
50KDa-
APOPT1
TOM20
ACO2
MT-CO1
TUBULIN
HEK293 WT
37KDa-
GFP
143B APOPT1GFP
C
DIG: 0 0 150 600 1000
TRYP: - + + + +
25KDa-
20KDa-
APOPT1
15KDa-
TOM20 (OM)
100KDa-
75KDa-
ACO2 (matrix)
37KDa-
MT-CO1 (IM)
75KDa-
AIF (IMS)
25KDa-
AK2 (IMS)
HEK293 WT
D
1D
2D
20KDa-
HA
37KDa-
MT-CO1
25KDa-
SDHB
143B APOPT1HA
37KDa-
25KDa-
GFP
20KDa-
MT-CO2
25KDa-
SDHB
143B APOPTGFP

Appendix Figure S5– APOPT1 detection in mitochondria by WB analysis (Related to main Figure 4)

A Western blot analysis of SDS-PAGE of total lysates from control immortalized human skin fibroblasts (C1), two APOPT1-deficient patients (S6 and S2) and 143B cells overexpressing wild-type $APOPT1^{HA}$. The left blot was immunodetected with an antibody raised against the full-length human APOPT1. The right blot was immunodetected with anti-HA.

B Western blot of SDS-PAGE of different fractions from the indicated cell lines. Tot: total lysate. C: post-mitochondrial fraction (cytoplasm). Mt: isolated mitochondria. Mt sol: Soluble mitochondrial fraction. Mt memb: mitochondrial membranes. CO_3^{2-} pellet: Pellet after carbonate extraction with 0.1 M Na_2CO_3, pH 10.5 for 30 minutes. CO_3^{2-} sol: soluble fraction after the carbonate extraction.

C Western blot of SDS-PAGE of mitochondria used for protease protection assay on HEK293T cells. The experiment was carried out in isolated mitochondria exposed to increasing amounts of digitonin (expressed in µg) and 50 µg/ml trypsin. TOM20: Translocase of the outer membrane 20 kDa. ACO2: Aconitase 2 (mitochondrial isoform). AIF: Apoptosis inducing factor. AK2: Adenylate kinase 2. OM: outer mitochondrial membrane. IM: inner mitochondrial membrane. IMS: intermembrane space.

D Western blot of 2D-BNGE of mitoplasts from the indicated cell lines. SDHB: succinate dehydrogenase subunit B used as a normalization and molecular weight standard signal.

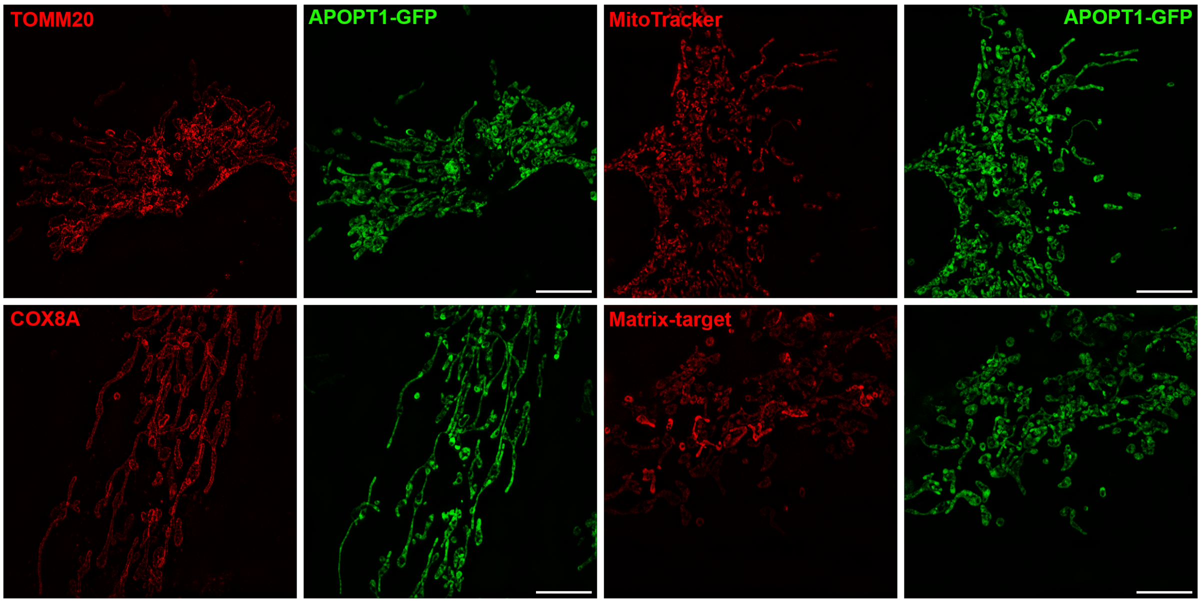

Appendix Figure S6– APOPT1 detection in mitochondria by fluorescence super-resolution microscopy (Related to main Figure 4)
N-SIM super-resolution micrographs showing 0.8 µm Maximum Intensity Projection (0.15 µm for each Z-stack) of 143B cells expressing APOPT1^{GFP} shown in Green, TOMM20 (outer membrane), COX8A (inner membrane), MitoTracker (inner membrane and matrix) and matrix-target (mScarlet) shown in Red.

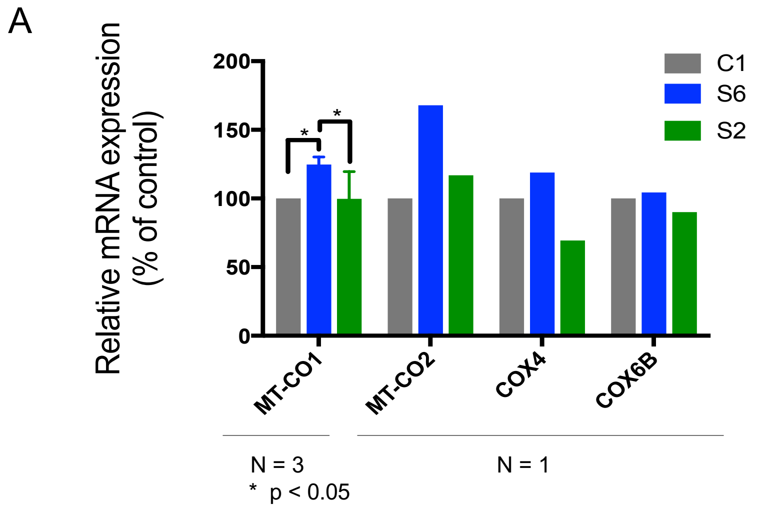

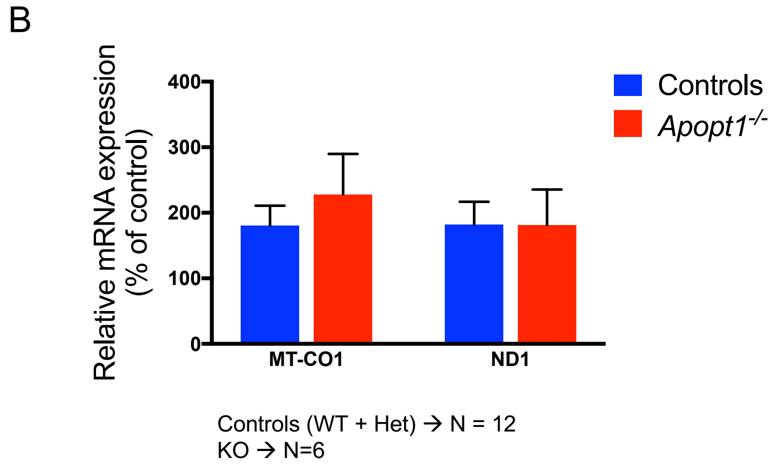

Appendix Figure S7 – Quantification of mRNA levels (Related to main Figure 5)
A Relative mRNA expression of different COX (CIV) subunits normalized to the expression of GAPDH in control immortalized human skin fibroblasts (C1) and two APOPT1-deficient patients (S6 and S2). MT-CO1 expression was measured in three biological replicas and MT-CO2, COX4 and COX6B was measured in a single sample. Data are presented as mean ± SEM (n = 3 for MT-CO1). The asterisks represent the significance levels calculated by two-way ANOVA with Sidak's multiple comparisons test: *P = 0.0262 (S2 vs S6), *P = 0.0440 (control vs S6).
B Relative mRNA expression of one COX (CIV) subunit (MT-CO1) and one CI subunit (ND1) normalized to the expression of GAPDH in skeletal muscle of twelve control animals (wild-type and heterozygous) and six knock-out animals at three months of age. Data are presented as mean ± SEM. (n = 12 for controls, n = 6 for KO).

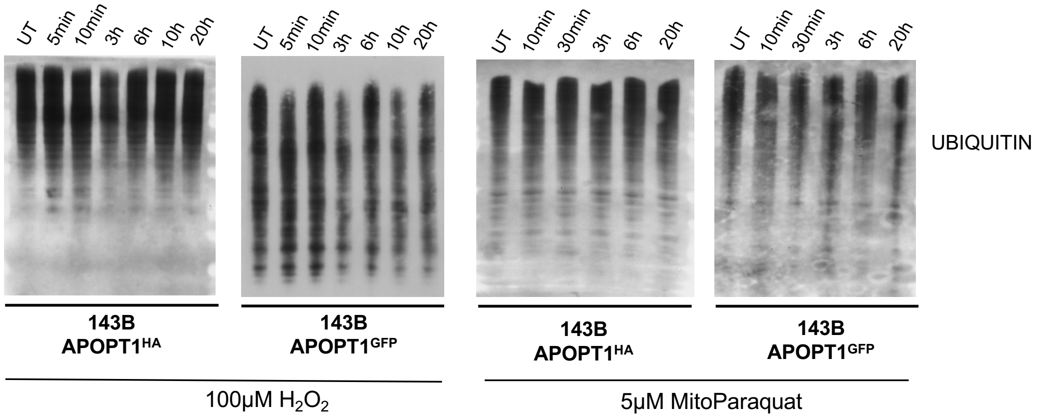

Appendix Figure S8 – Ubiquitination analysis of cells stressed with oxidants
Western blot analysis of SDS-PAGE of total lysates from 143B cells transduced with APOPT1^{HA} or APOPT1^{GFP} treated with H_2O_2 and MitoPQ. No accumulation of ubiquitinated proteins was observed with any of the treatments.
