# Supplementary material for: APOPT1/COA8 assists COX assembly and is oppositely regulated by UPS and ROS
Source: EMBO Mol Med. 2018 Dec 14;11(1):e9582. doi: 10.15252/emmm.201809582 (PMC6328941; doi:10.15252/emmm.201809582)
Supplement: Supplementary file 3 — Source Data for Figure 3 [file EMMM-11-e9582-s002.pdf]

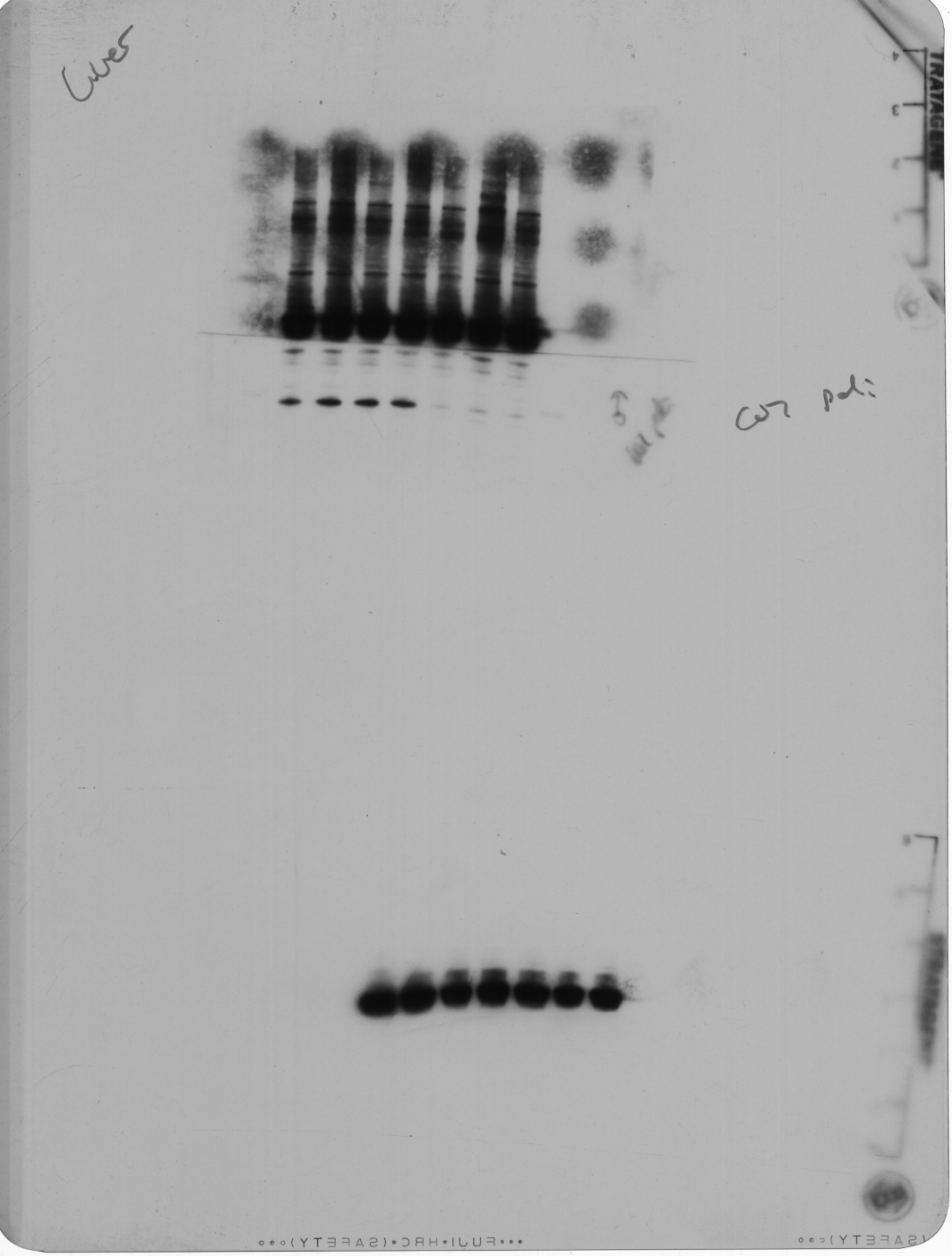

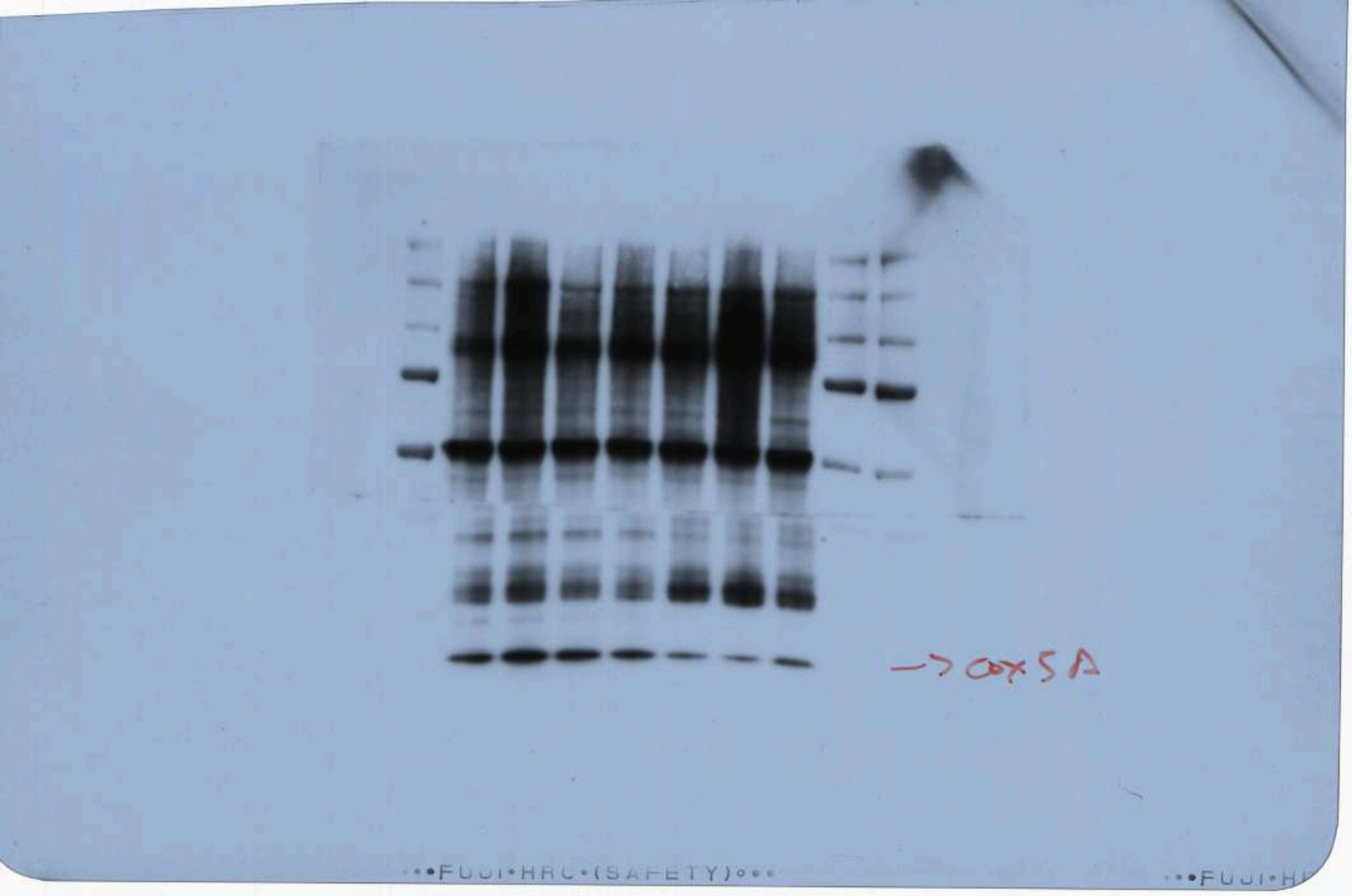
→ COX 5A
•••FUJI•HRC•(SAFETY)•••
•••FUJI•H

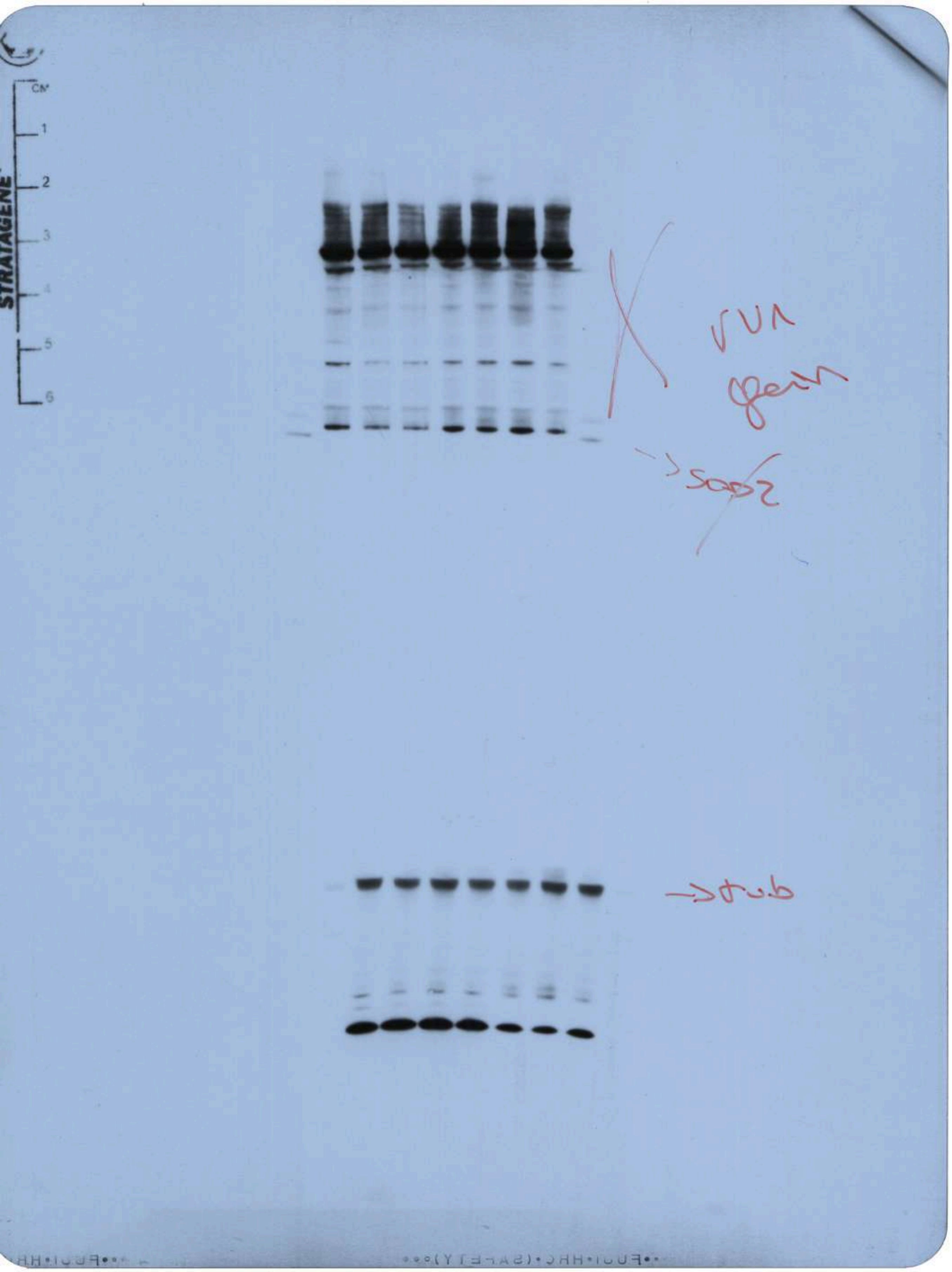
STRATAGENE
CM
1
2
3
4
5
6
tub

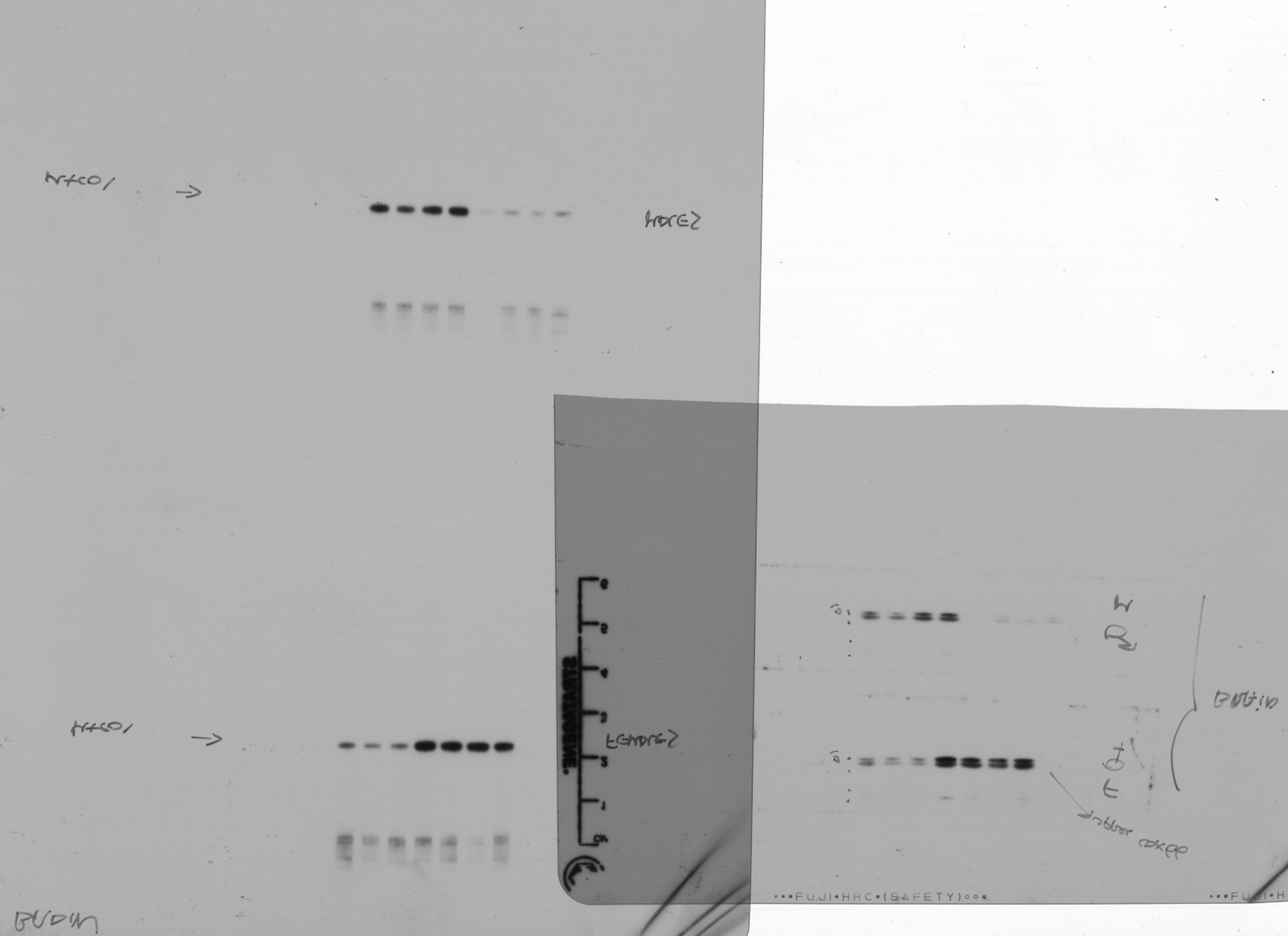
•••FUJI•HRC•(SAFETY)•••

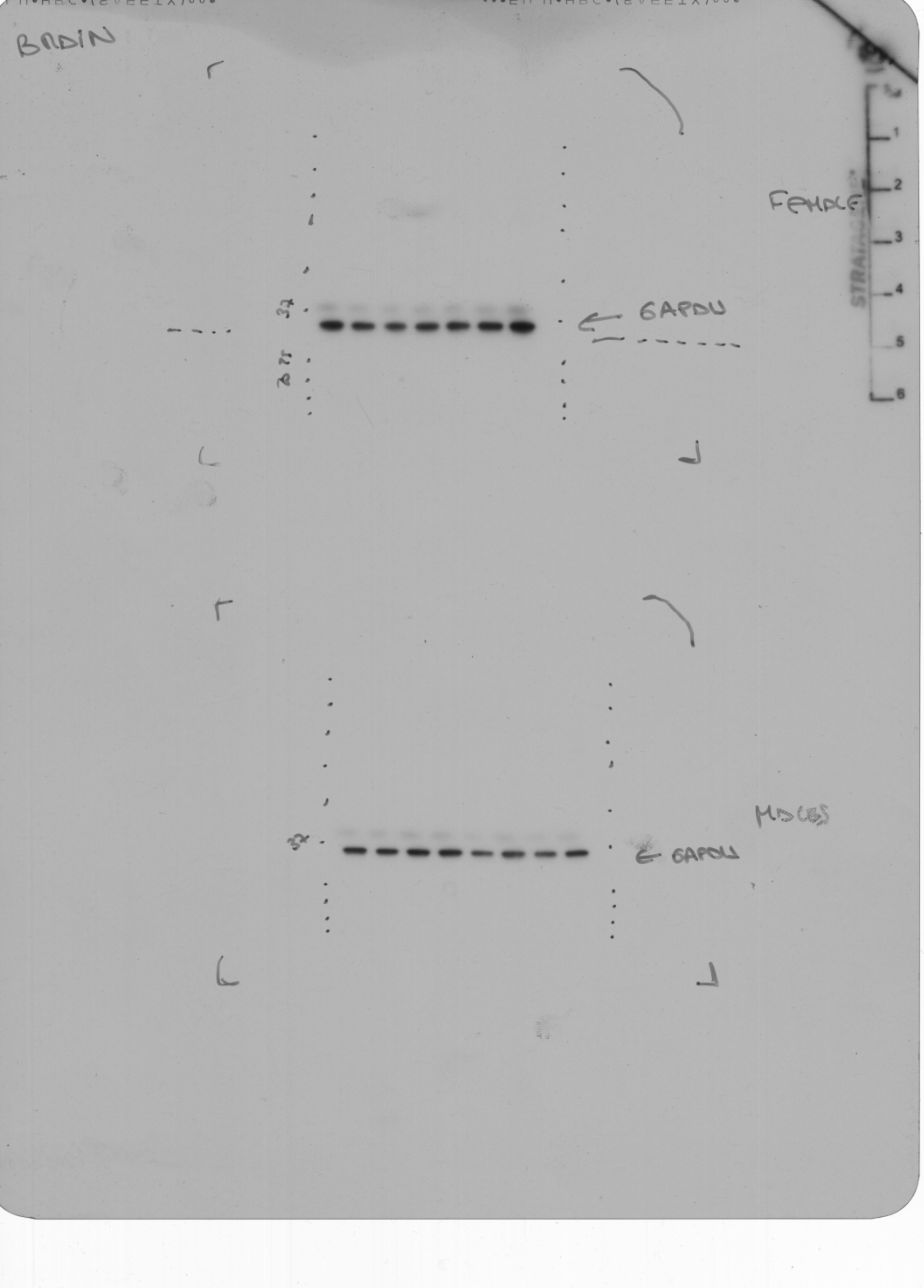
BRAIN
FEMALE
37
GAPDH
MALES
GAPDH

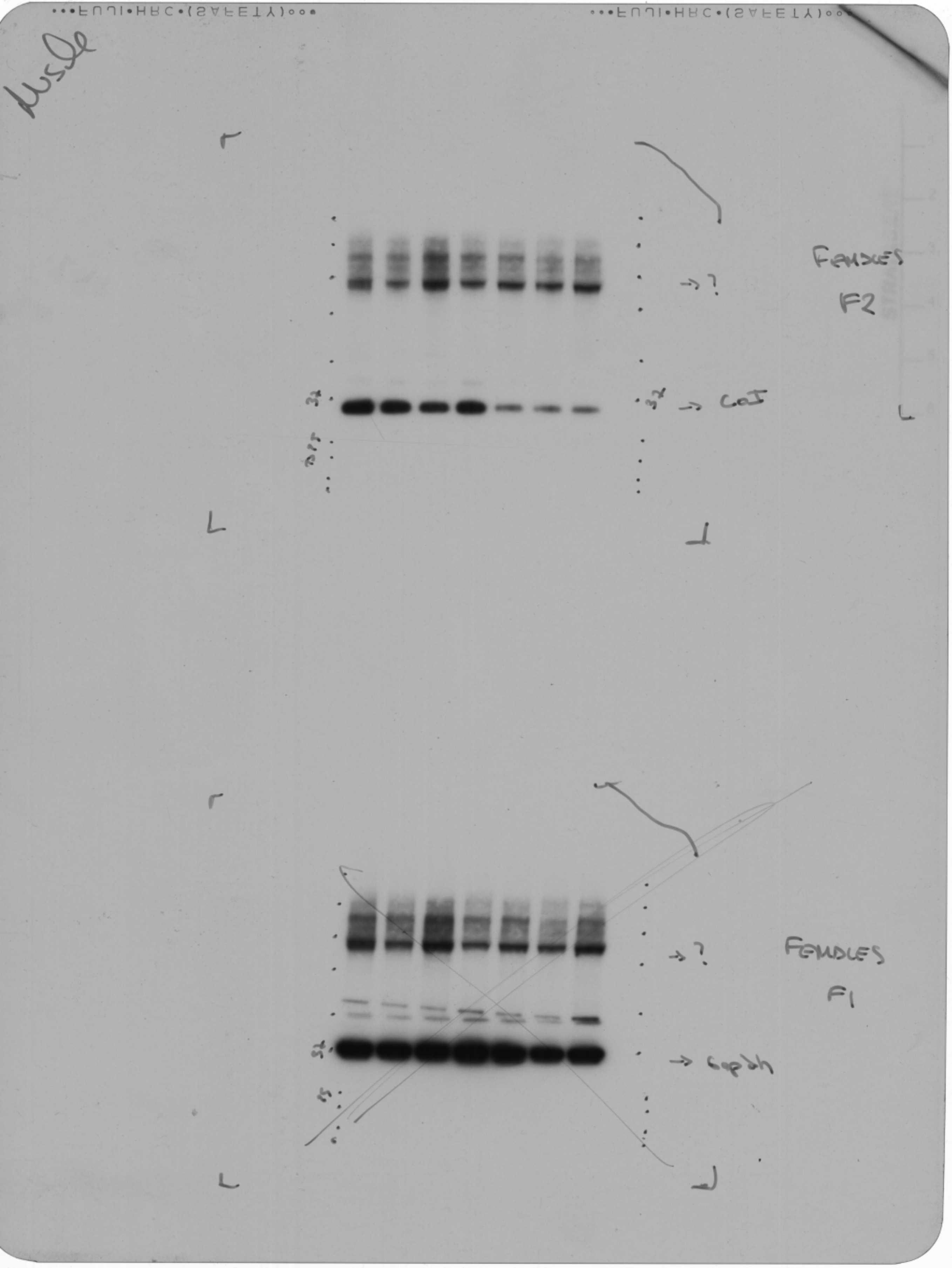

FEMALES
F2
→ ?
FEMALES
F1
→ ?
→ Gapdh

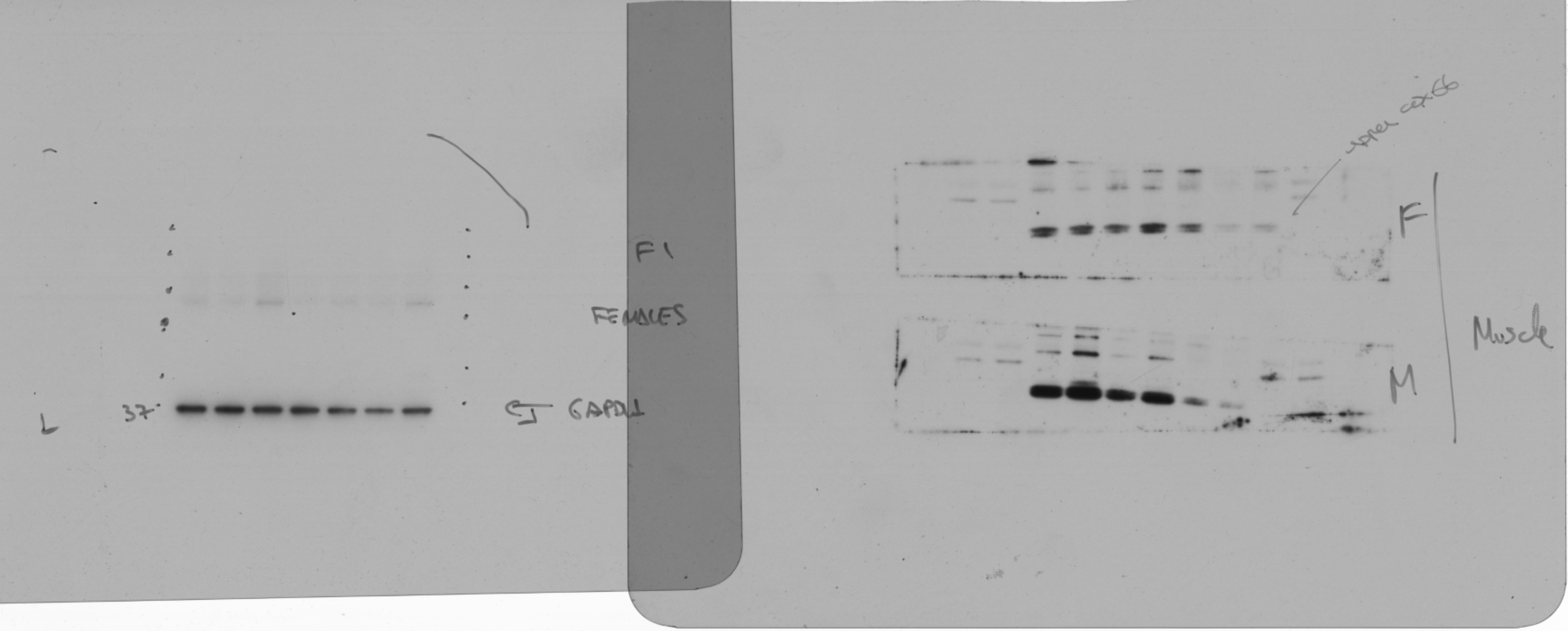
F1
FEMALES
37
GAPDH
F
M
Muscle

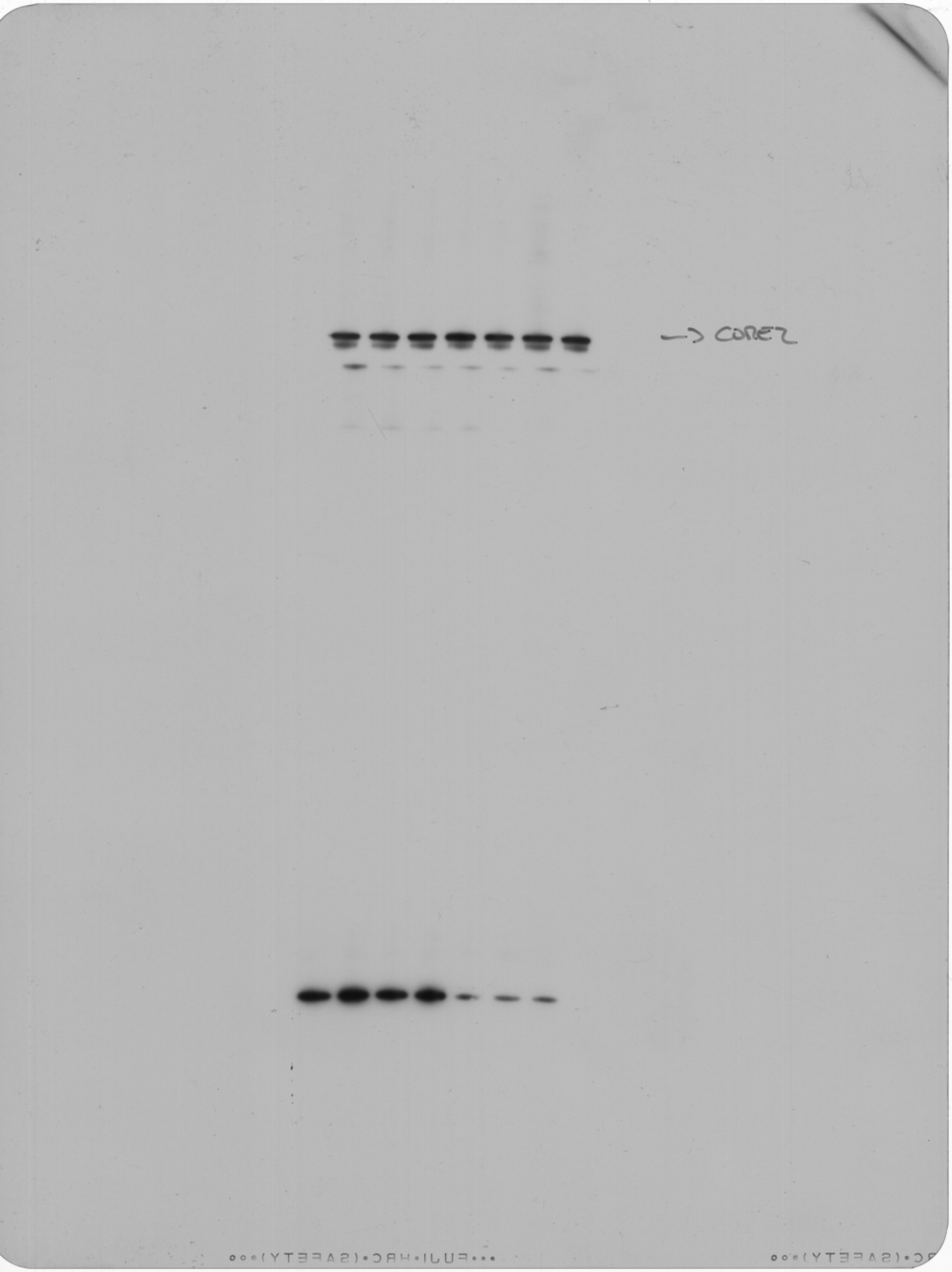
CORE2

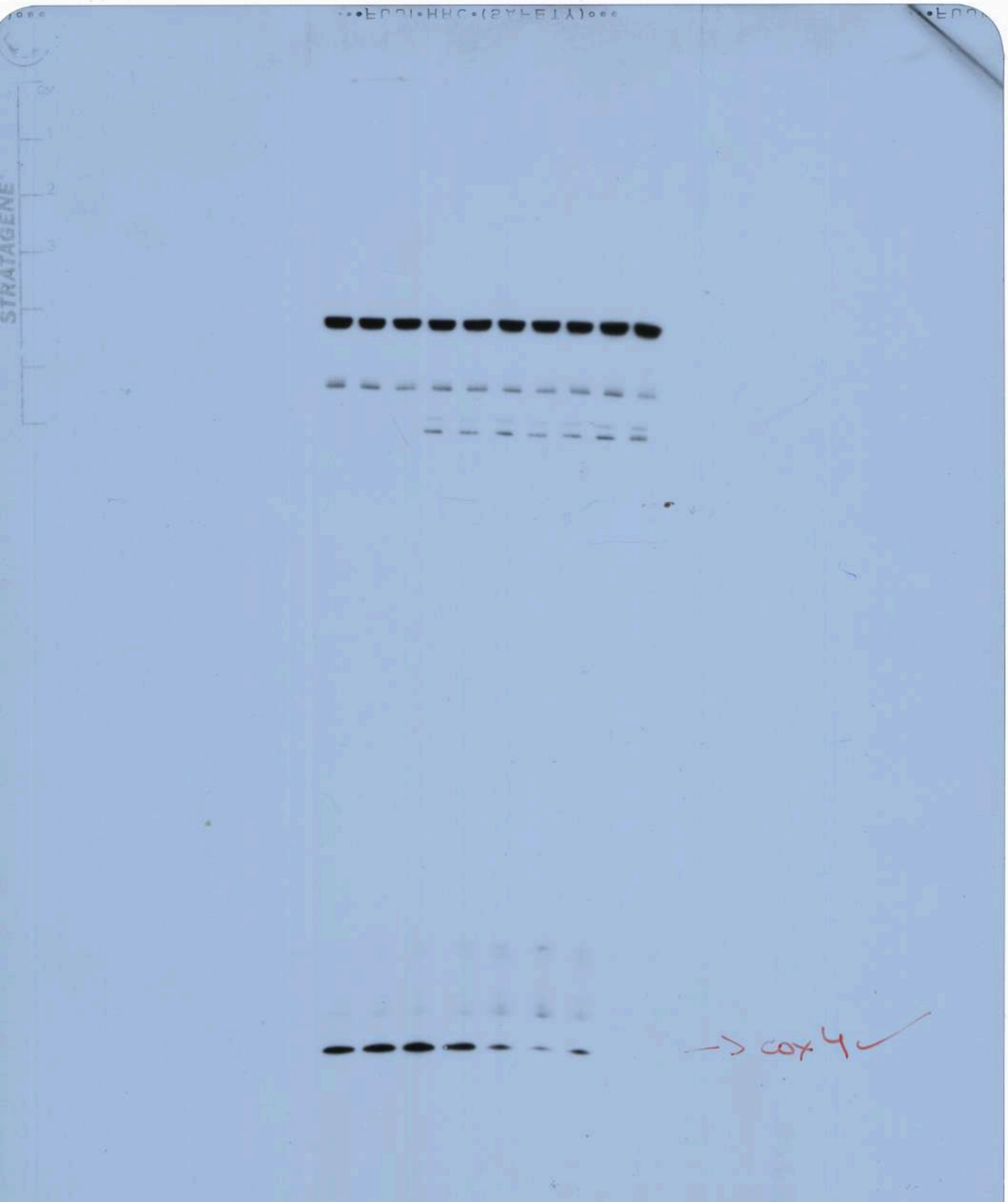
STRATAGENE
cox 4

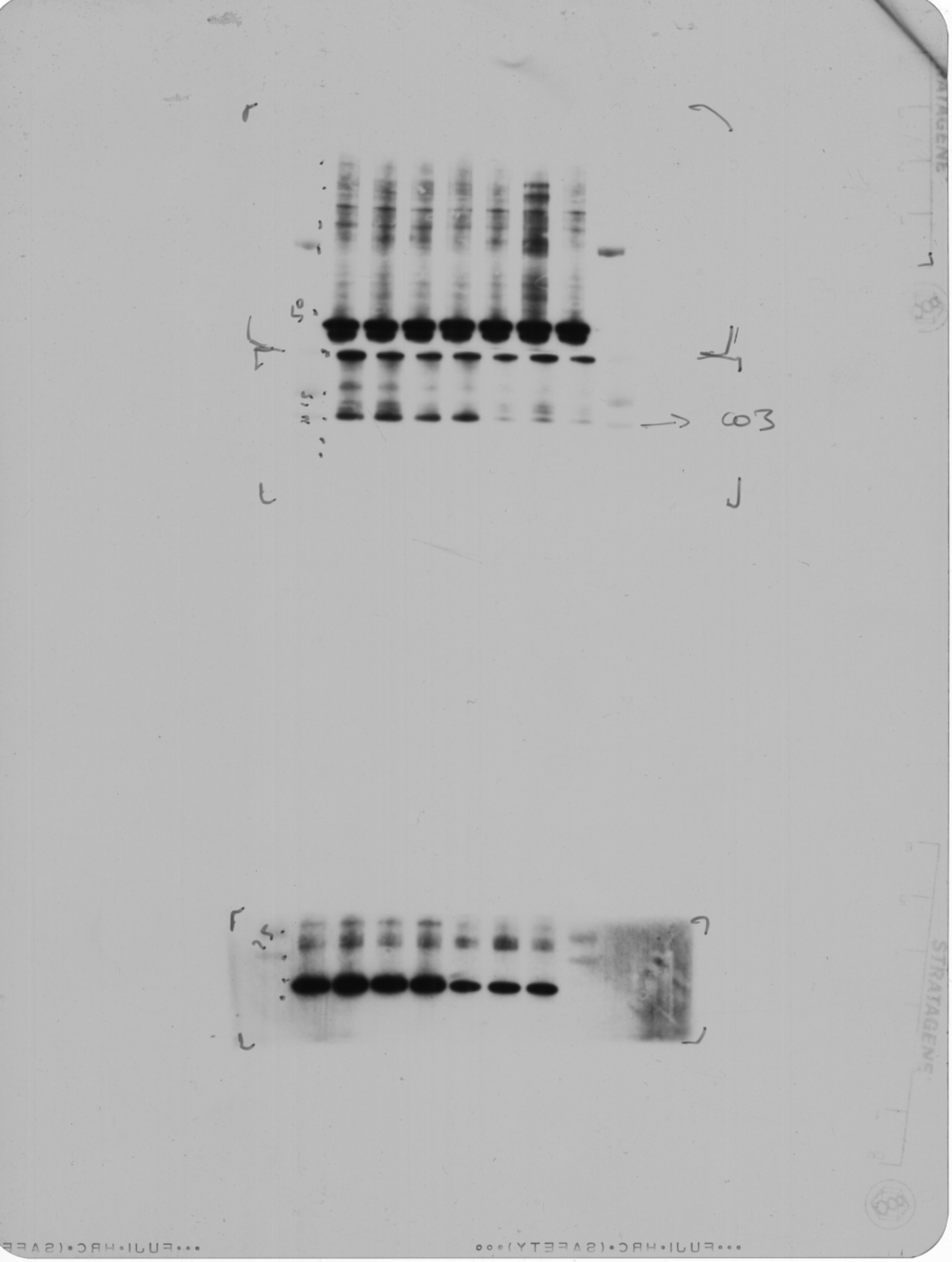
50
co3
25

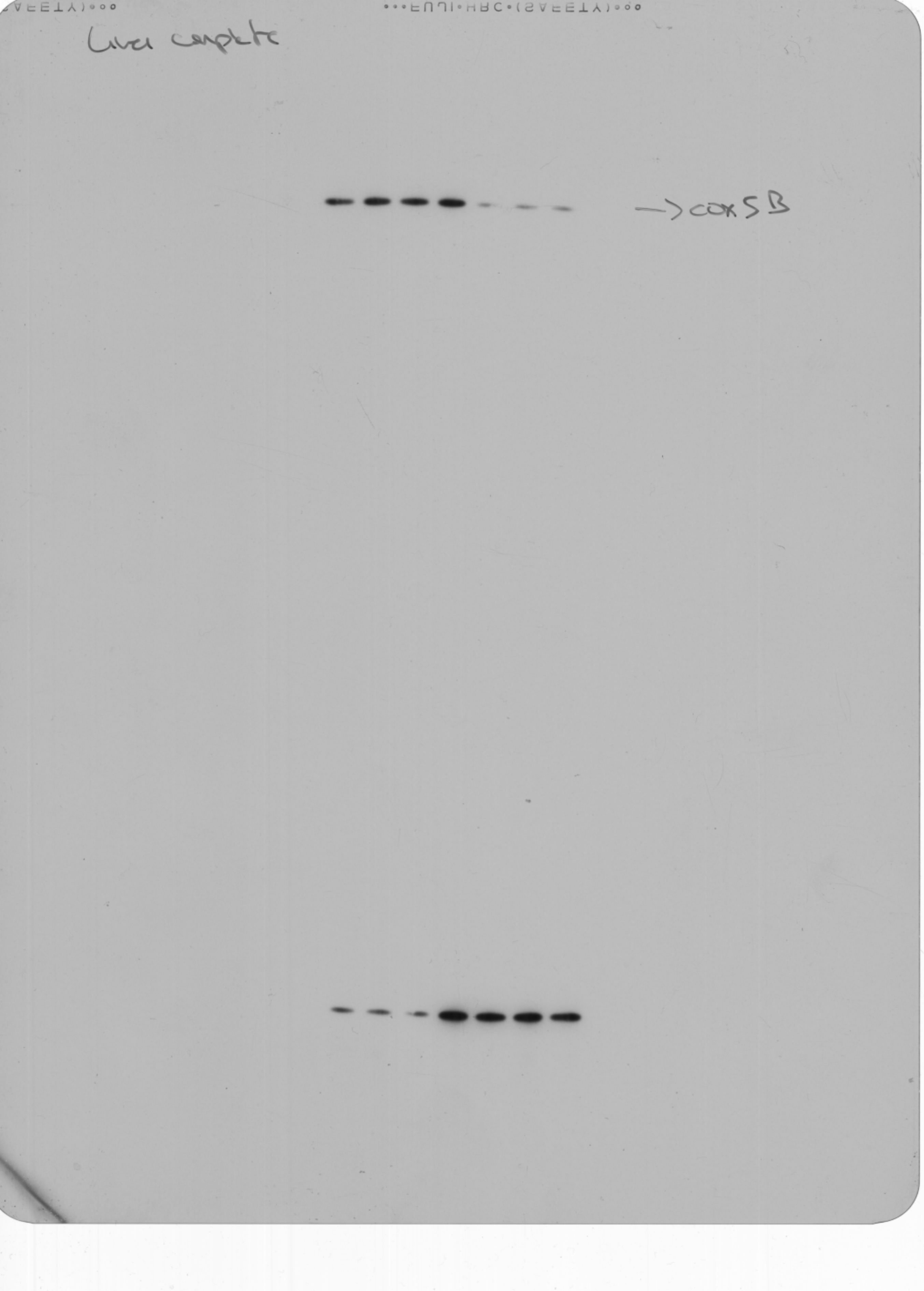
Liver complete
cox 5B

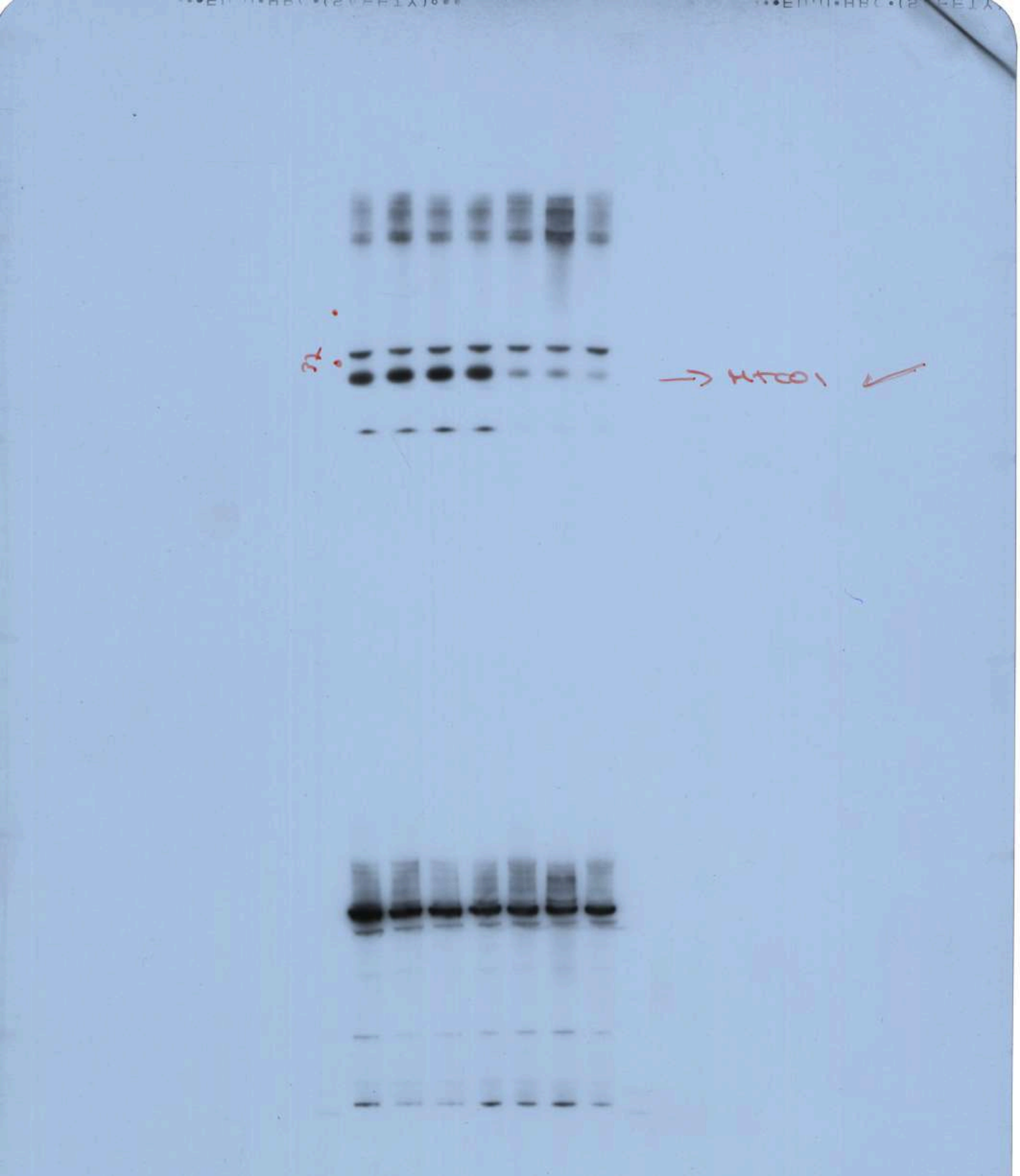
32
HTCO1

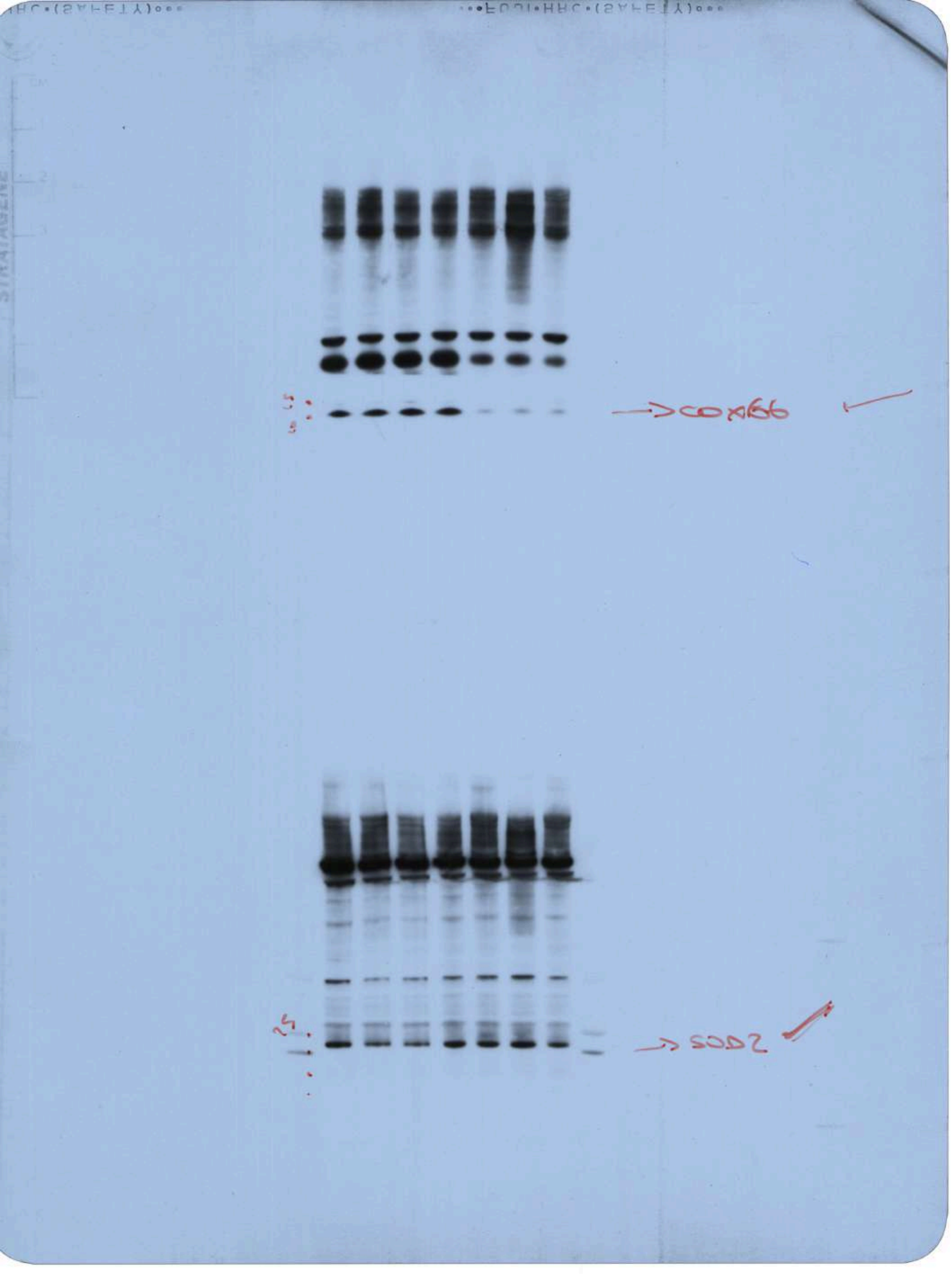
COX6b
25
SOD2

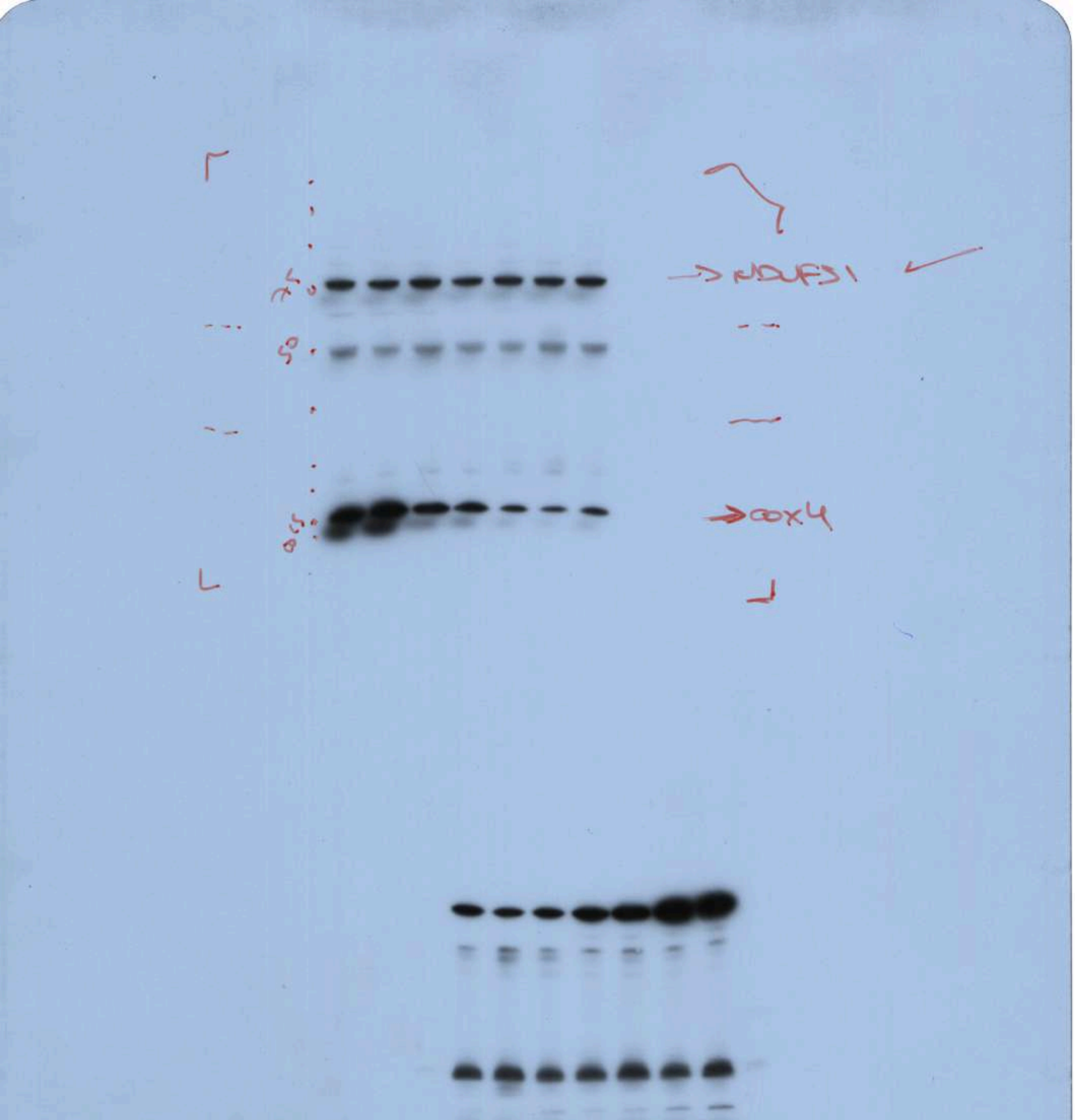
NDUFS1
COX4
