# Supplementary figures and images for: APOPT1/COA8 assists COX assembly and is oppositely regulated by UPS and ROS
Source: EMBO Mol Med. 2018 Dec 14;11(1):e9582. doi: 10.15252/emmm.201809582 (PMC6328941; doi:10.15252/emmm.201809582)

100  
90  
80  
70  
60  
50  
40  
30  
20  
10  
0

100

100

100 100 100 100 100

→ 100 lac  
( $ce_3^-$ )

→ 3502

→ 3505

→ 3506

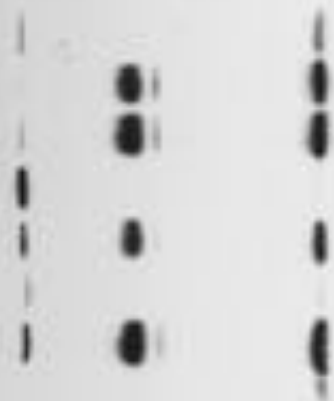

→ 3507

→ 3508

→ 3509

→ 3510

→ 3511

✓

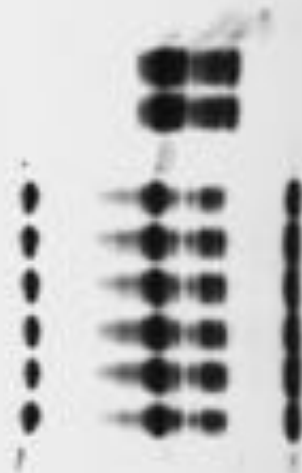

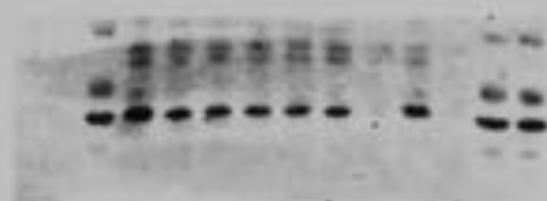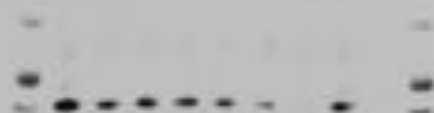

Pro  
CM

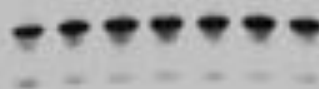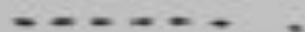

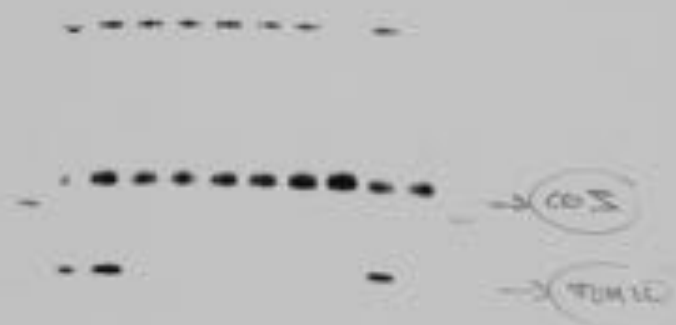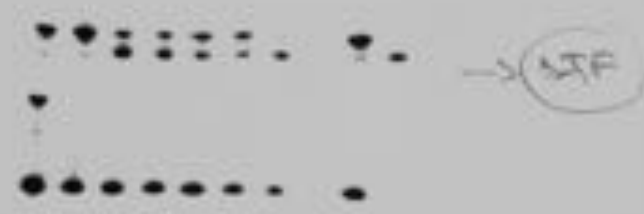

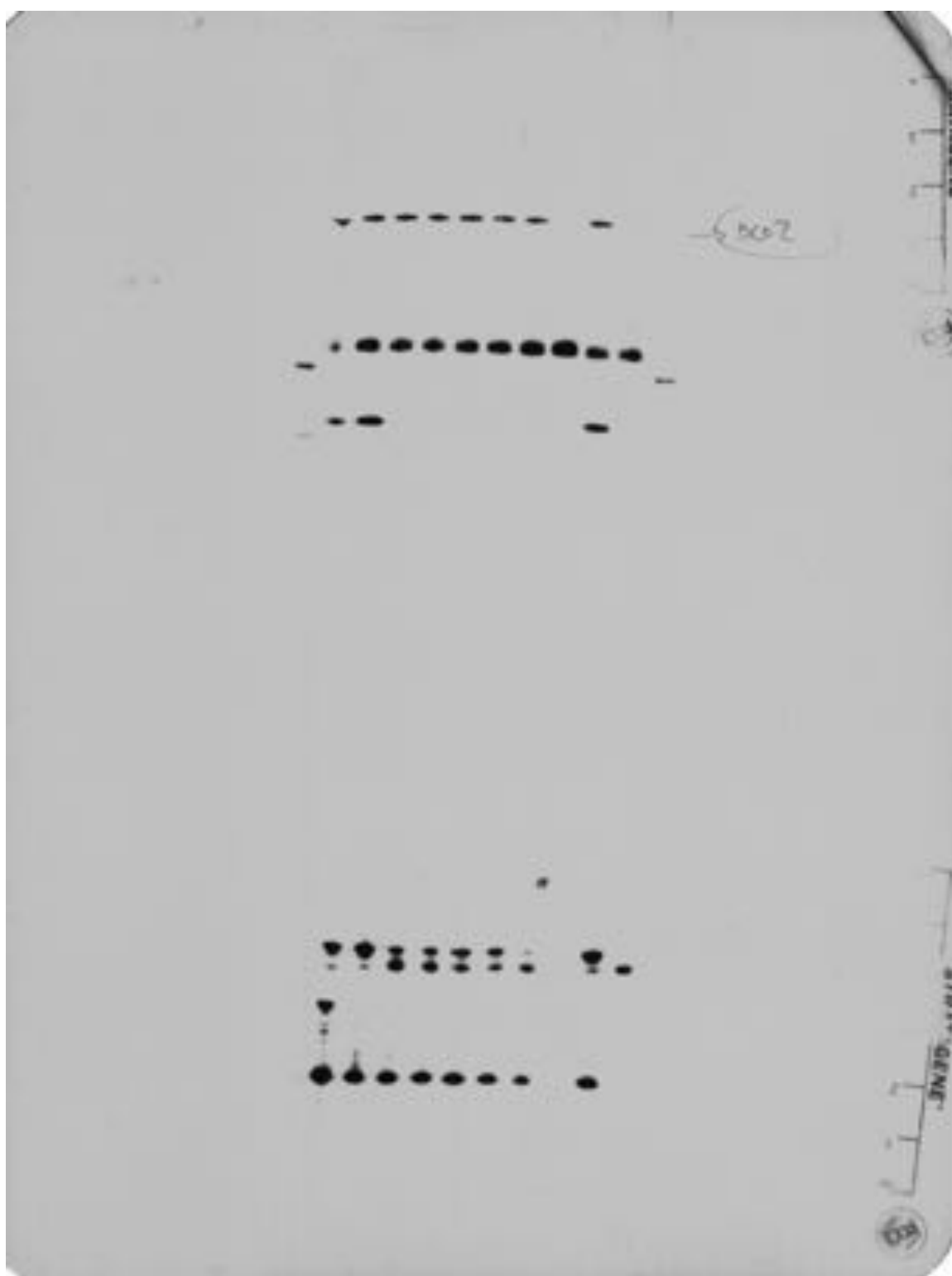

1 2 3 4 5 6 7 8 9 10

1 2 3 4 5 6 7 8 9 10

1 2 3 4 5 6 7 8 9 10

1 2 3 4 5 6 7 8 9 10

1

1 2 3 4 5 6 7 8 9 10

→ KIT

→ DIL2

3100-GENE

8

1 2 3 4 5 6 7 8 9

10 11 12 13 14 15 16 17 18 19

20 21 22 23 24 25 26 27 28 29

30 31 32 33 34 35 36 37 38 39

dry pen + day

→ cost

1 2 3 4 5 6 7 8 9

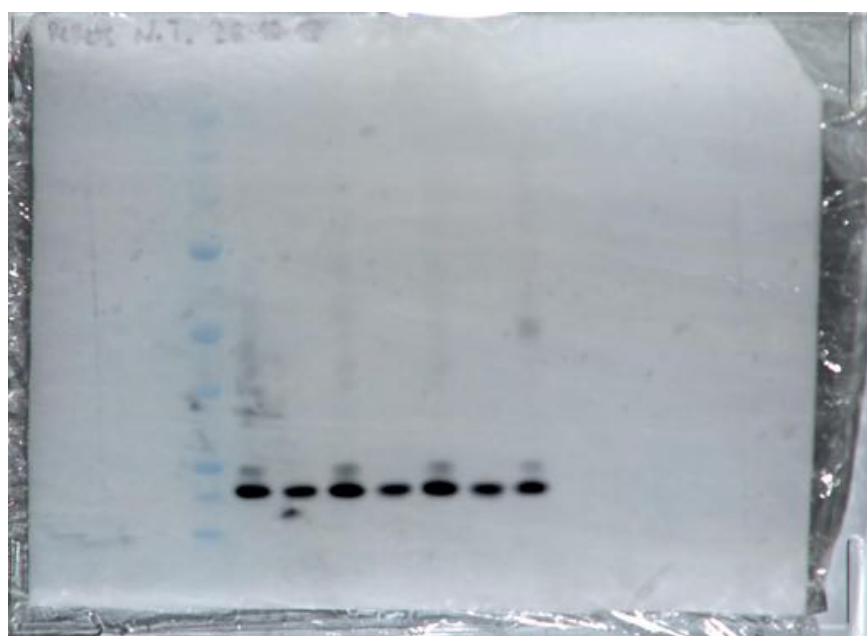

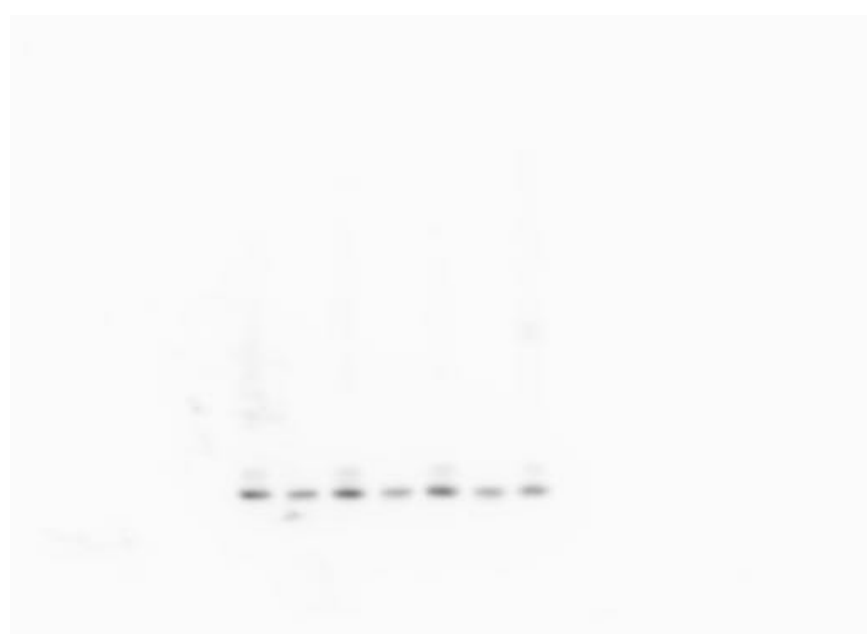

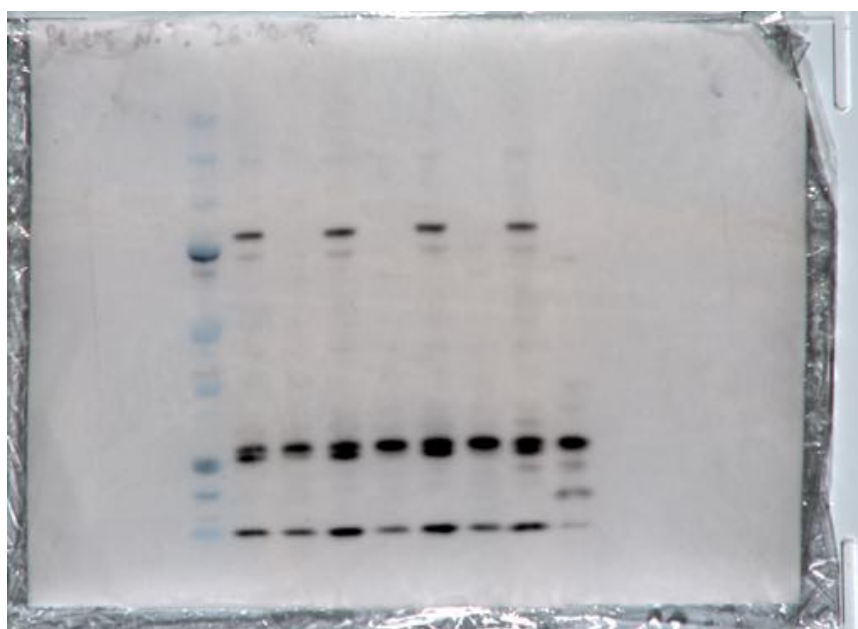

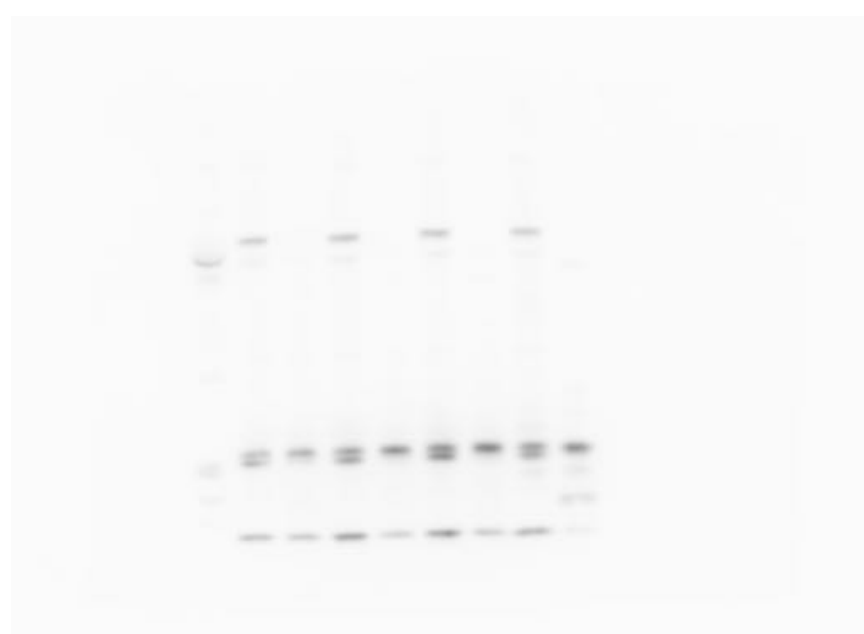

94242 N. T. 26-10-18

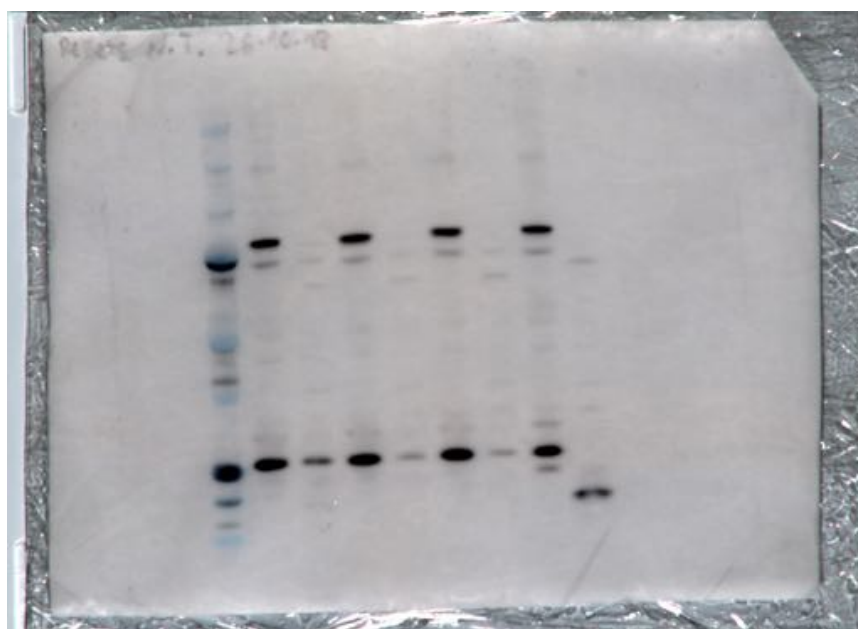

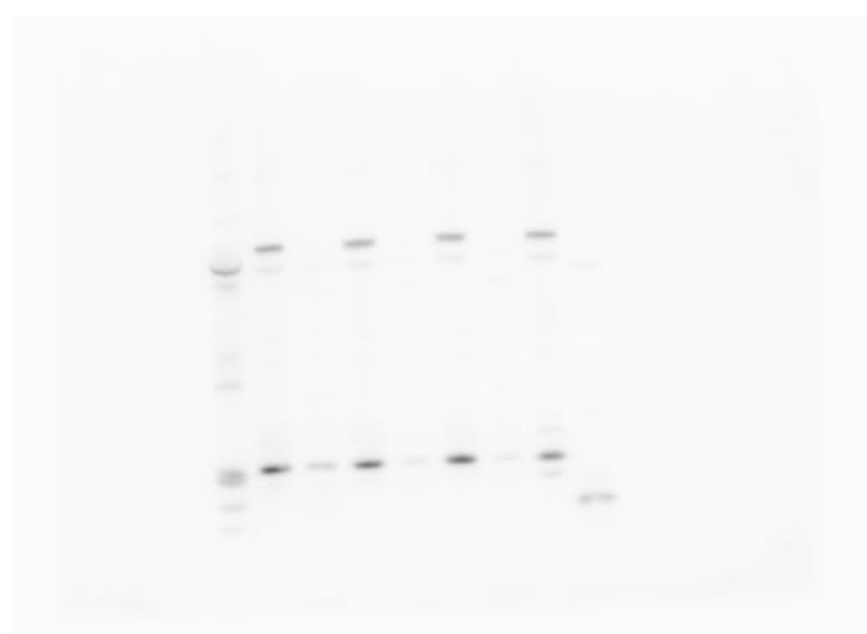

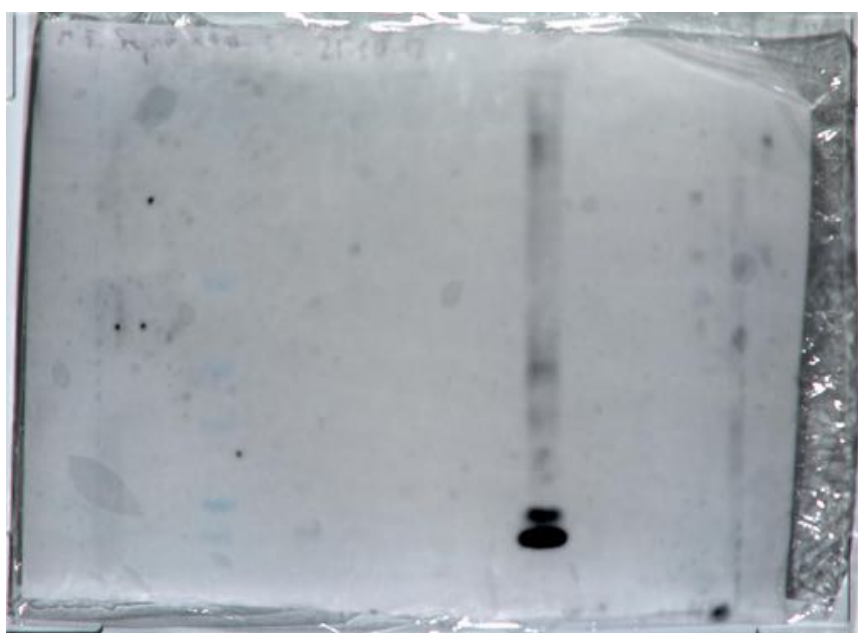

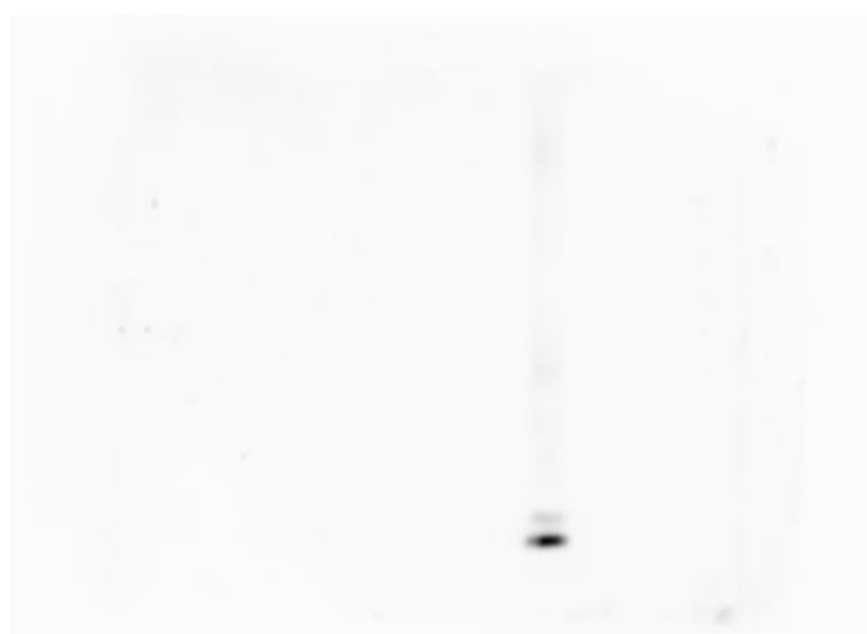

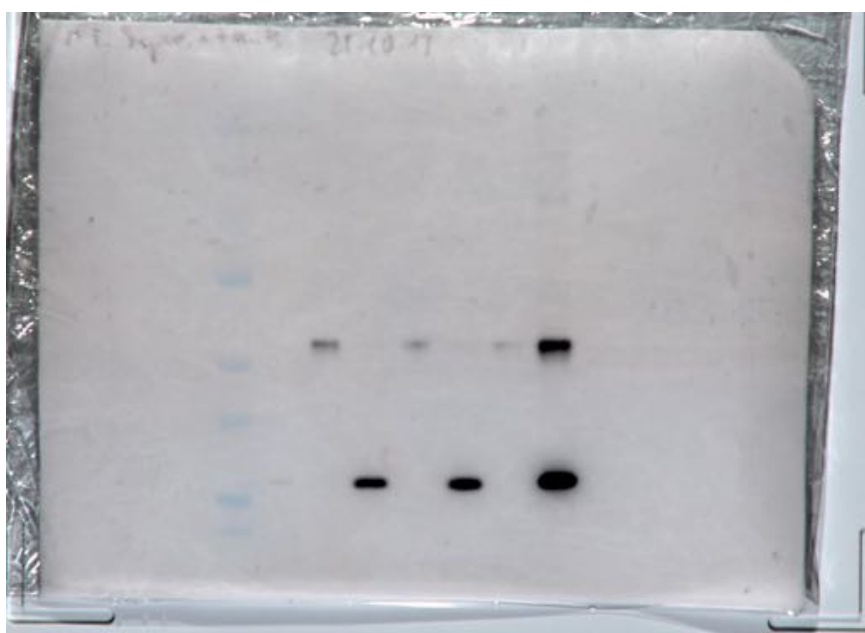

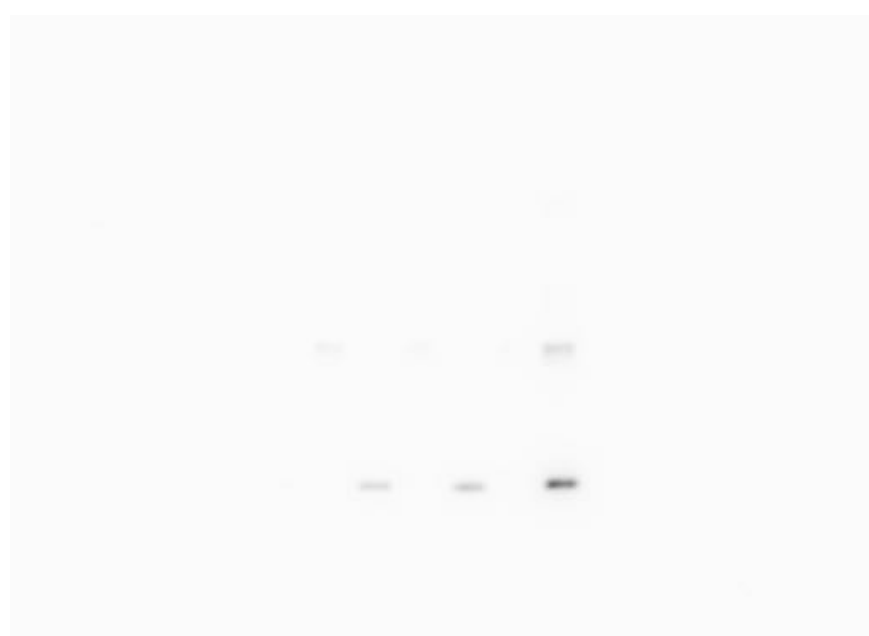

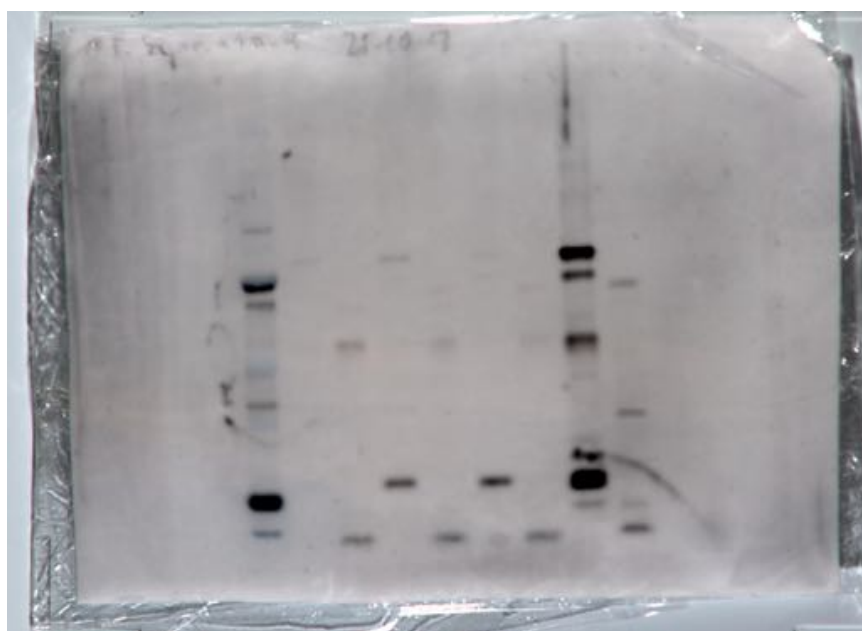

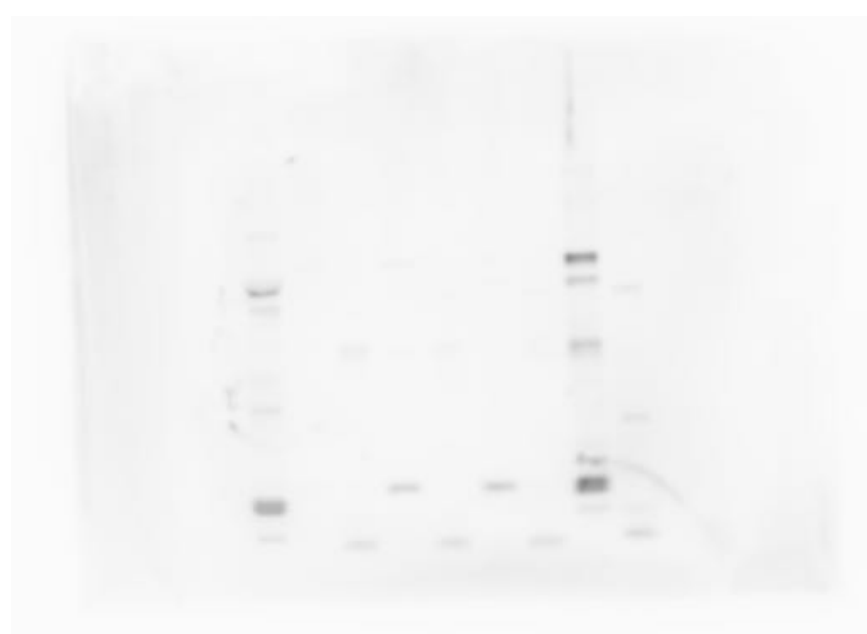

Supplement: Supplementary file 4 — Source Data for Figure 4 [file EMMM-11-e9582-s003.pdf]

Pa. Am.

2 001-1 001-2 001-3 001-4 C47F5

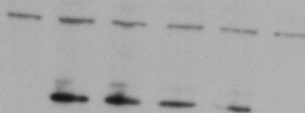

← HA

Doti-HA on 4°C  
ab<sup>2</sup> anti-net 1:1000 1h RT

C.A.

2 001-1 001-2 001-3 001-4 C47F5

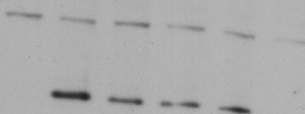

← HA

STRATAGENE

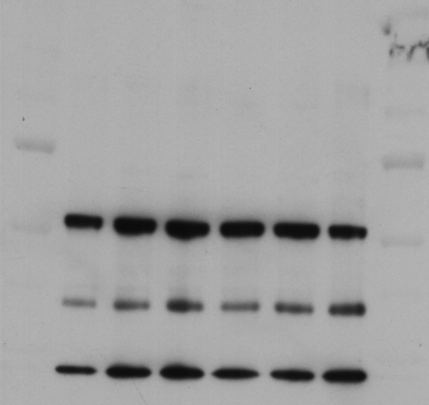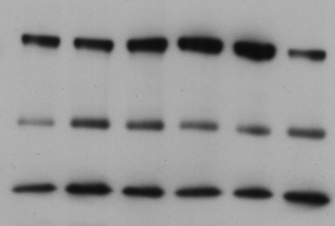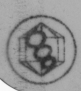

Supplement: Supplementary file 5 — Source Data for Figure 5 [file EMMM-11-e9582-s004.pdf]

4-42  $\underbrace{F_{0.500, 1, 2000}}_{1.000000000}$

Further:

3

1954

275

$\frac{1}{\sqrt{2}}$

 $\text{CO}_2$  100%

PLATE 1

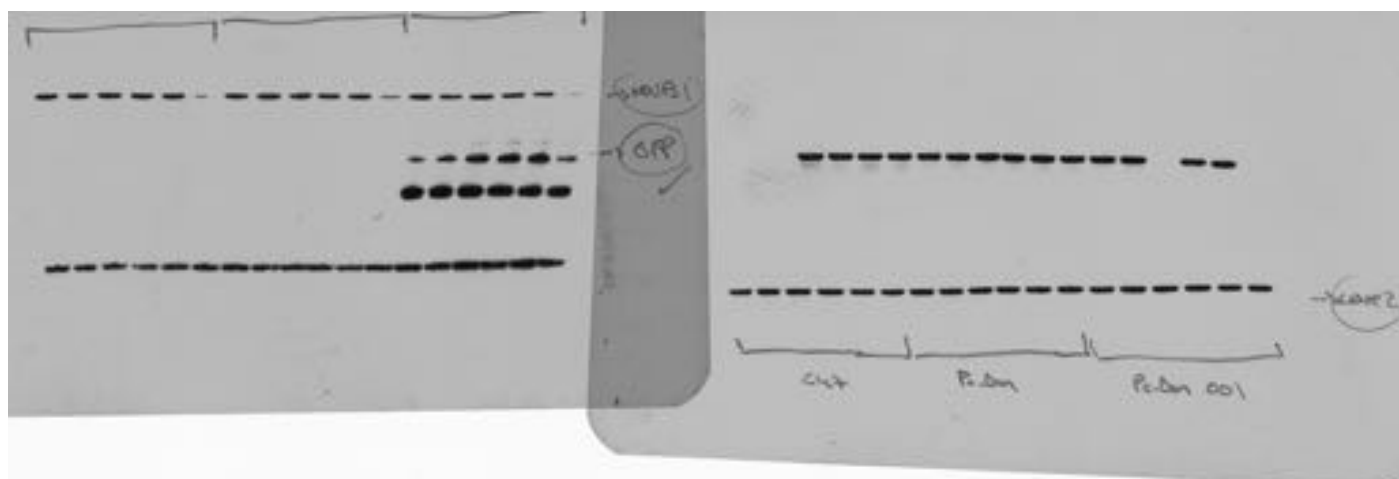

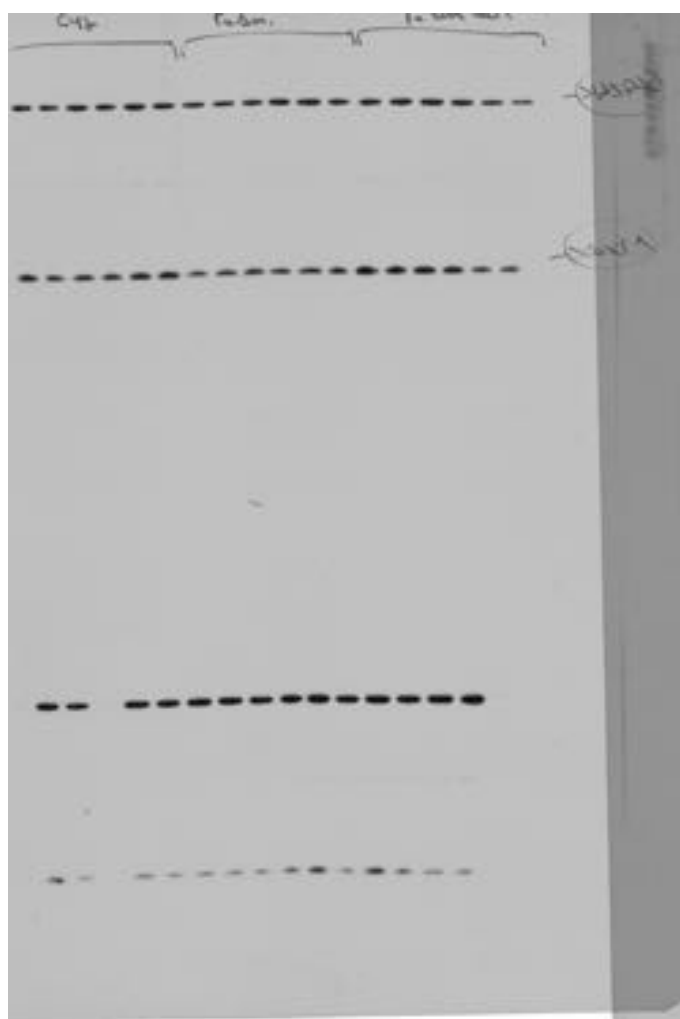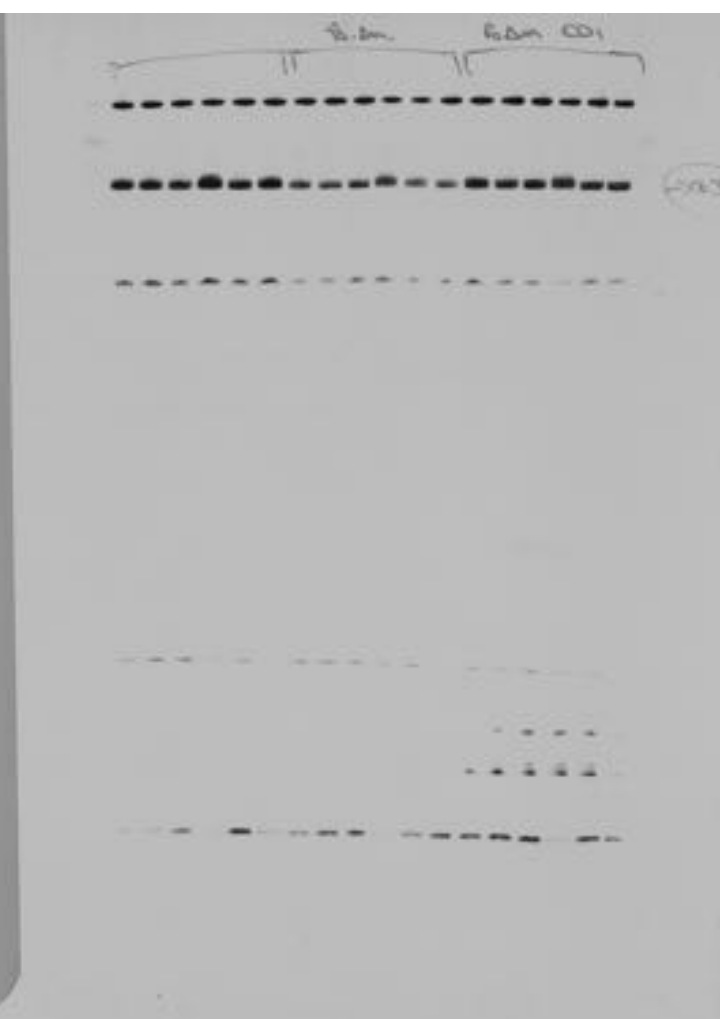

Supplement: Supplementary file 7 — Source Data for Figure 7 [file EMMM-11-e9582-s006.pdf]

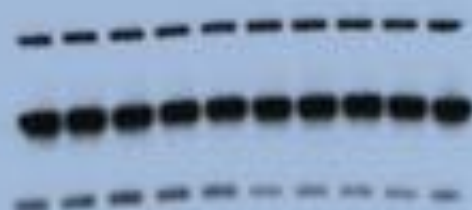

→ 4000 ✓

— 10 —

2025

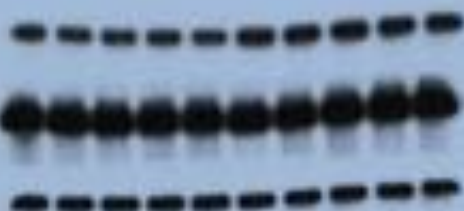

100 100 100 100 100 100 100 100

100 100 100 100 100 100 100 100

100 100 100 100 100 100 100 100

100 100 100 100 100 100 100 100

100 100 100 100 100 100 100 100

100 100 100 100 100 100 100 100

100 100 100 100 100 100 100 100

100 100 100 100 100 100 100 100

100 100 100 100 100 100 100 100

100 100 100 100 100 100 100 100

100 100 100 100 100 100 100 100

100 100 100 100 100 100 100 100

100 100 100 100 100 100 100 100

100 100 100 100 100 100 100 100

→ LEVEL ✓

STRATAGIE

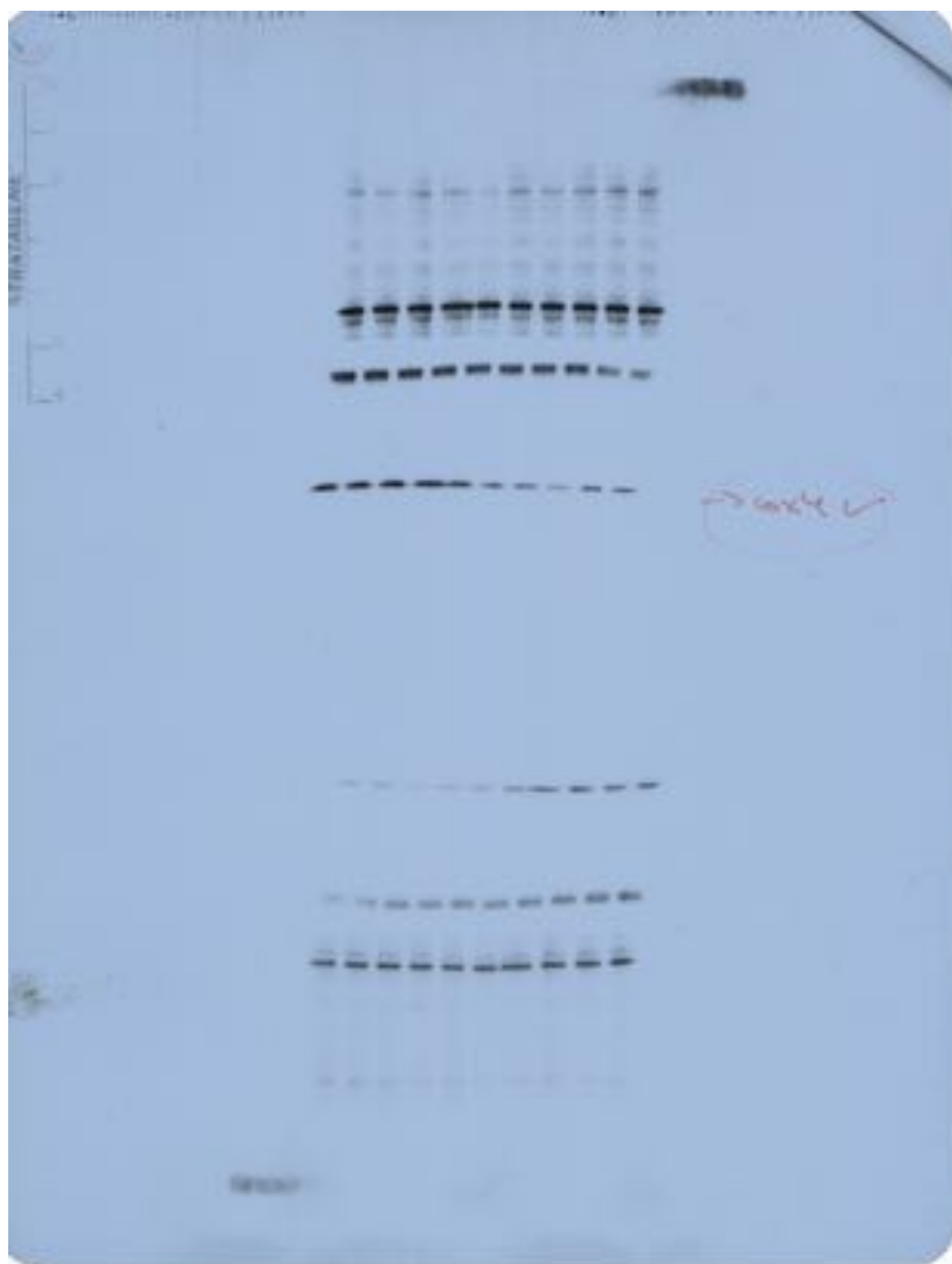

10025 (1)

10025 (1)

10025 (1)

10025 (1)

10025 (1)

10025 (1)

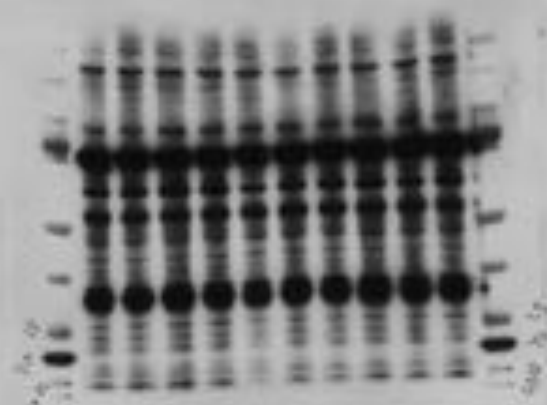

AP0PT1  
only.

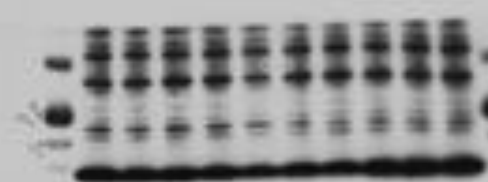

→ cor. sol:

STRATIGINE

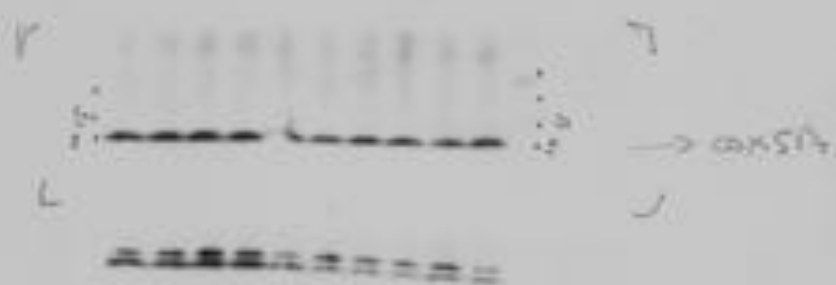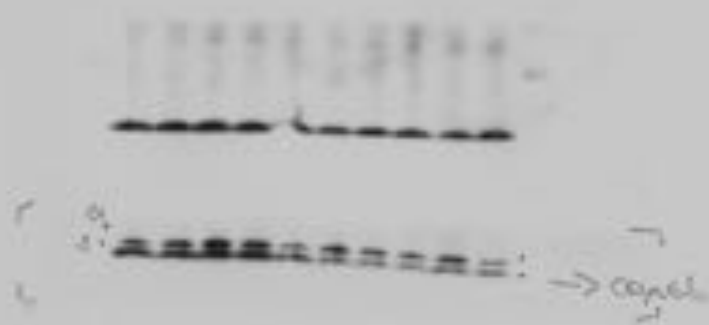

bio 10 - mofa pz

1

100

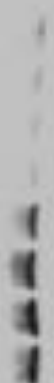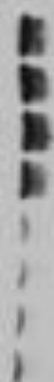

(10) 100

bio 10 - mofa pz

100

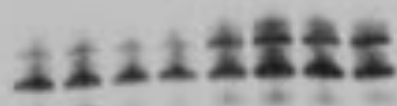

Supplement: Supplementary file 8 — Source Data for Appendix [file EMMM-11-e9582-s007.zip › Appendix_Figure__S2.pdf]

52  
m

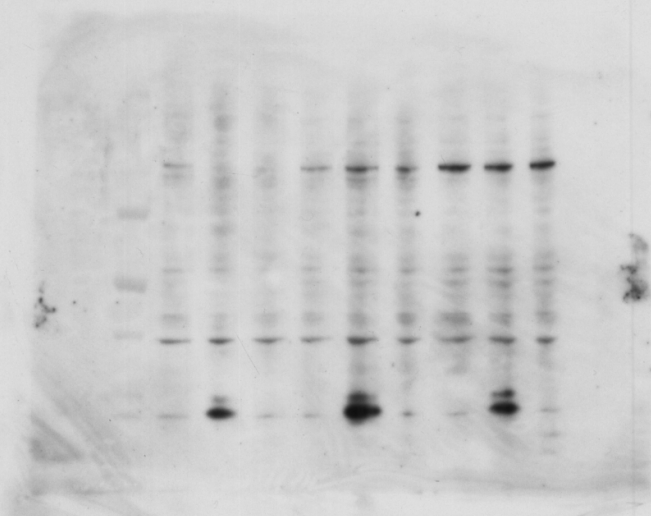

Supplement: Supplementary file 8 — Source Data for Appendix [file EMMM-11-e9582-s007.zip › Appendix_Figure_S3.pdf]

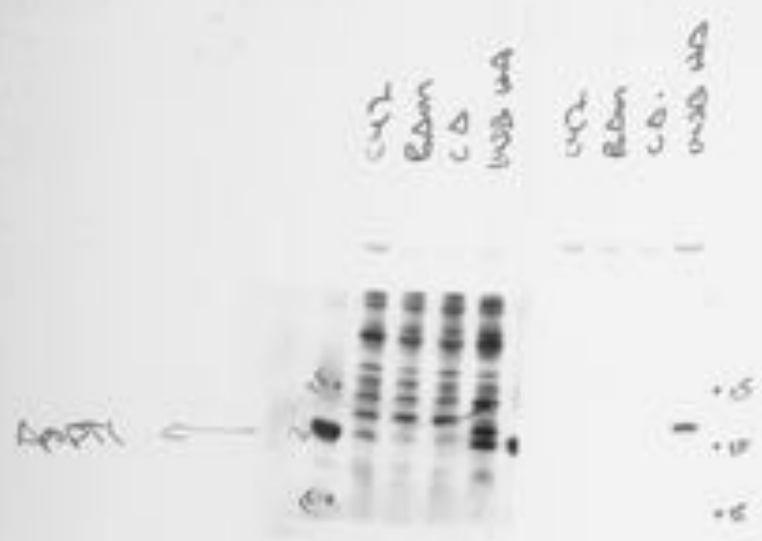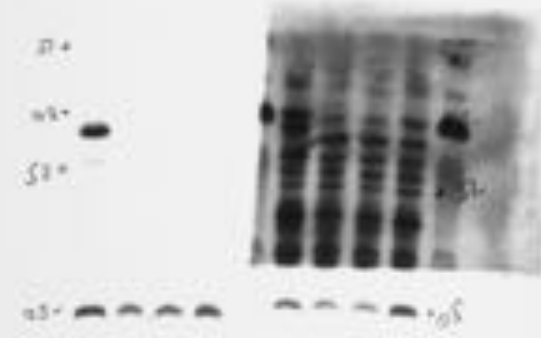

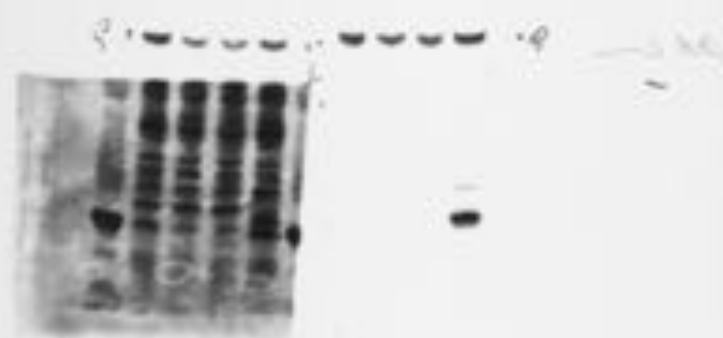



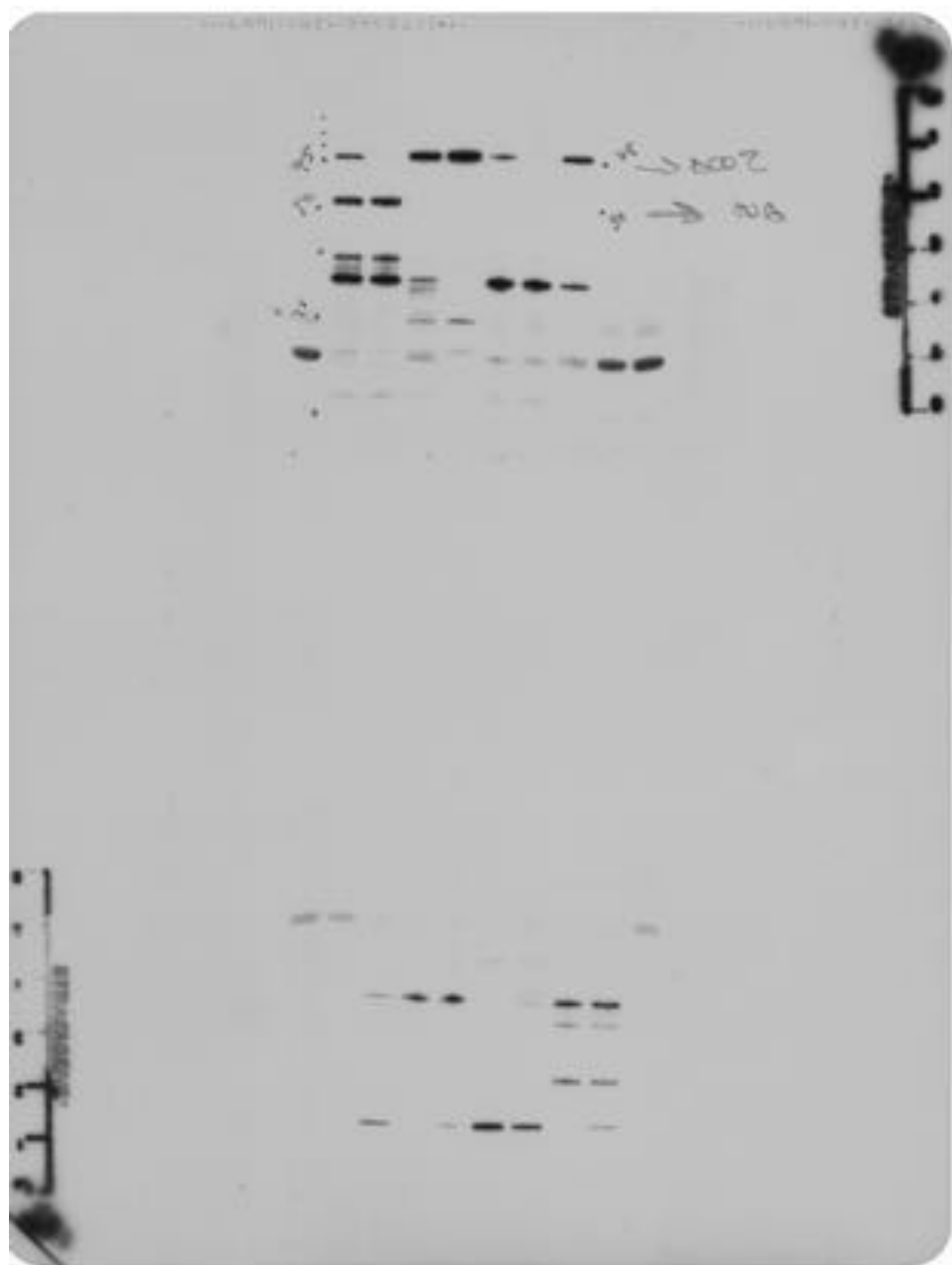

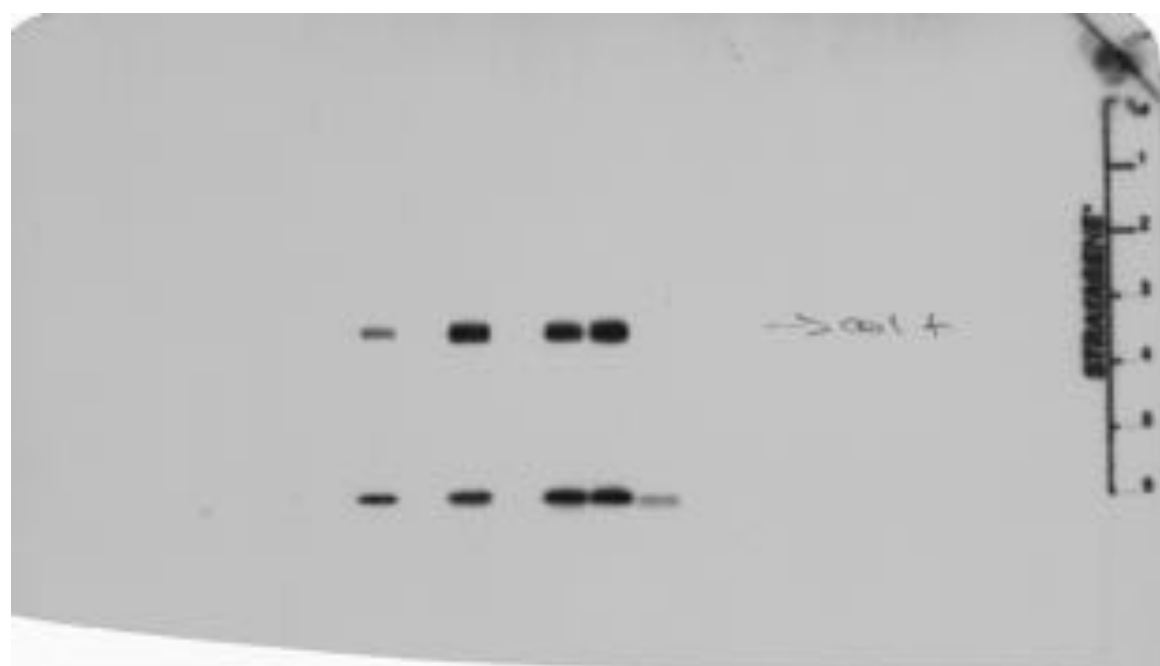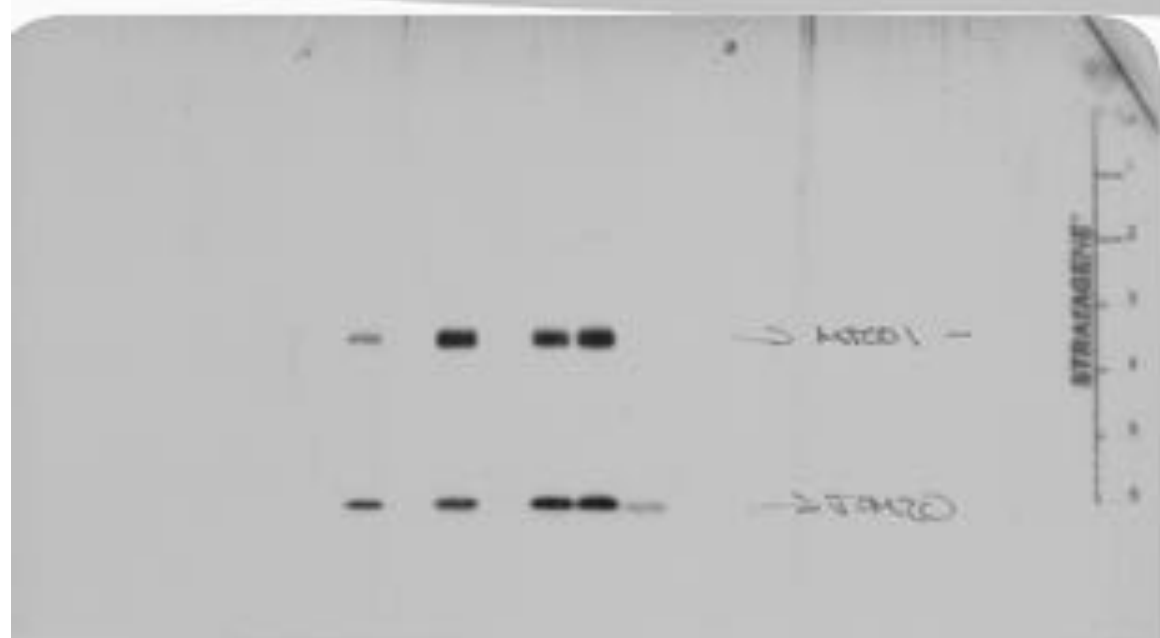

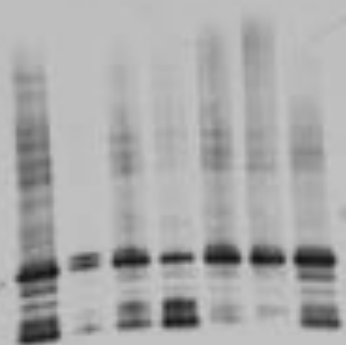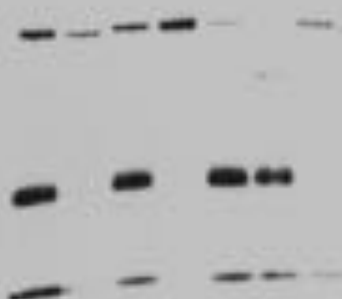

\*\*\*XTOTAB(2R)ALD\*\*\*

\*\*\*XTOTAB\*\*\*

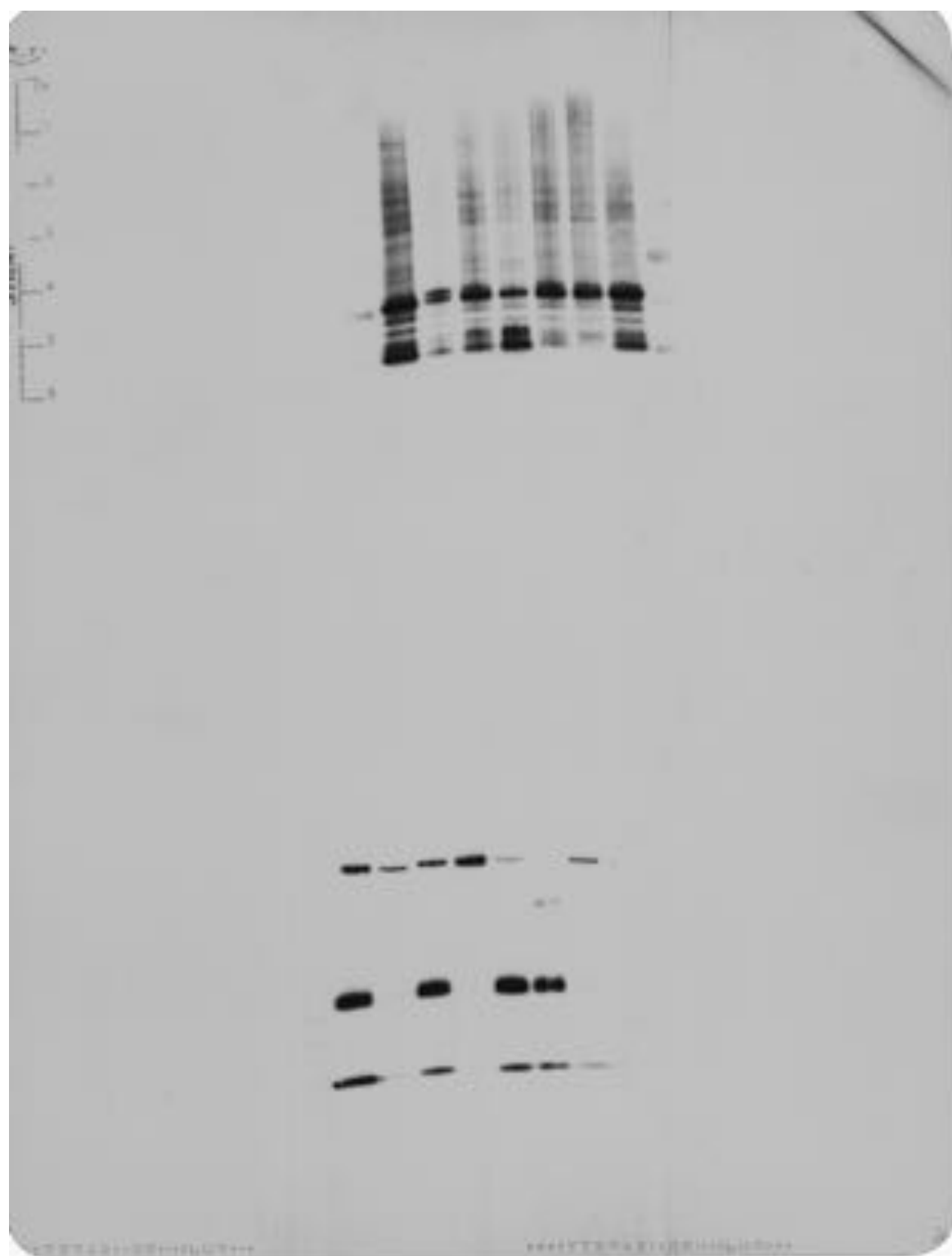

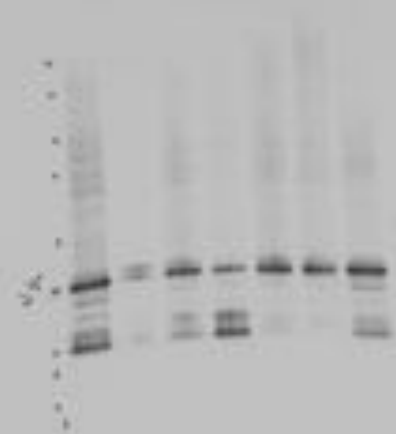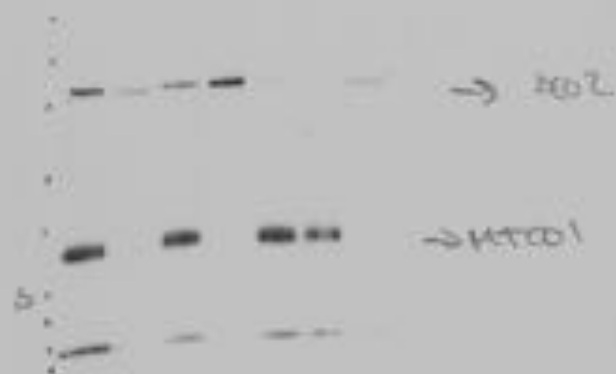

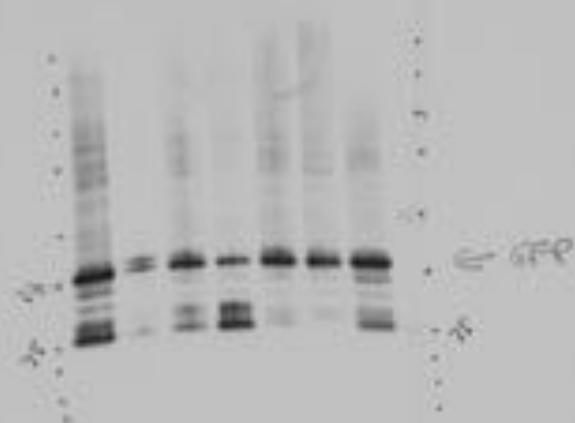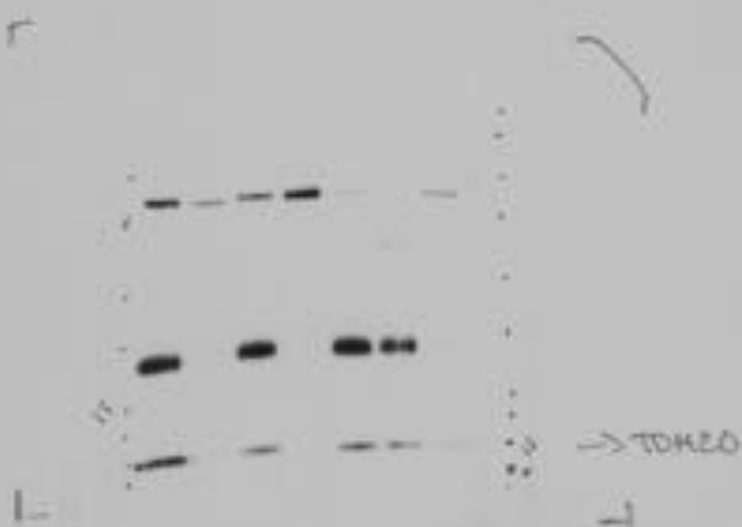

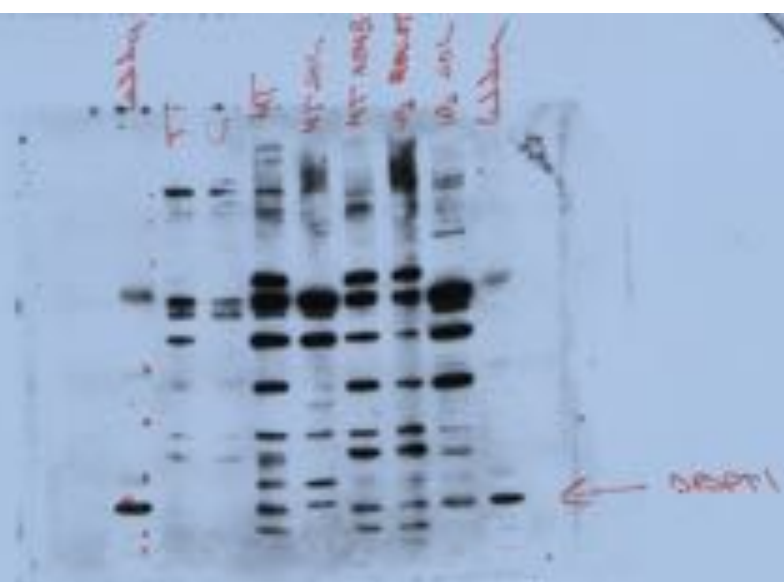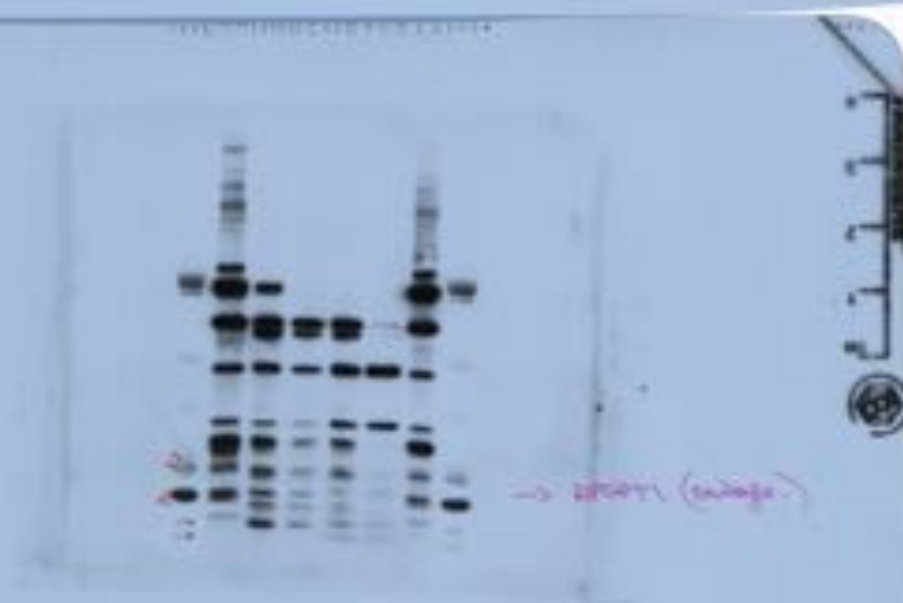

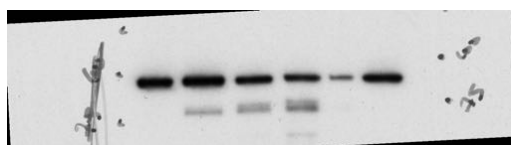

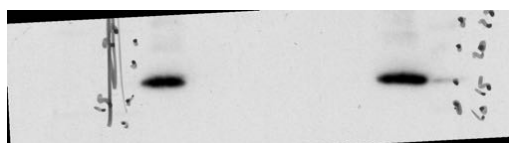

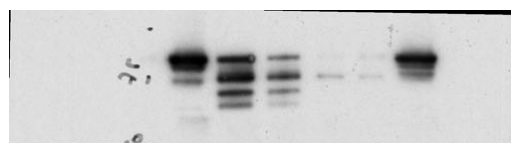

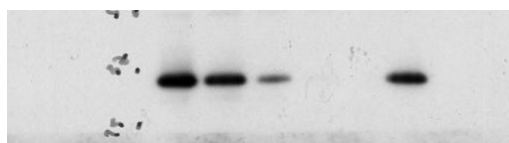

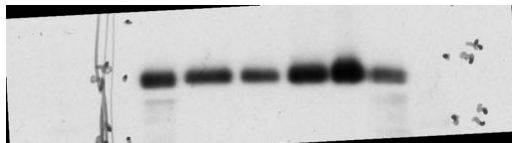

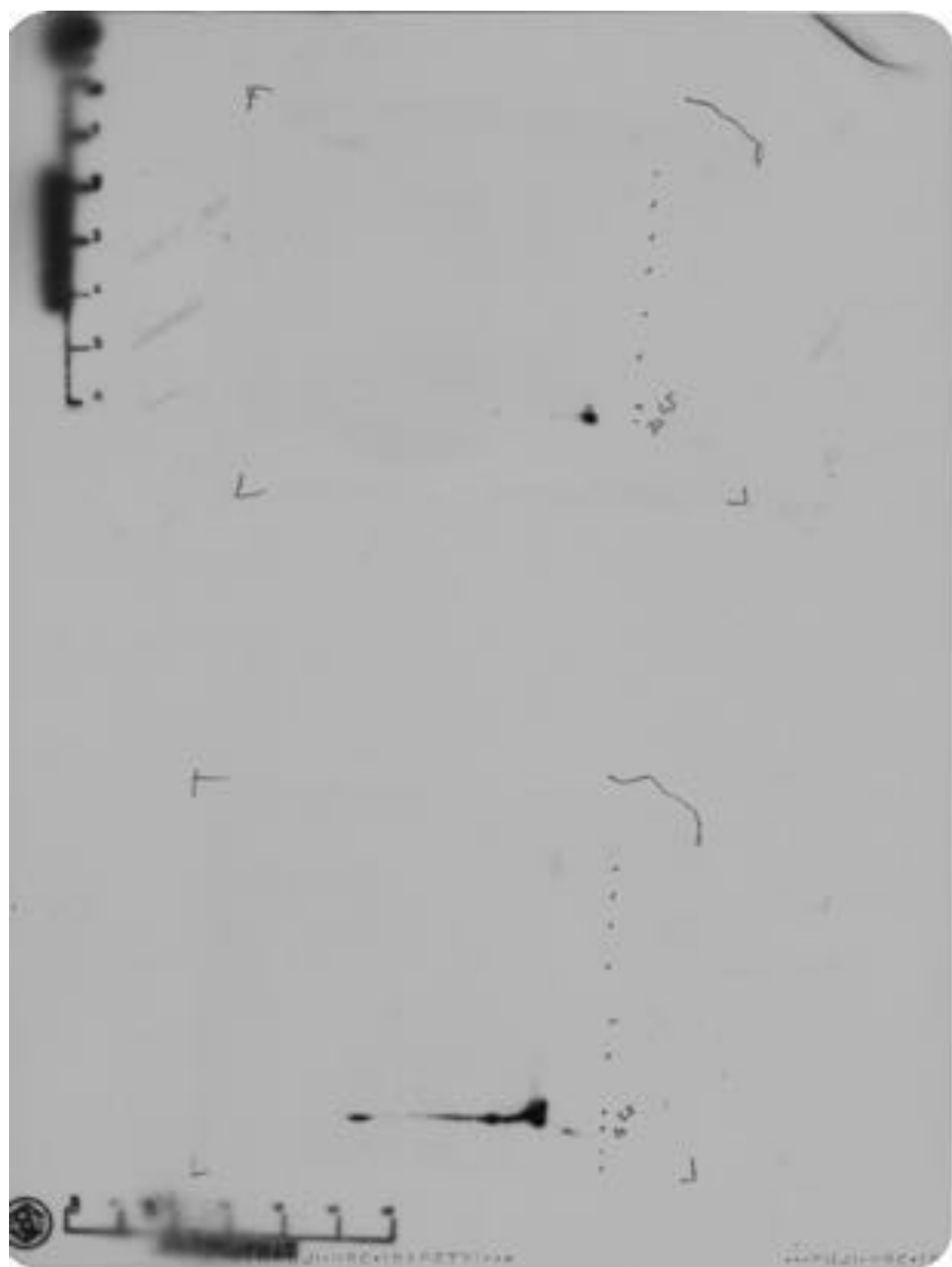

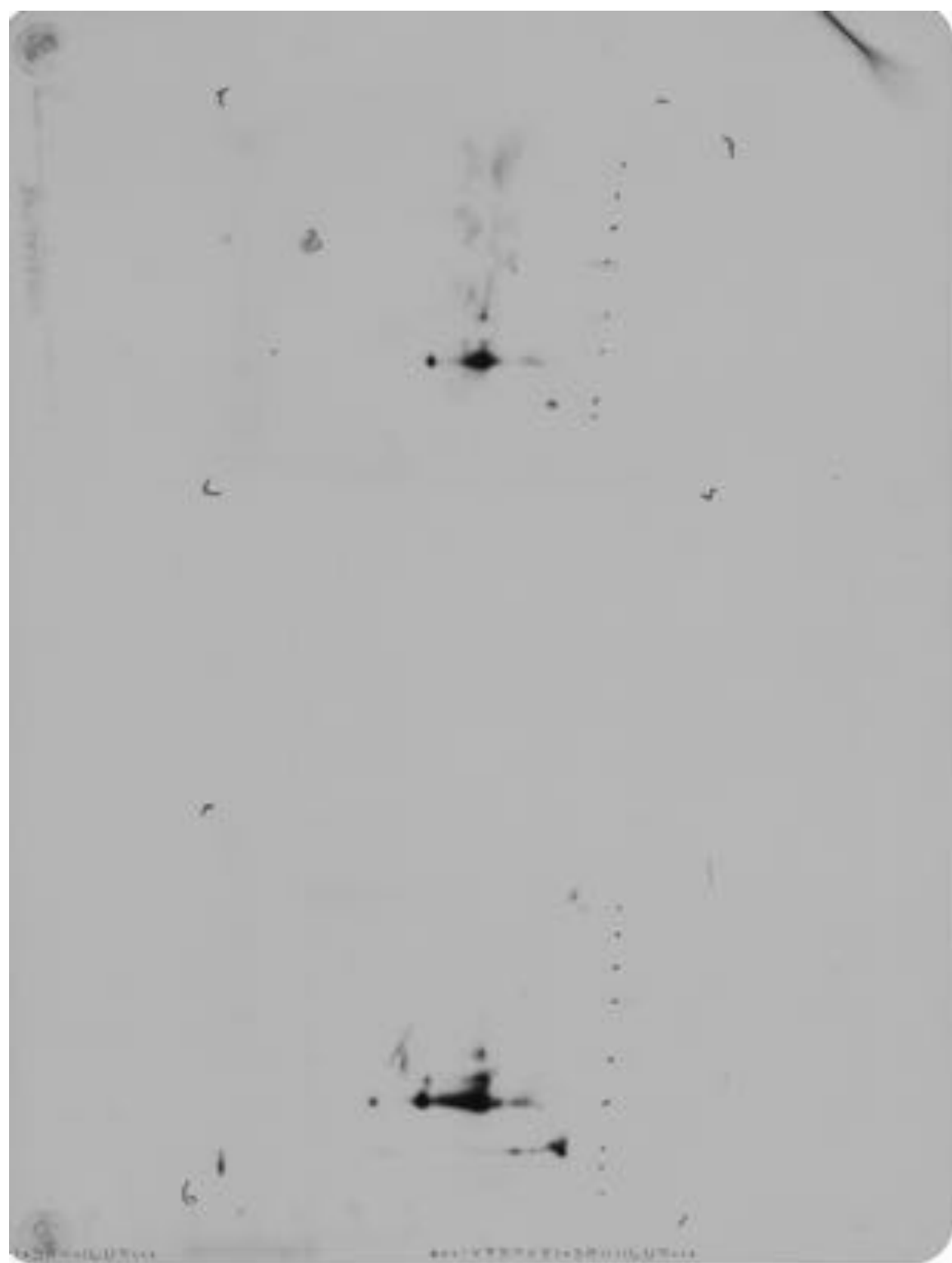

SOLB

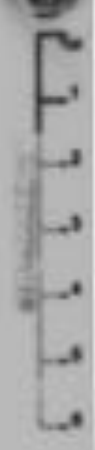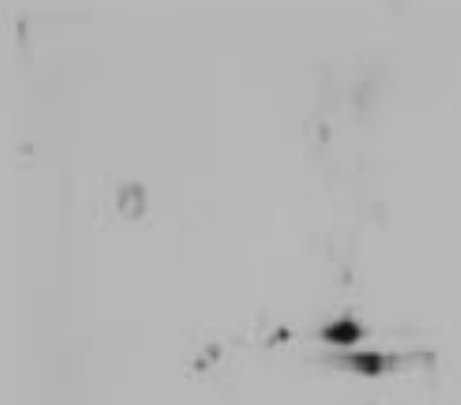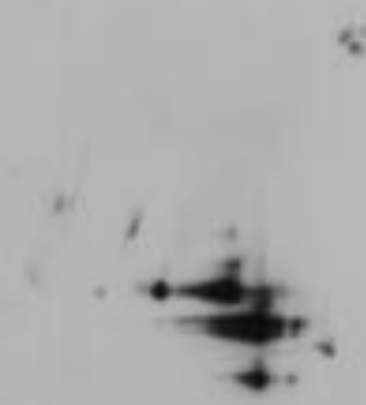

MBB  
Apopt-006EP

UT  
→ GFP

1402  
100µM  
→ GFP

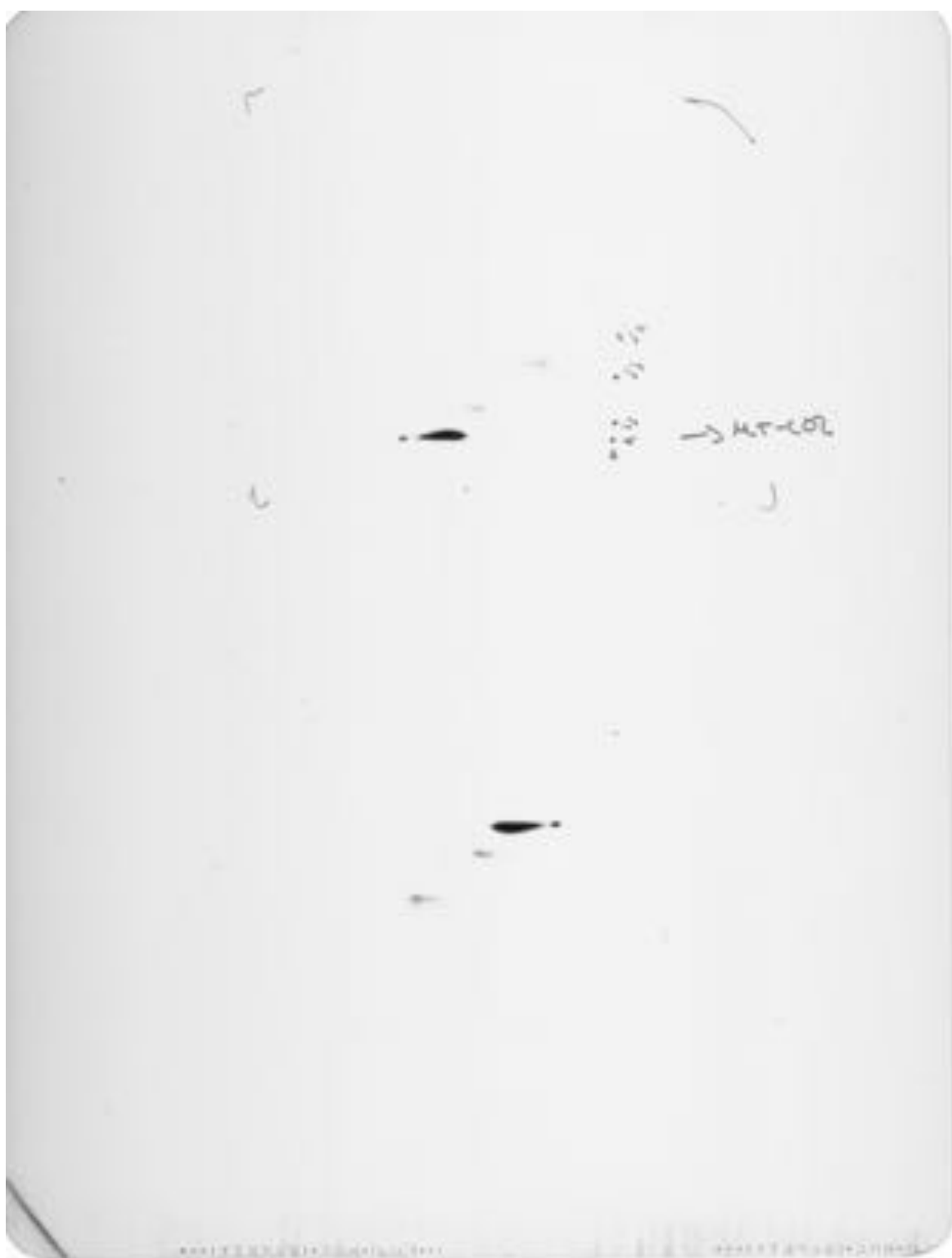

1430  
2001-001-  
600

song

CT

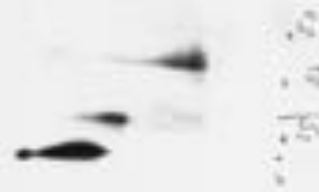

Supplement: Supplementary file 8 — Source Data for Appendix [file EMMM-11-e9582-s007.zip › Appendix_Figure_S5.pdf]

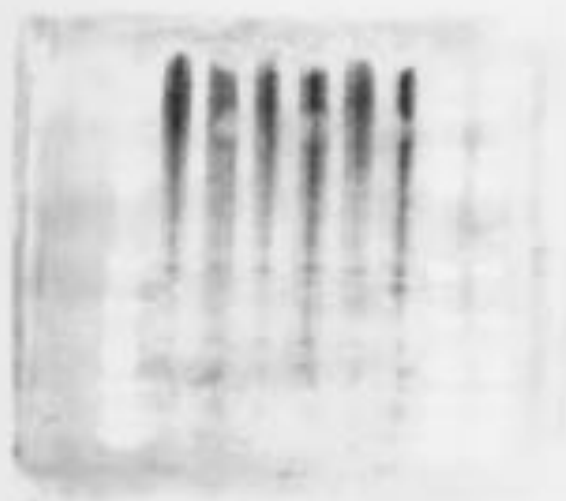

⑤ 子 子 子 子 子 子

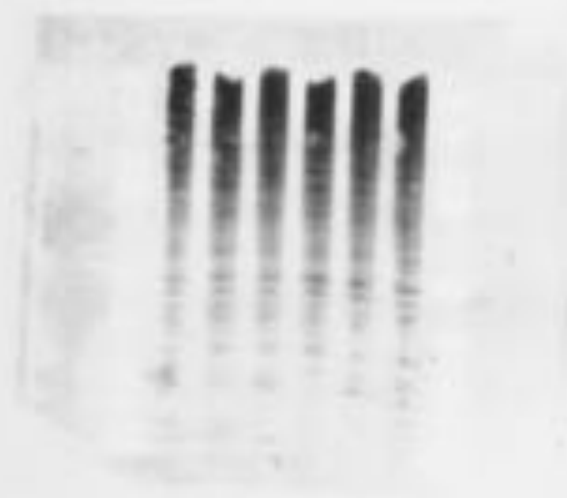

⑥ 子 子 子 子 子 子

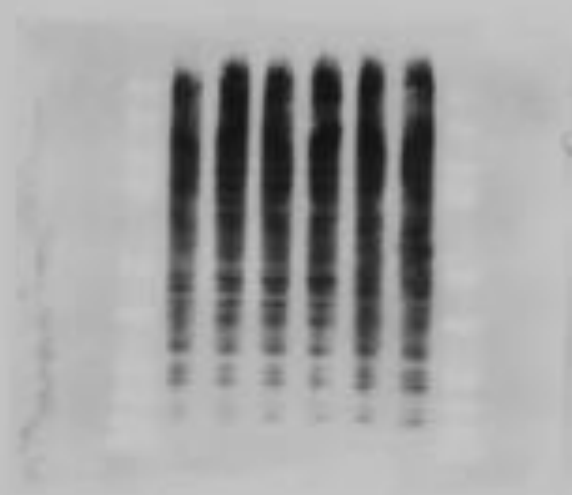

5  
6

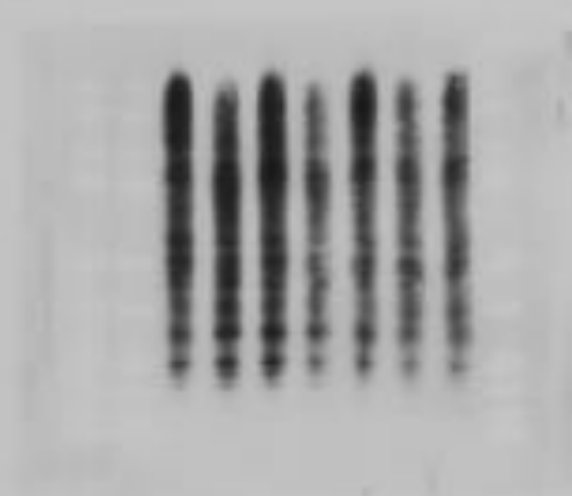

U3iQW7N  
GFP

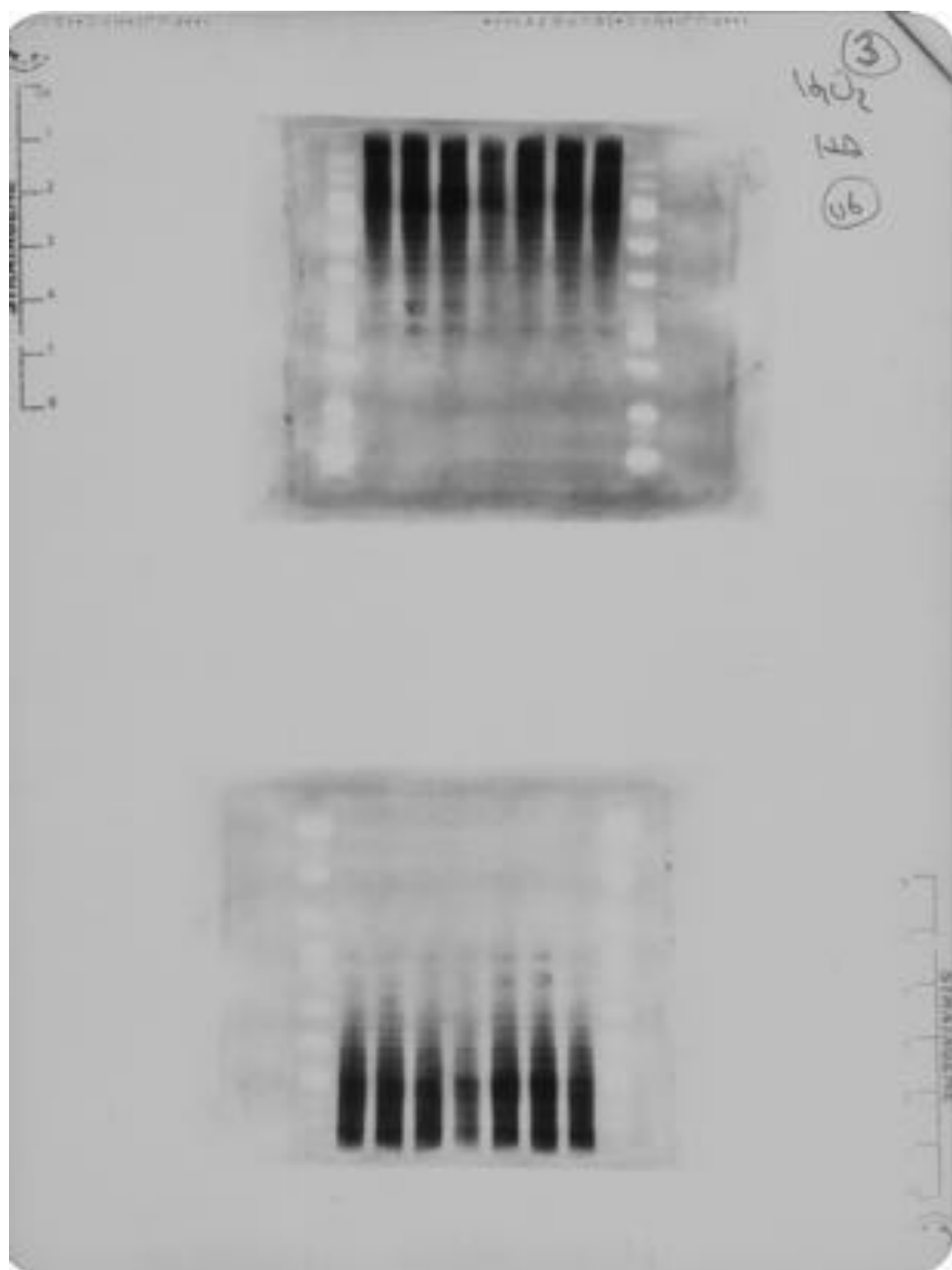

Supplement: Supplementary file 8 — Source Data for Appendix [file EMMM-11-e9582-s007.zip › Appendix_Figure_S8.pdf]
