# Supplementary material for: APOPT1/COA8 assists COX assembly and is oppositely regulated by UPS and ROS
Source: EMBO Mol Med. 2018 Dec 14;11(1):e9582. doi: 10.15252/emmm.201809582 (PMC6328941; doi:10.15252/emmm.201809582)

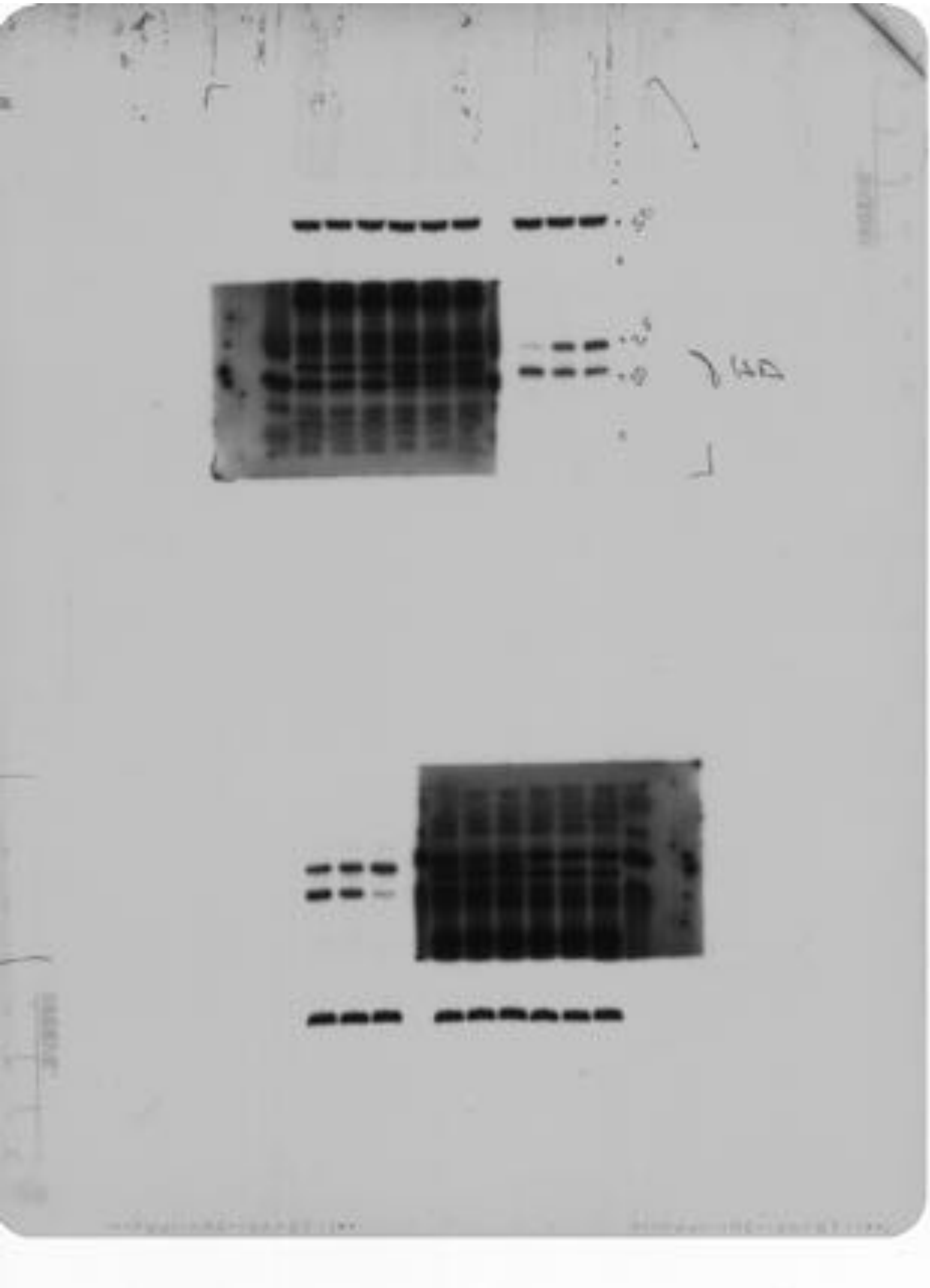

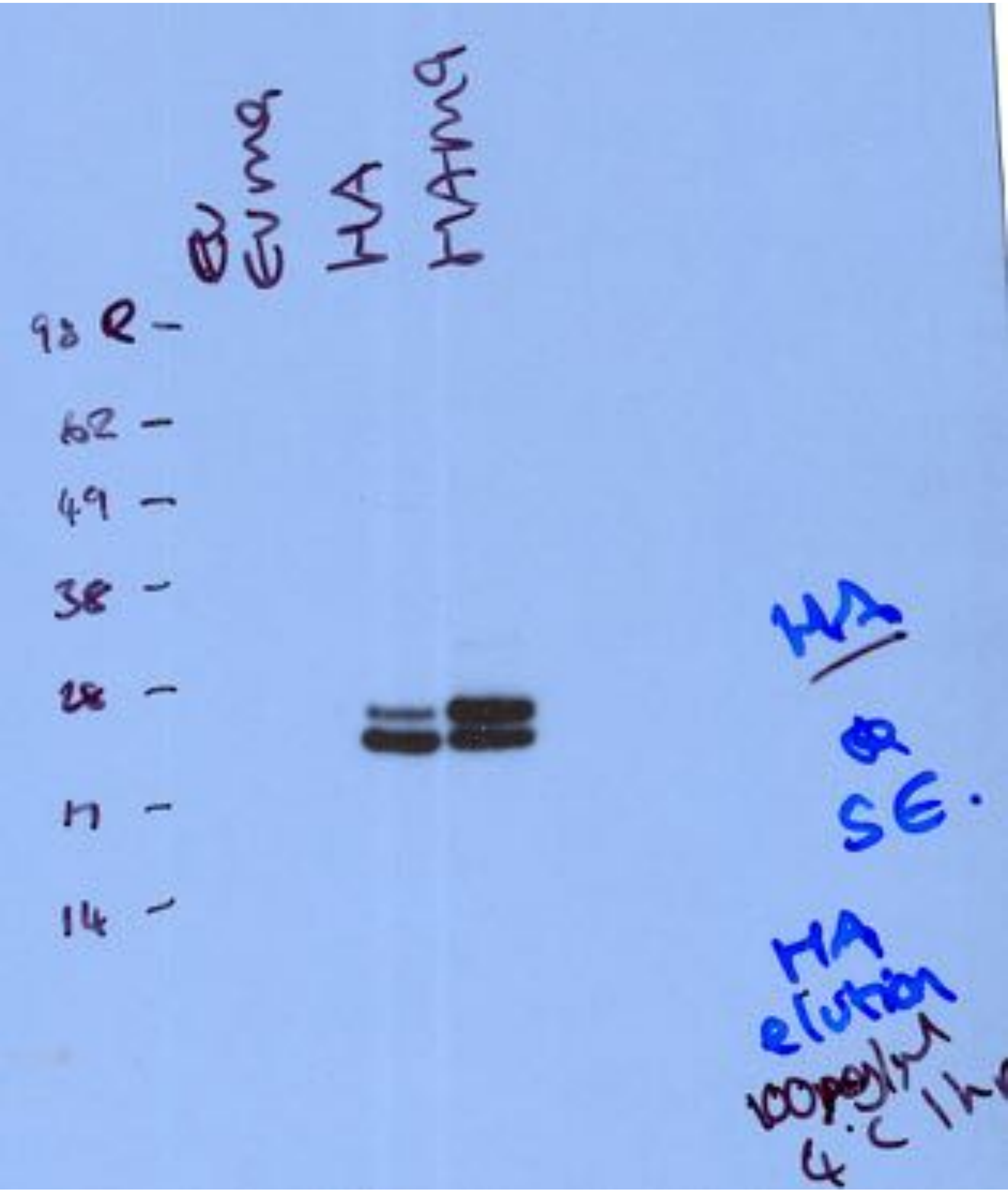
98
62
49
38
17
14
HA
HA
elution
SE.

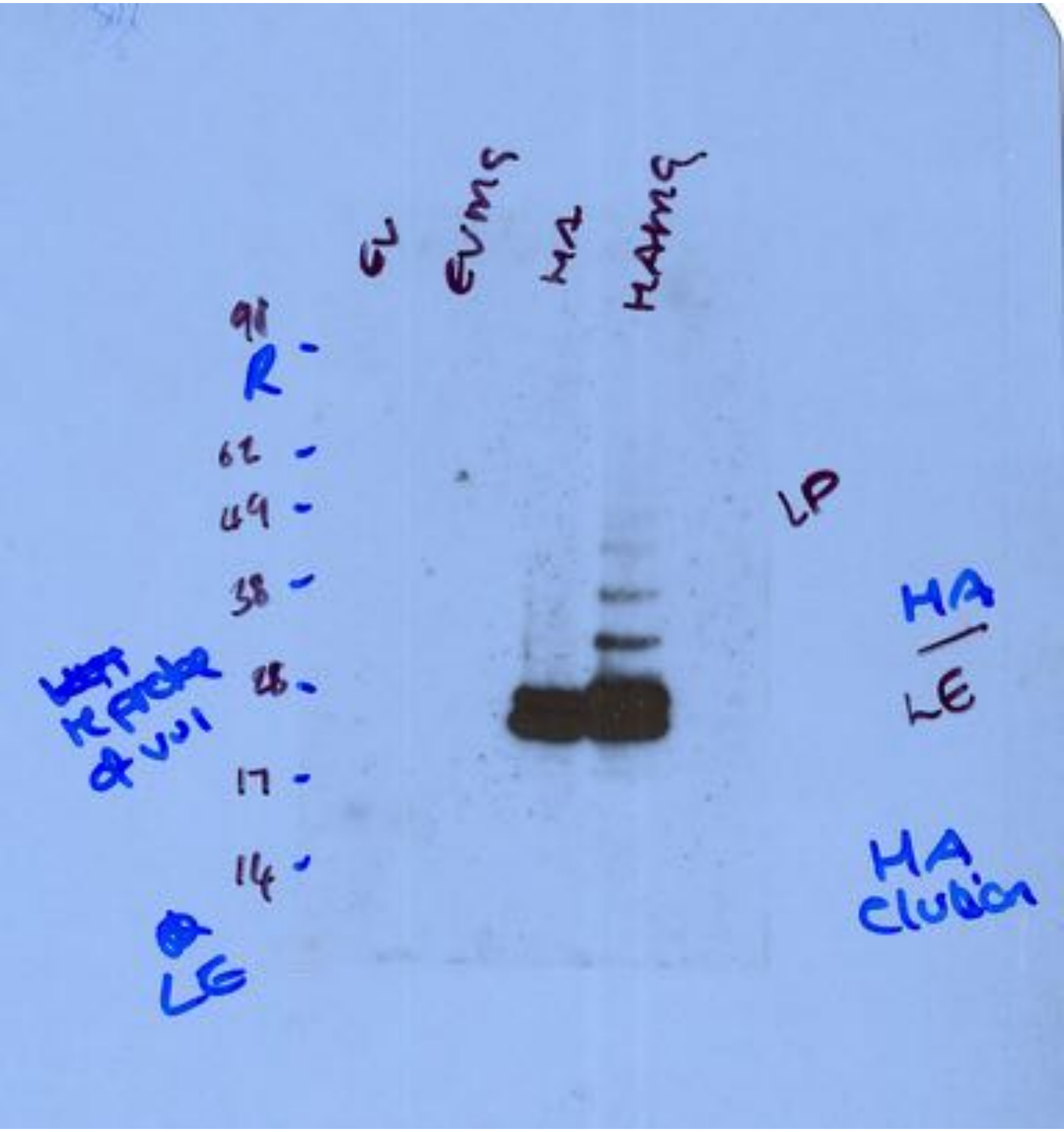

91
R
62
49
38
28
17
14
LP
HA
LE
HA
elution
LG

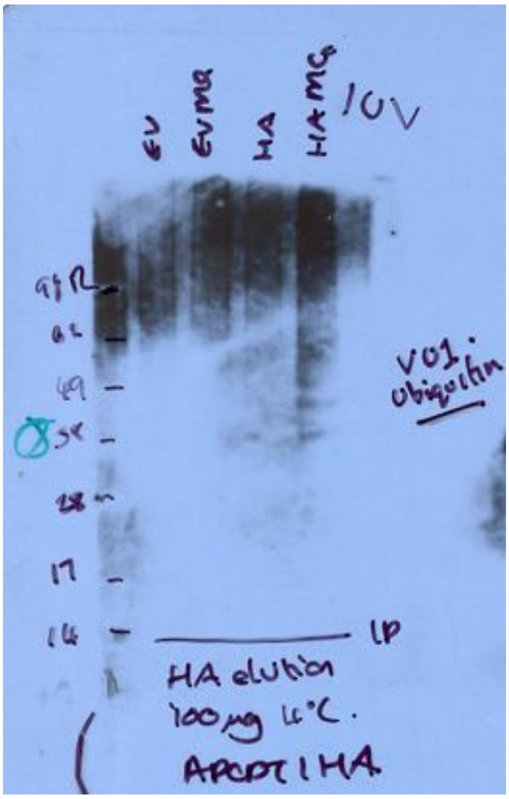

EV
EVMG
HA
HAMG
10V
49
17
IP
HA elution
100μg 4°C.
V01:
ubiquitin

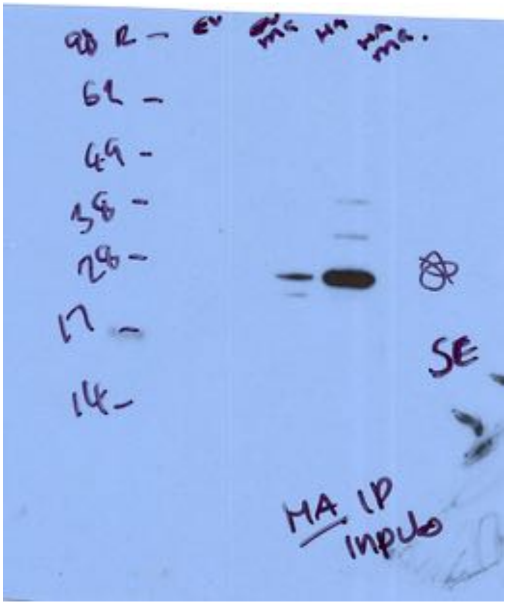

98
62
49
38
28
17
14
EV
HA
SE
HA IP
input

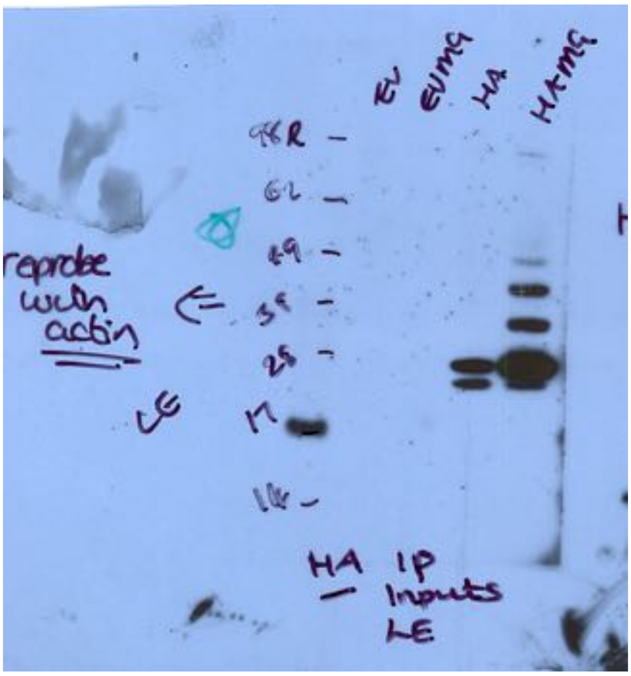
EV
EVMG
HA
HA-MG
98R
62
49
38
28
17
14
reprobe with actin
LE
HA IP
Inputs
LE

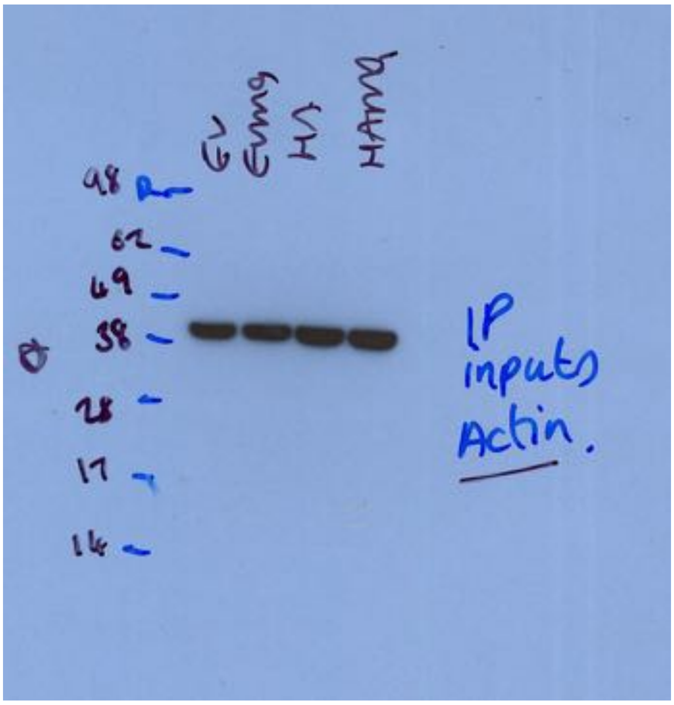
98
62
49
38
28
17
14
EV
Emmg
HA
HAmq
IP
inputs
Actin.

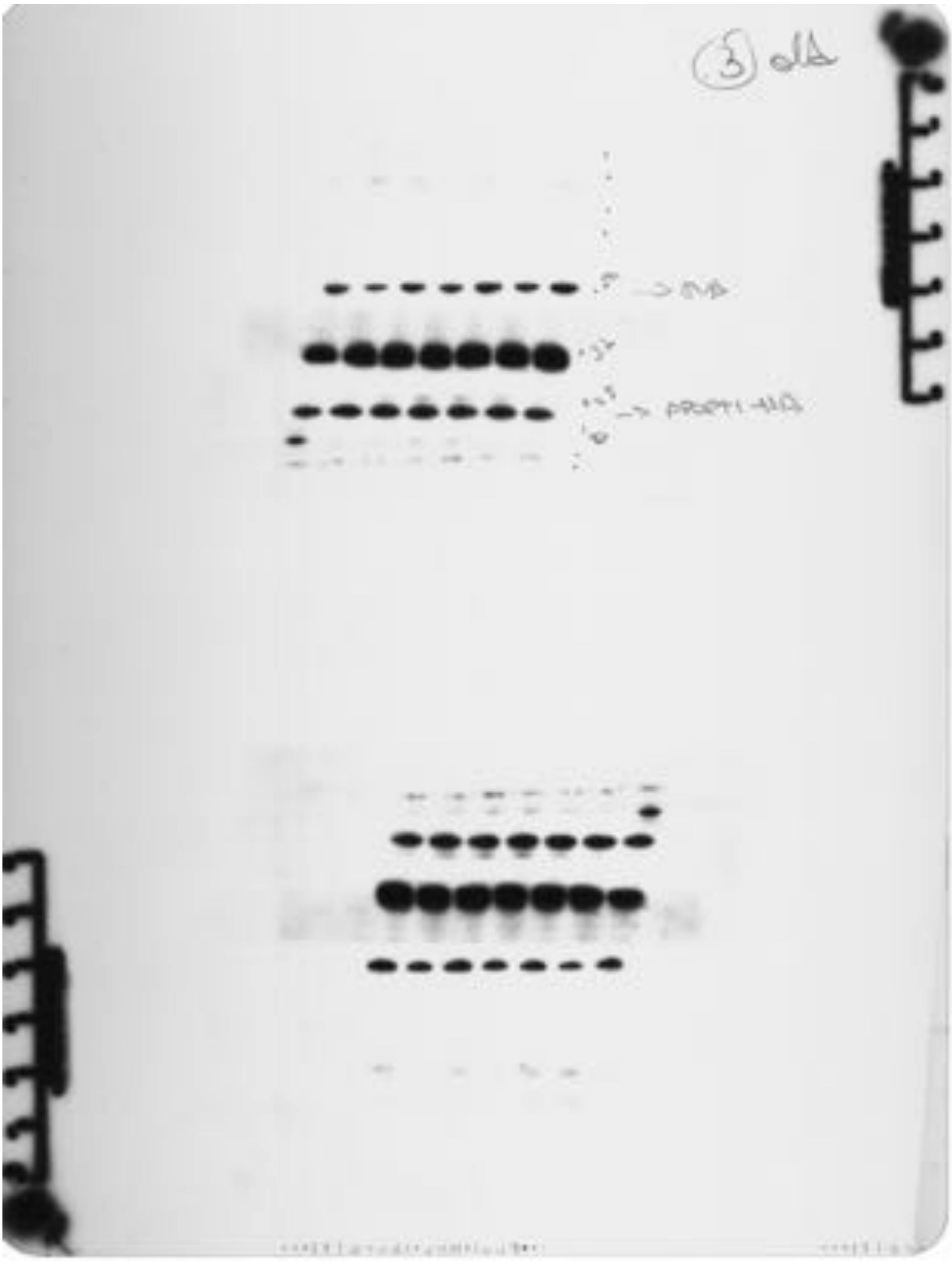
3

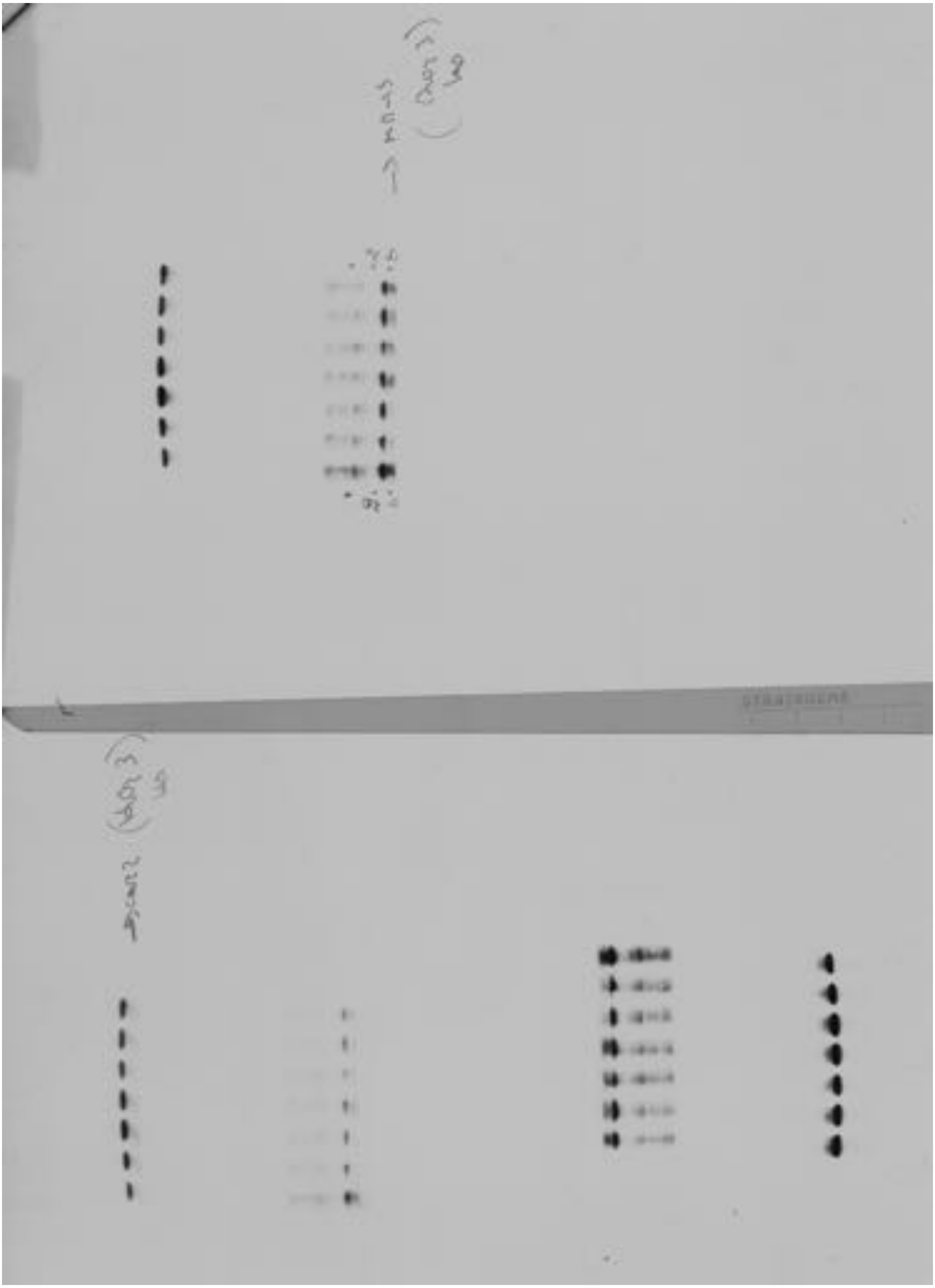

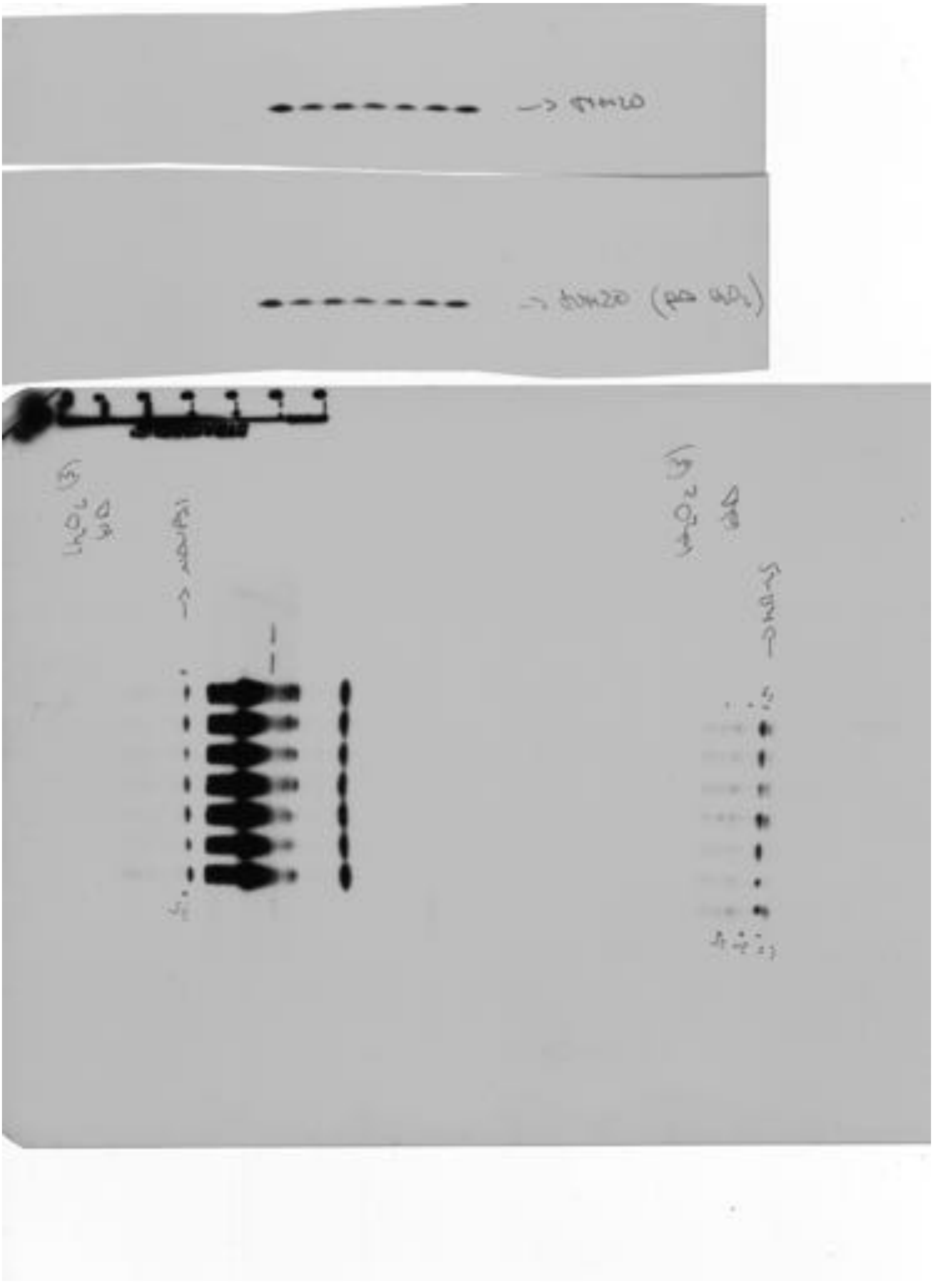

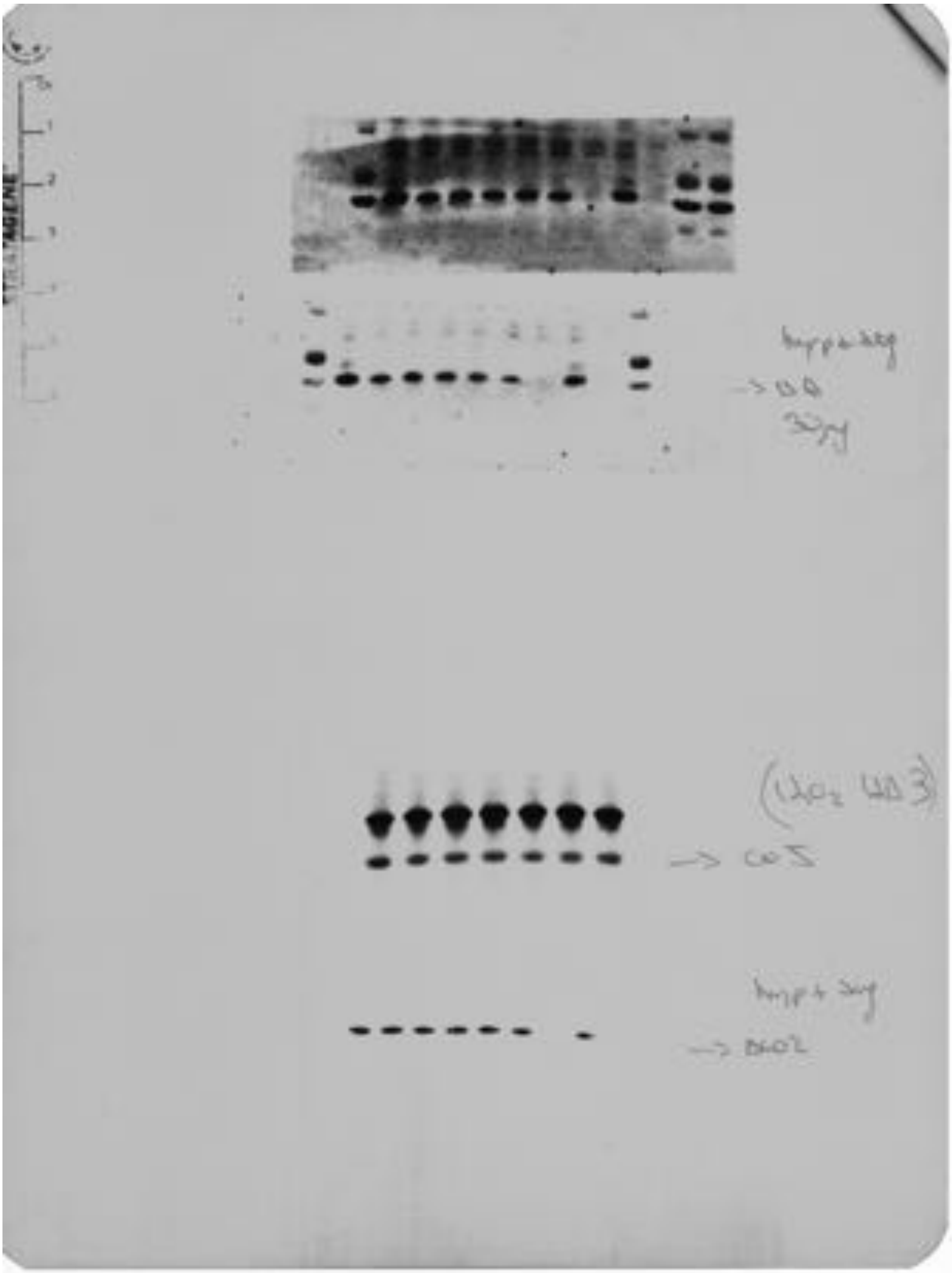

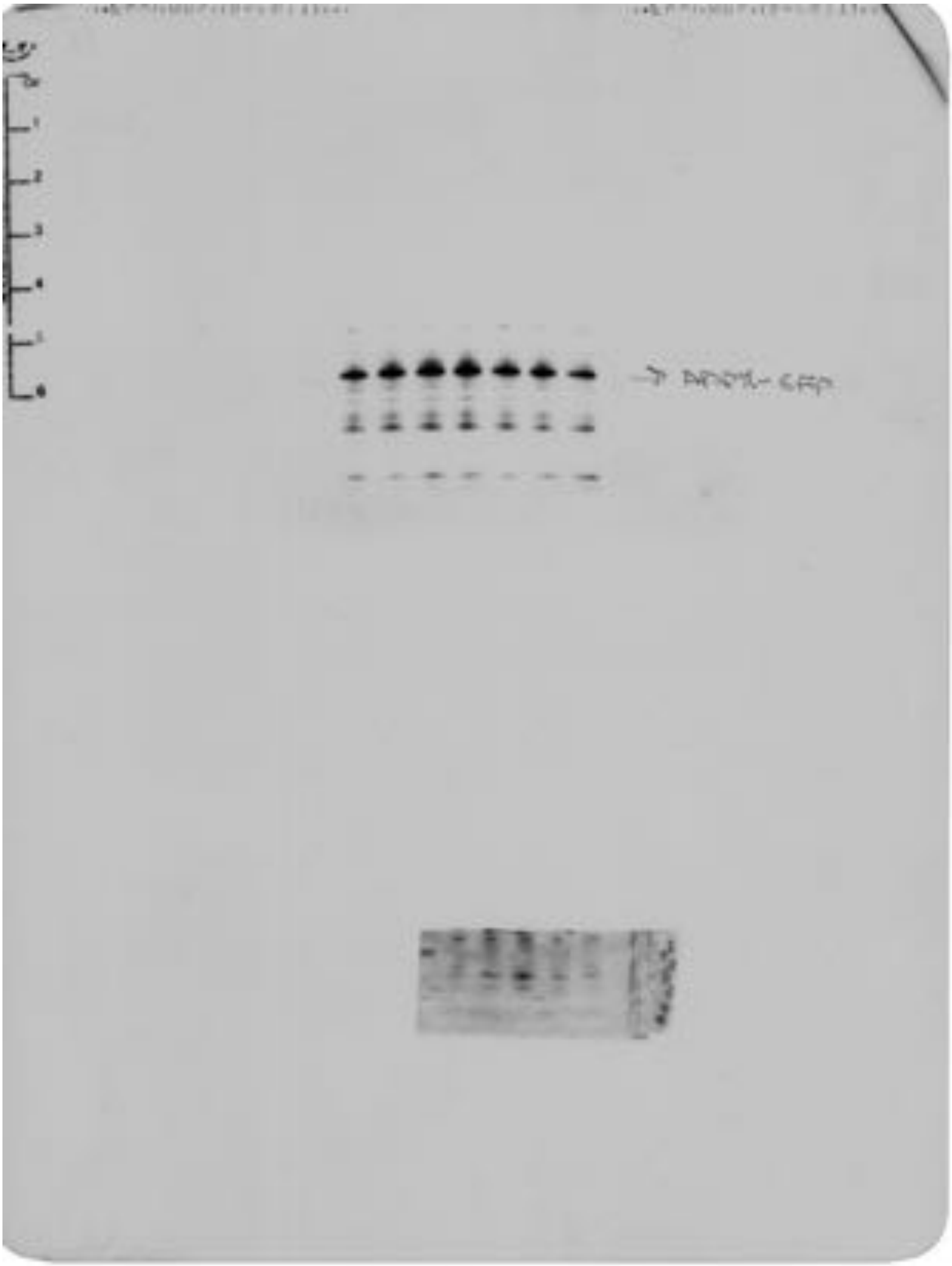

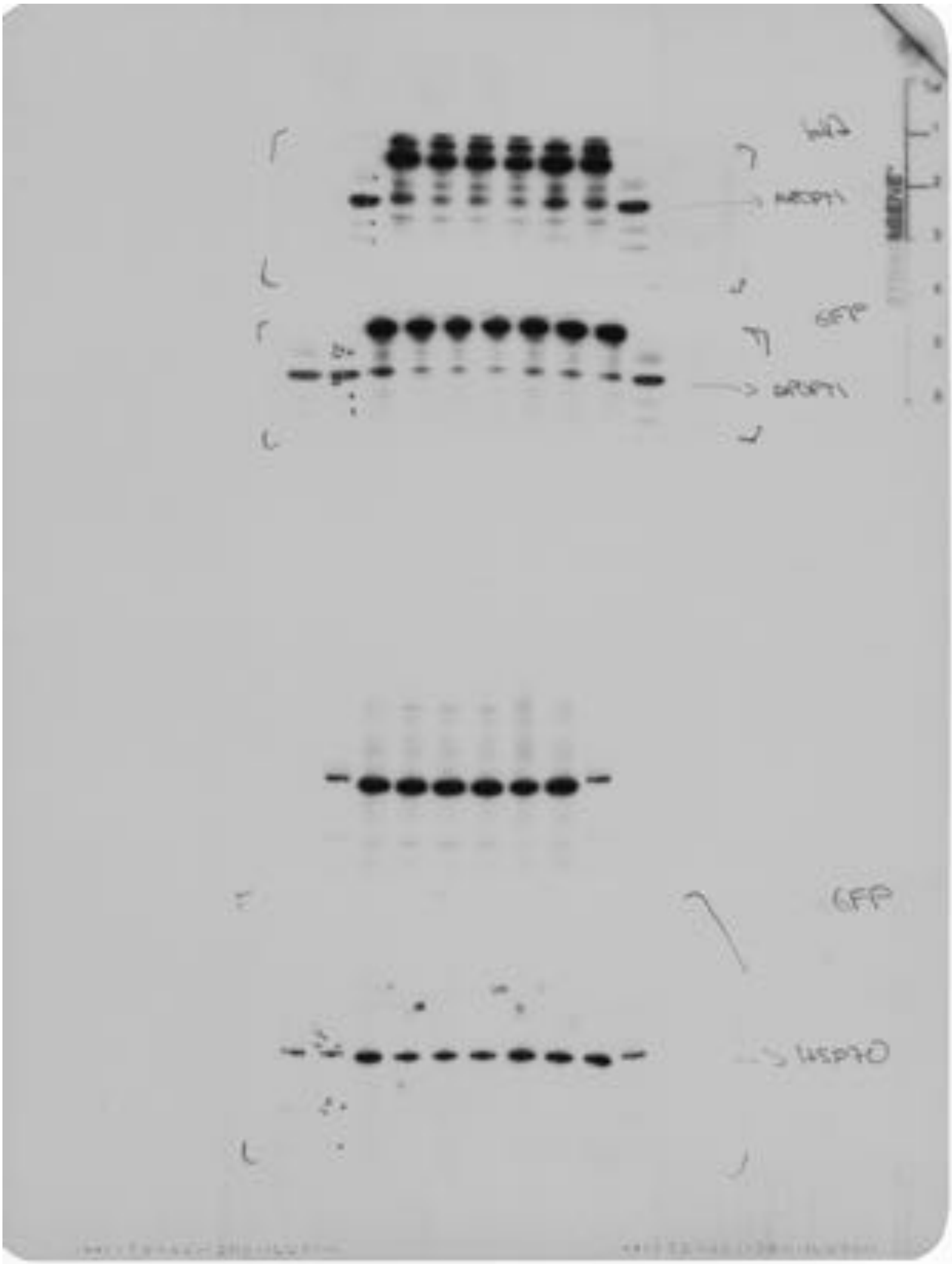

GFP
GFP

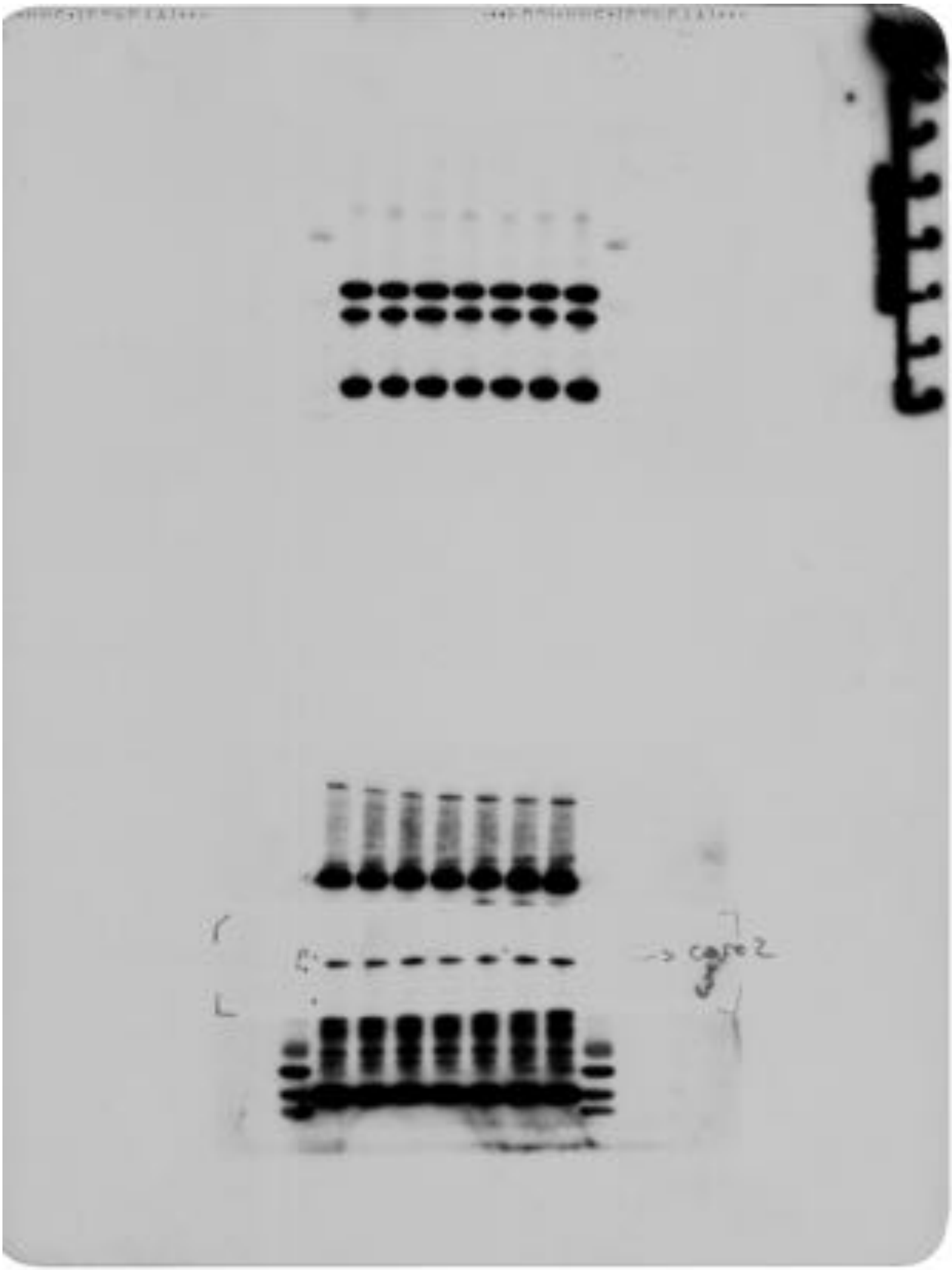
core 2

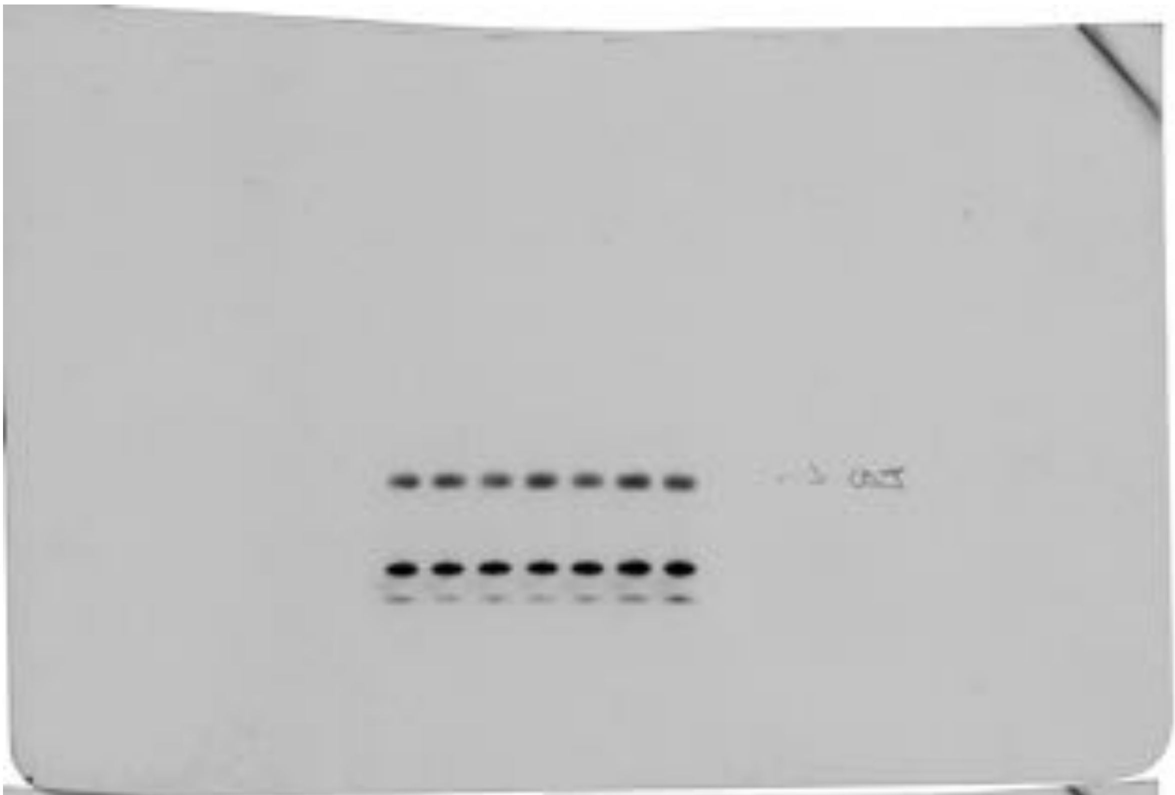

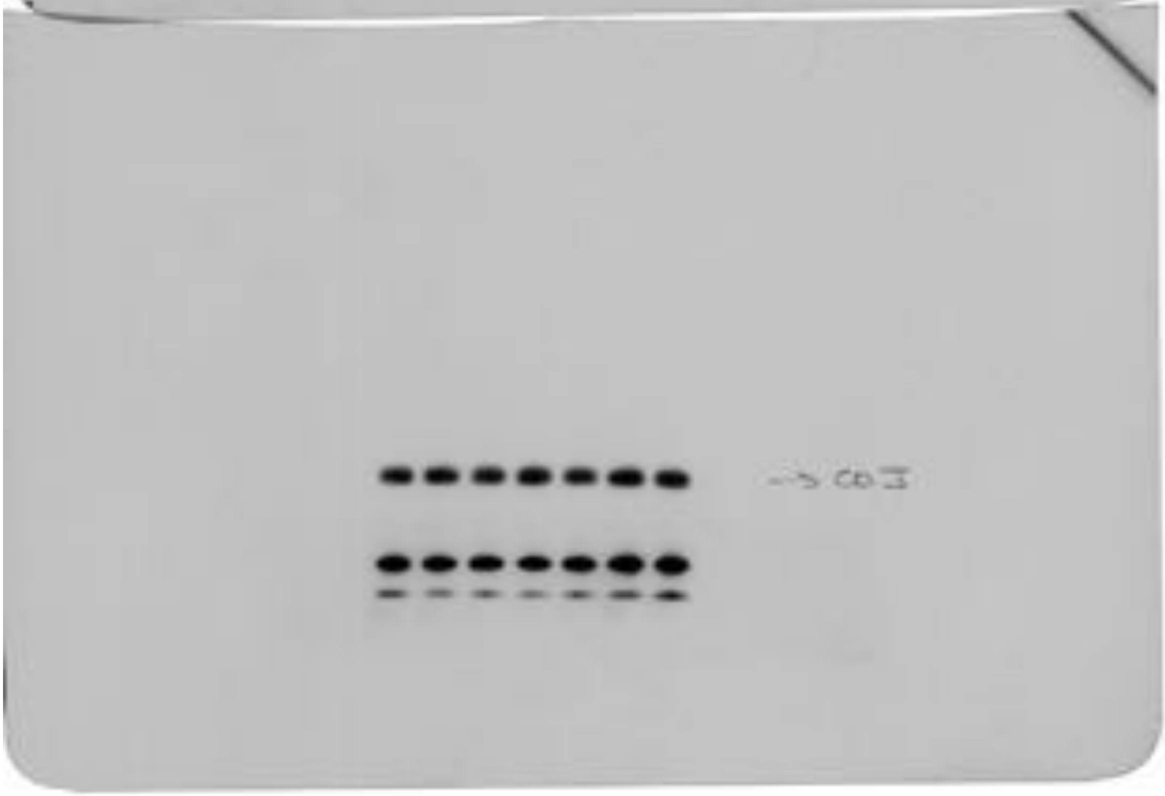

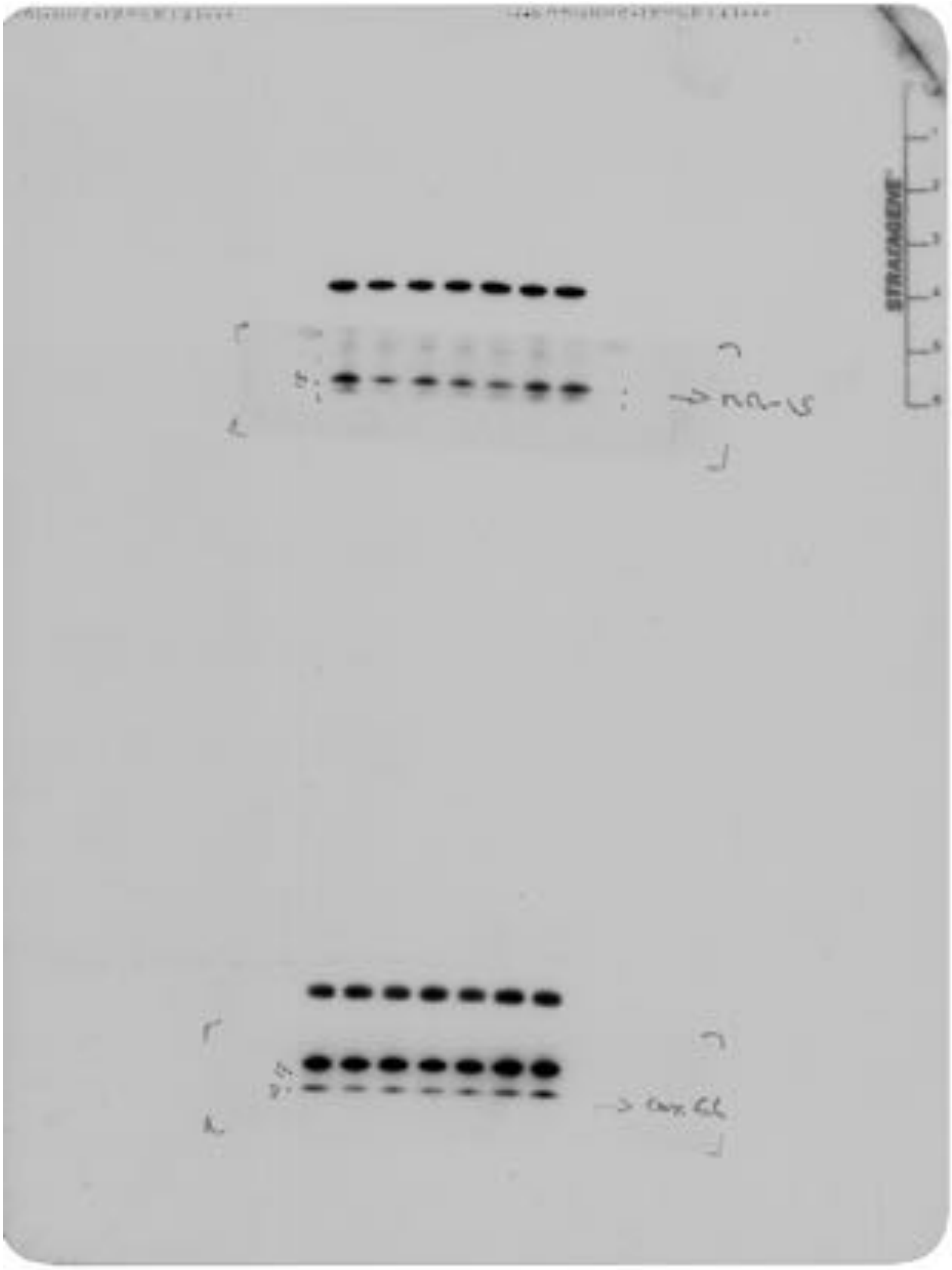
STRATAGENE

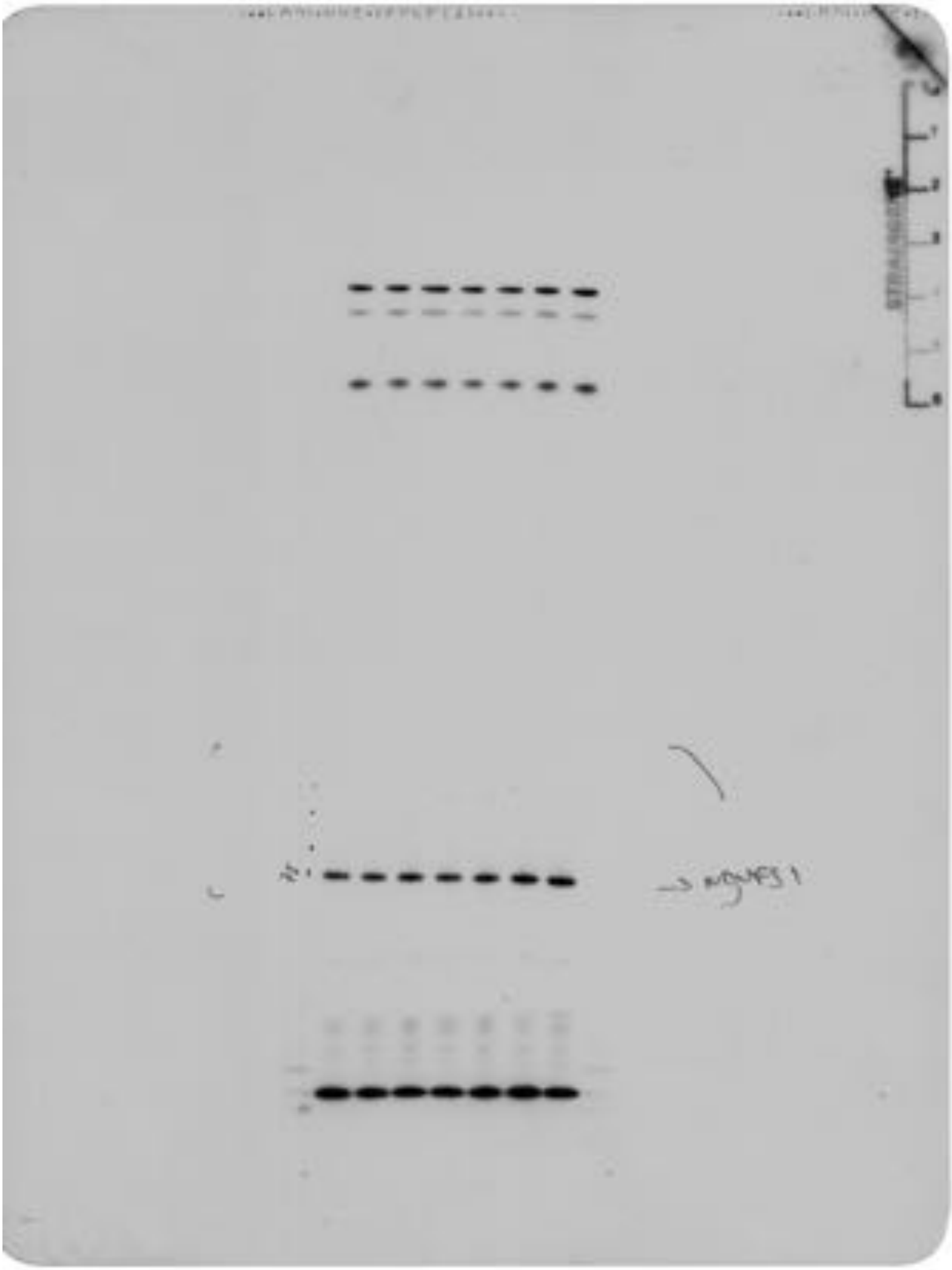

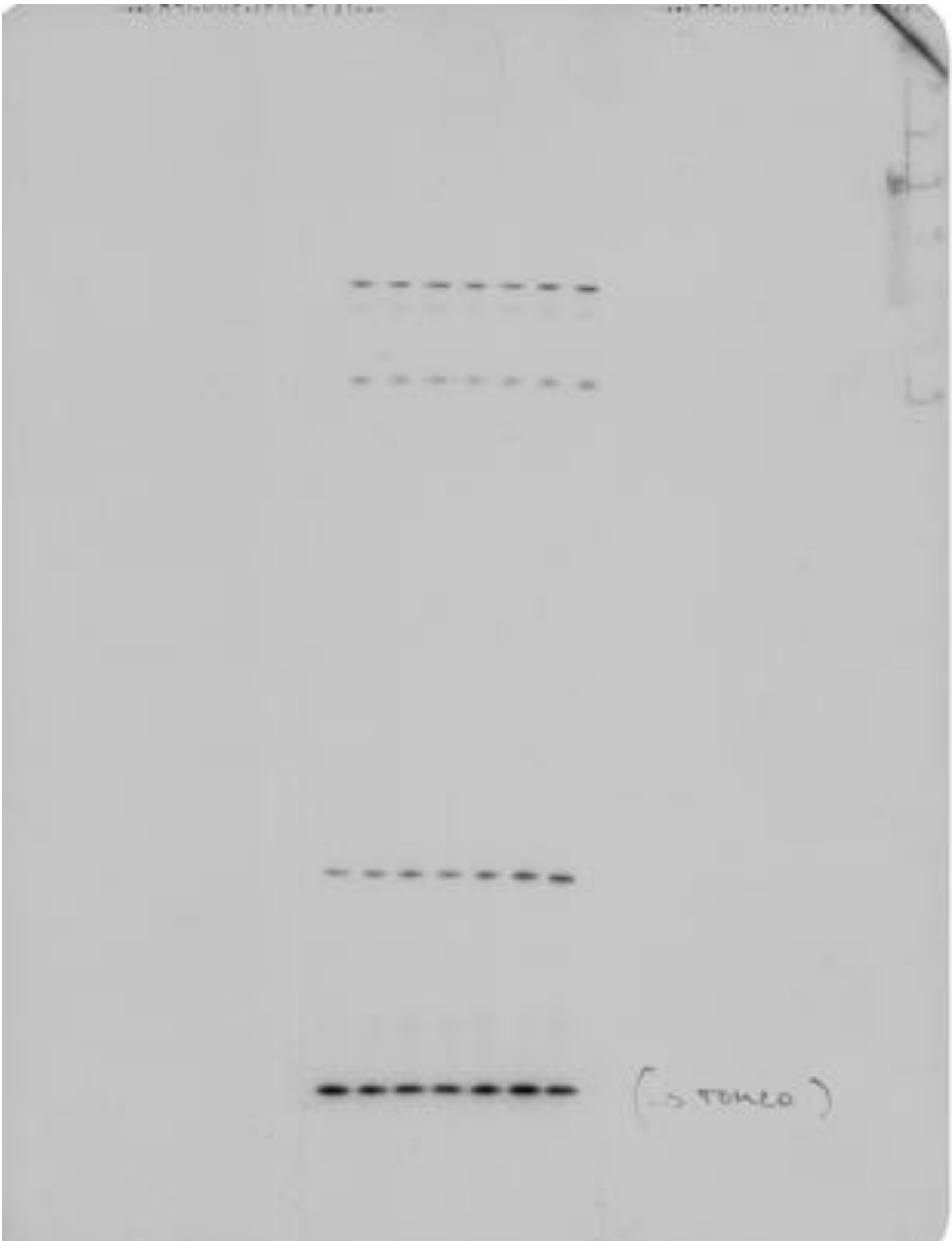

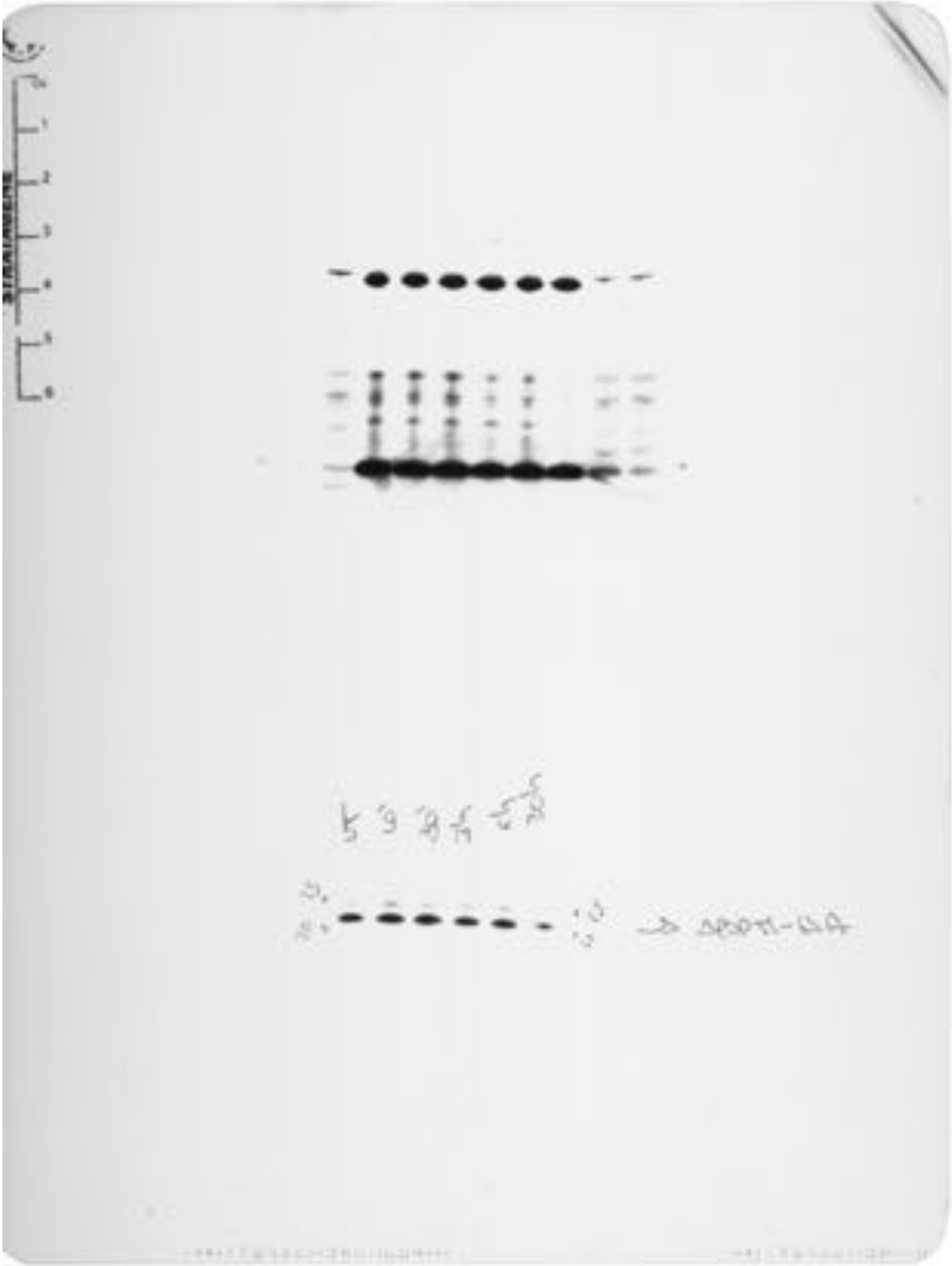

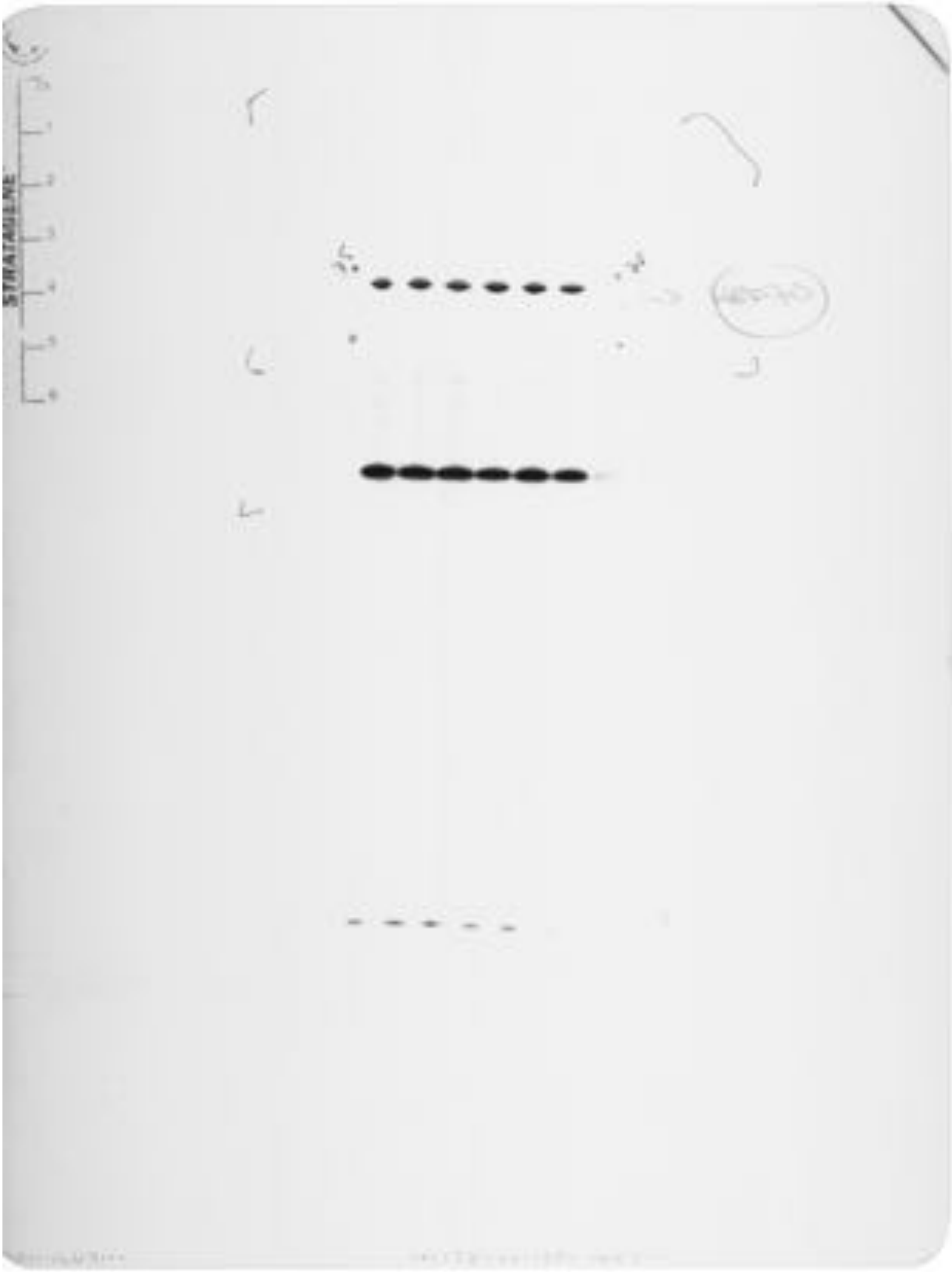
STRATAGENE

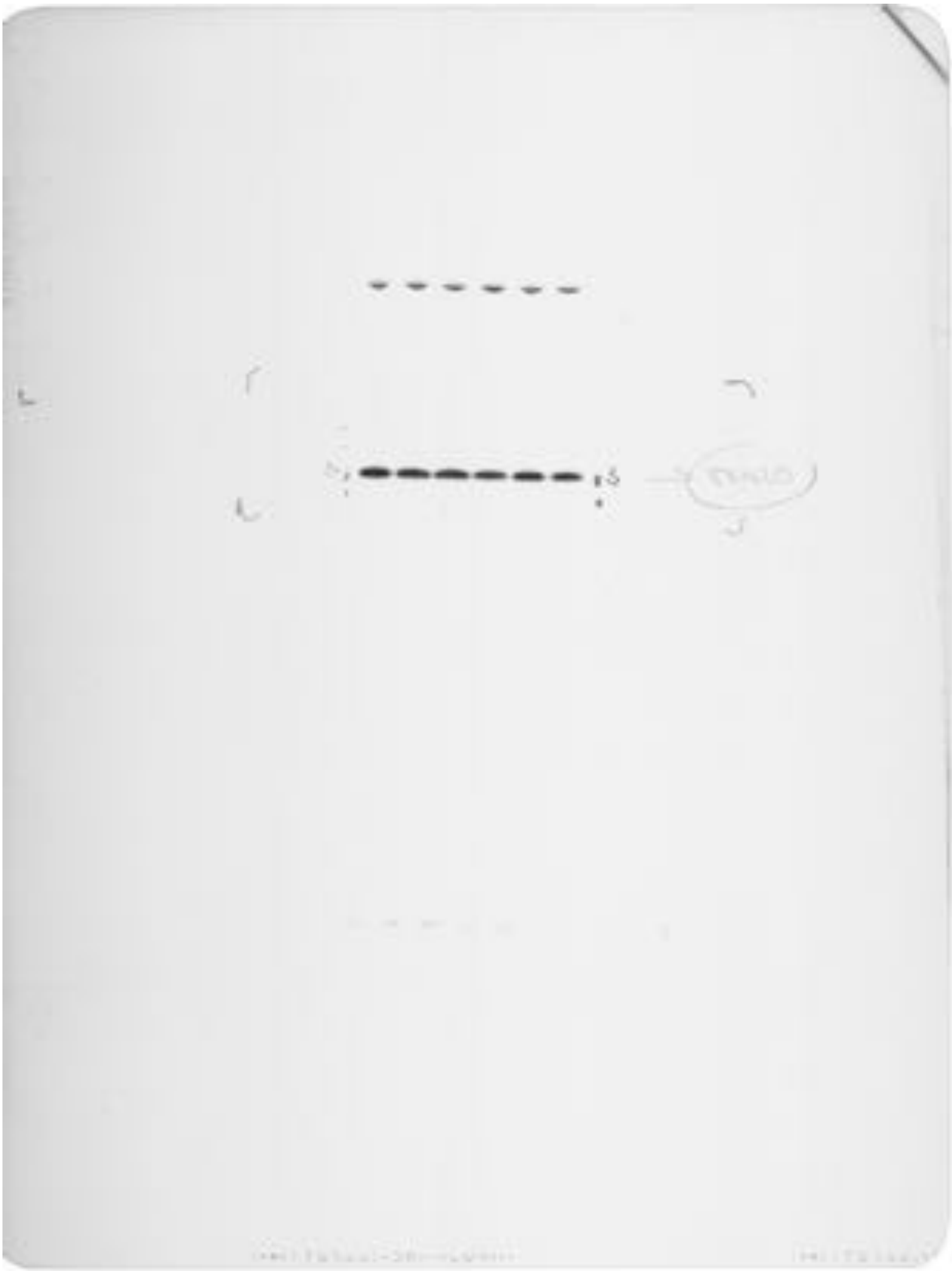

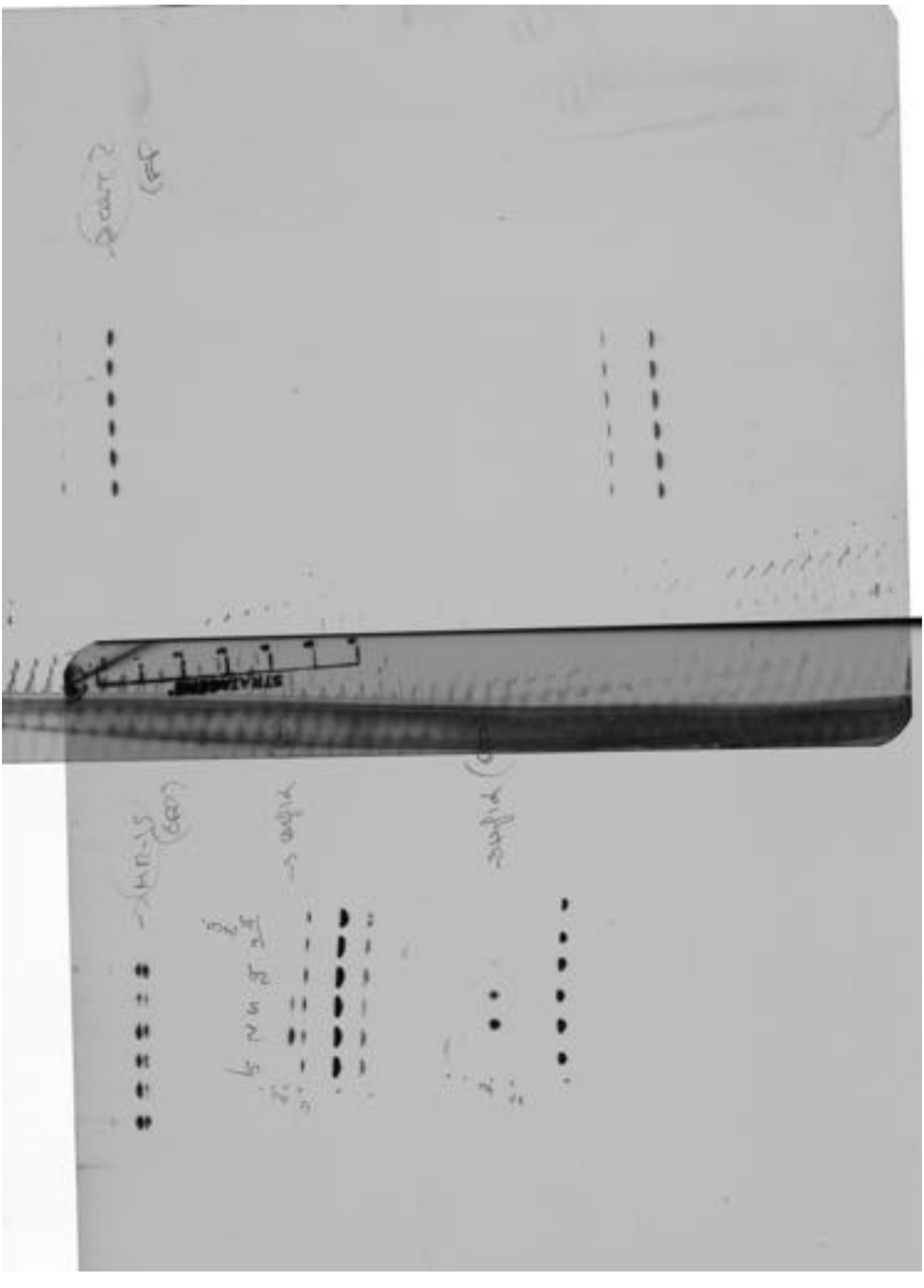

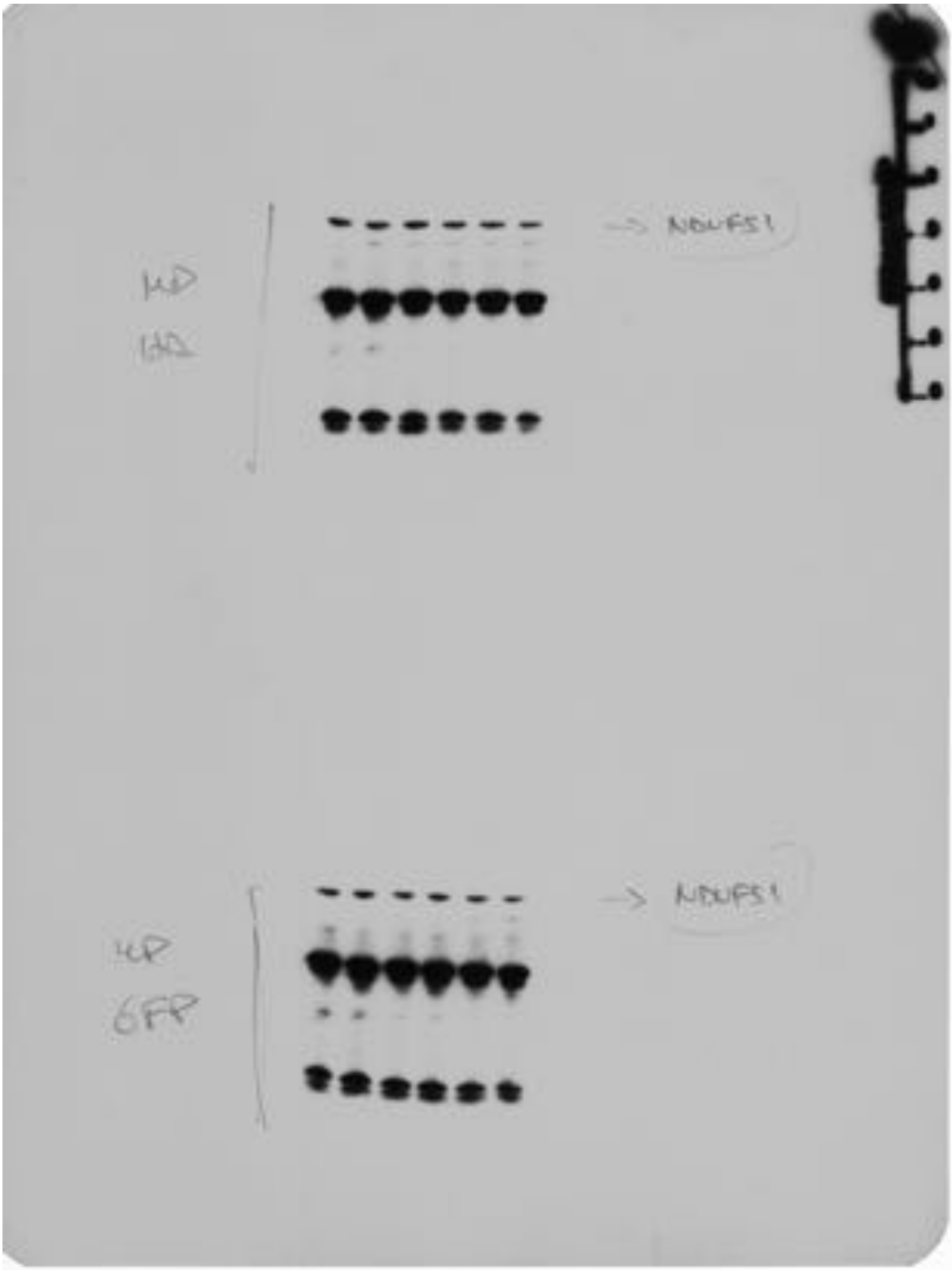

NDUFS1
IP
HA
NDUFS1
IP
GFP

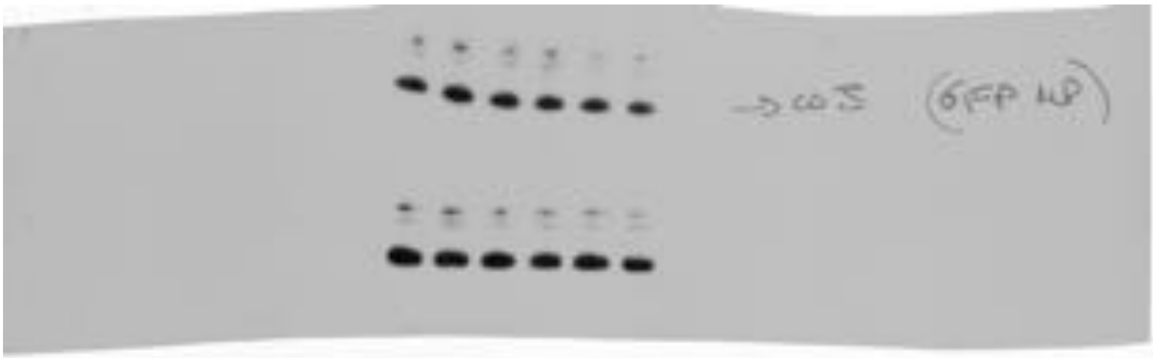
(GFP WB)

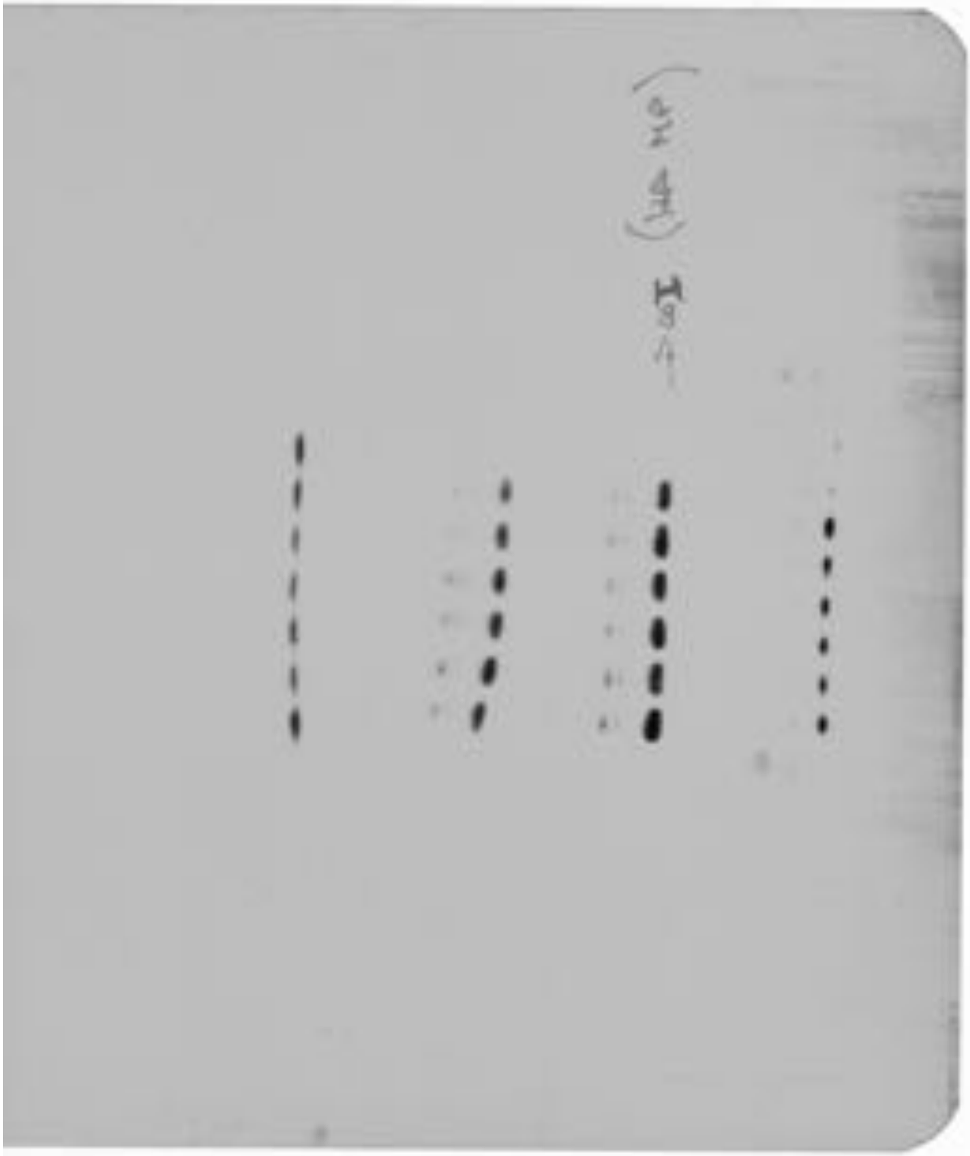

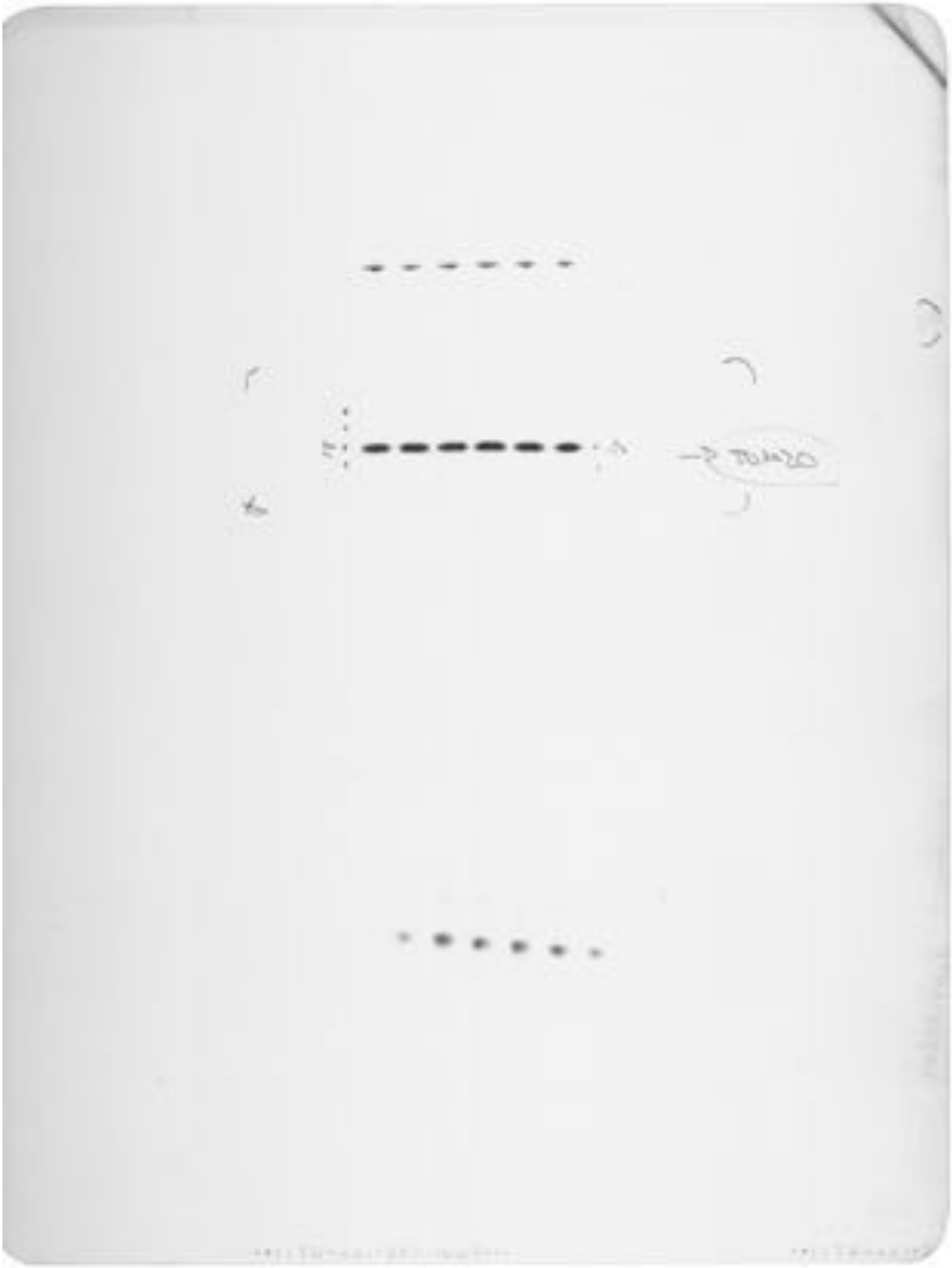

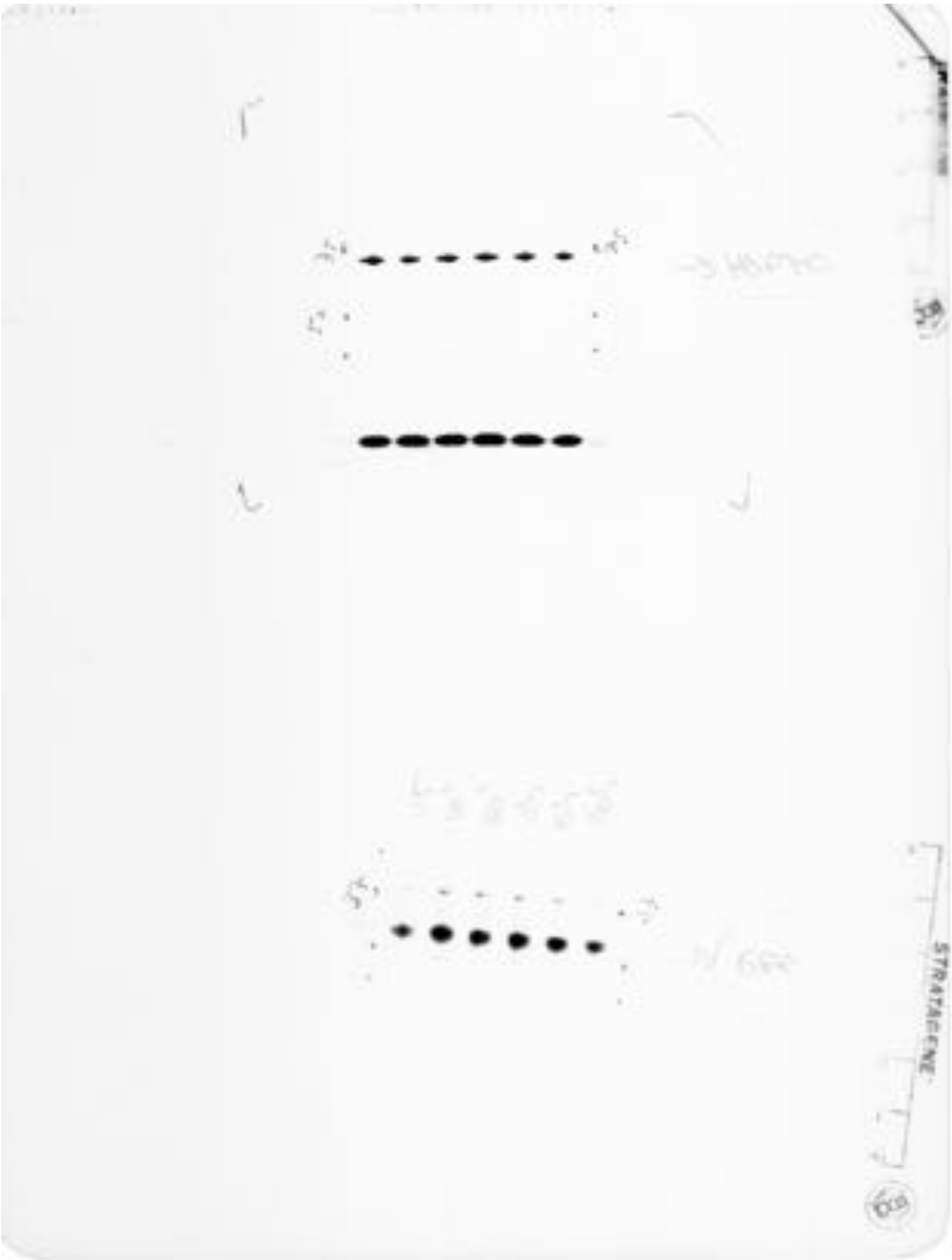
STRATAGENE

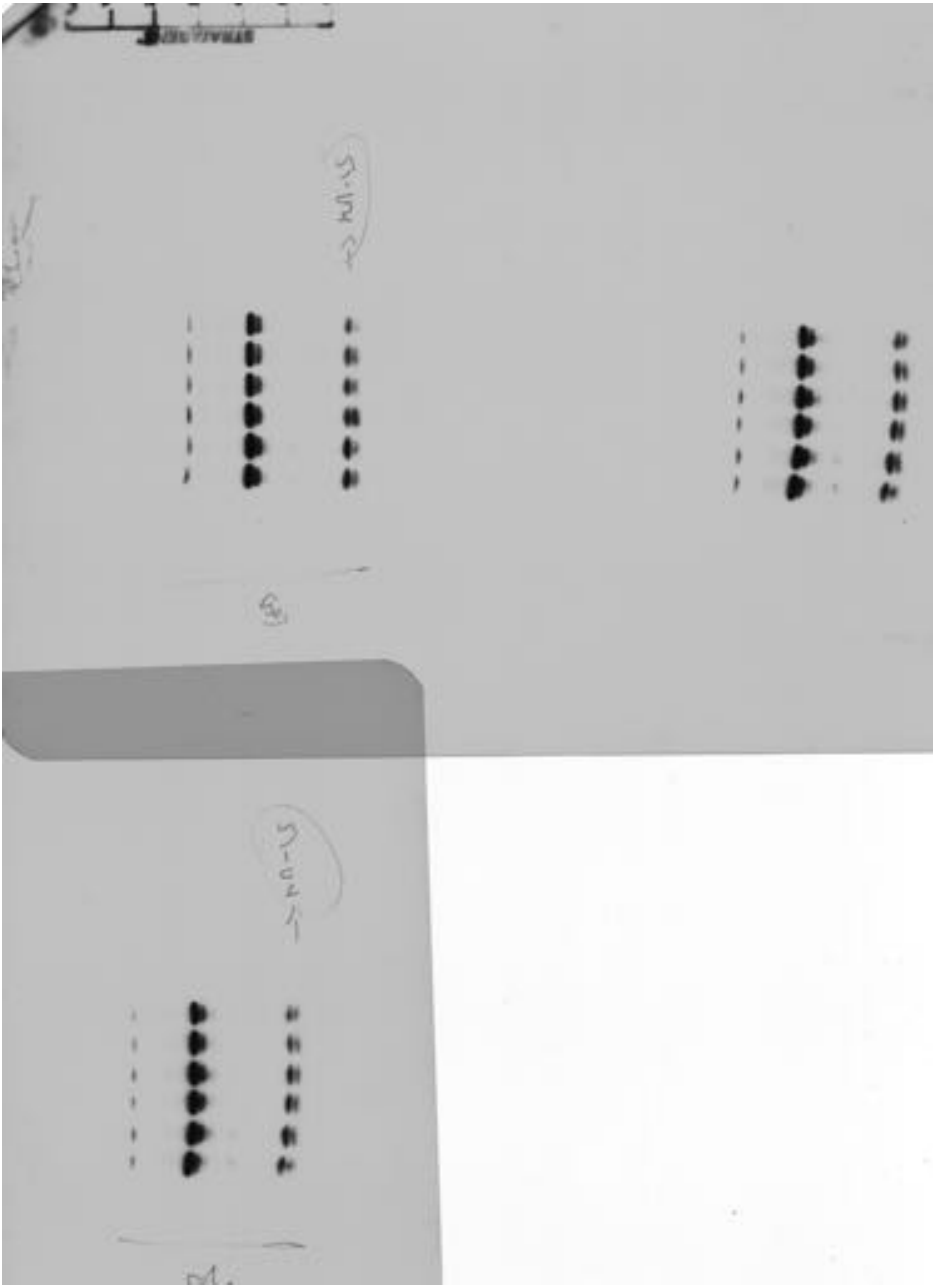

Supplement: Supplementary file 6 — Source Data for Figure 6 [file EMMM-11-e9582-s005.pdf]
